# Supplementary material for: Developmentally regulated activation of defense allows for rapid inhibition of infection in age-related resistance to Phytophthora capsici in cucumber fruit
Source: BMC Genomics. 2020 Sep 11;21:628. doi: 10.1186/s12864-020-07040-9 (PMC7488727; doi:10.1186/s12864-020-07040-9)
Supplement: Supplementary file 5 — Additional file 5: Supplementary file 5. All code and analysis [file 12864_2020_7040_MOESM5_ESM.pdf]

Developmentally regulated activation of defense allows for rapid inhibition of infection in age-related resistance to *Phytophthora capsici* in cucumber fruit

Supplementary File 5: Analysis workflow

Ben N. Mansfeld, Marivi Colle, Chunqiu Zhang, Ying-Chen Lin, Rebecca Grumet

04/07/2019

```
# source("https://bioconductor.org/biocLite.R")
# biocLite("tximport")
# bioCLite("impute")
library(tidyverse)
library(UpSetR)
library(grid)
library(tximport)
library(stringr)
library(readr)
library(DESeq2)
library(kableExtra)
library(rjson)
library(ggalluvial)
library(WGCNA)
library(pheatmap)
library(RColorBrewer)
library(splines)
library(rvest)
library(readxl)
library(magick)
library(topGO)
```

Physiology and other experiments

Disease rating vs. growth

```
loaded <- read.csv(file = "diseaserating_length_marivi.csv", header = TRUE)

drPlot <- ggplot(loaded) +
    stat_summary(
        aes(x = Age, y = `Disease.rating`, linetype = "Disease rating"),
        fun.y = mean,
        geom = "line",
        na.rm = TRUE,
        size = 1
    ) +
```

```
    stat_summary(
        aes(x = Age, y = `Disease.rating`),
        fun.y = mean,
        geom = "point",
        na.rm = TRUE,
        size = 2
    ) +
    stat_summary(
        aes(x = Age, y = `Disease.rating`),
        fun.data = mean_se,
        geom = "errorbar",
        na.rm = TRUE,
        width = 0.2
    ) +
    stat_summary(
        aes(
            x = Age,
            y = `Length` / 2.777,
            linetype = "Fruit length"
        ),
        fun.y = mean,
        geom = "line",
        na.rm = TRUE,
        size = 1
    ) +
    stat_summary(
        aes(x = Age, y = `Length` / 2.777),
        fun.y = mean,
        geom = "point",
        na.rm = TRUE,
        size = 2
    ) +
    stat_summary(
        aes(x = Age, y = `Length` / 2.777),
        fun.data = mean_se,
        geom = "errorbar",
        na.rm = TRUE,
        width = 0.2
    ) +
    scale_y_continuous(
        name = "Disease rating (1-9)",
        sec.axis = sec_axis( ~ . * 2.777, name = "Length (cm)"),
        limits = c(1, 9)
    ) +
    scale_linetype() +
    geom_hline(yintercept = 3, linetype = 2) +
    cowplot::theme_cowplot(font_size = 14) +
    theme(
        legend.title = element_blank(),
        legend.position = c(0.7, 0.1),
        legend.direction = "vertical"
    )
```

Fluorecent plate reader data:

```r
rawData <- readxl::read_xlsx(path = "flu_2018-10-6_raw_data.xlsx",
                             sheet = 1,
                             skip = 61,
                             n_max = 1822,
                             col_names = FALSE)

wells <- rawData[seq(from = 1, to = nrow(rawData), by = 19), 1, drop = TRUE]
time <- as.numeric(rawData[2, 2:ncol(rawData)])

df <- rawData[rep(seq(from = 0, nrow(rawData) - 1, by = 19), each = 12) + 6:17, ]
df <- cbind(rep(wells, each = 12), df)
names(df) <- c("well", "subPos", time)

#summarize the data
df_summary <- df %>%
  gather(key = "time", value = "RFU", -well, -subPos) %>%
  mutate(RFU = as.numeric(RFU),
         time = as.numeric(time),
         col = str_sub(well, start = 2),
         row = str_sub(well, start = 1, end = 1)) %>%
  group_by(well, time, col, row) %>%
  summarise(avgRFU = mean(RFU), maxRFU = max(RFU), minRFU = min(RFU)) %>%
  ungroup() %>%
  mutate(col = as.numeric(col))


# assign treatments
df_summary <- df_summary %>%
  mutate(Age = ifelse(col <= 6, "8 dpp", "16 dpp"),
         Treatment = ifelse(row %in% c("G", "H"), "Control", "Inoculated")) %>%
  mutate(Age = fct_relevel(Age, "8 dpp"))

# filter out H1
df_summary <- df_summary %>%
  filter(!well %in% c("H1")) %>%
  na.omit()
```

Plot fluorecense summarized over time:

```r
#plot summarised data
df_summary %>%
  ggplot(aes(x = time / 3600, y = maxRFU)) +
  stat_summary(
    data = filter(df_summary, Treatment == "Inoculated"),
    fun.data = mean_se,
    geom = "errorbar",
    aes(x = time / 3600, y = maxRFU, group = Age),
    alpha = 0.2
  ) +
  stat_summary(
    data = filter(df_summary, Treatment == "Control"),
    fun.data = mean_se,
    geom = "errorbar",
    aes(x = time / 3600, y = maxRFU, group = Age),
```

```
    alpha = 0.2
  ) +

  stat_summary(aes(color = Age, linetype = Treatment), fun.y = "mean", geom = "line", size = 1) +
  labs(x = "Hours post-inoculation", y = "Relative fluorescence") +
  cowplot::theme_cowplot(font_size = 14) +
    theme(legend.title = element_blank(),
          legend.position = c(0.1, 0.7),
          legend.direction = "vertical")
```

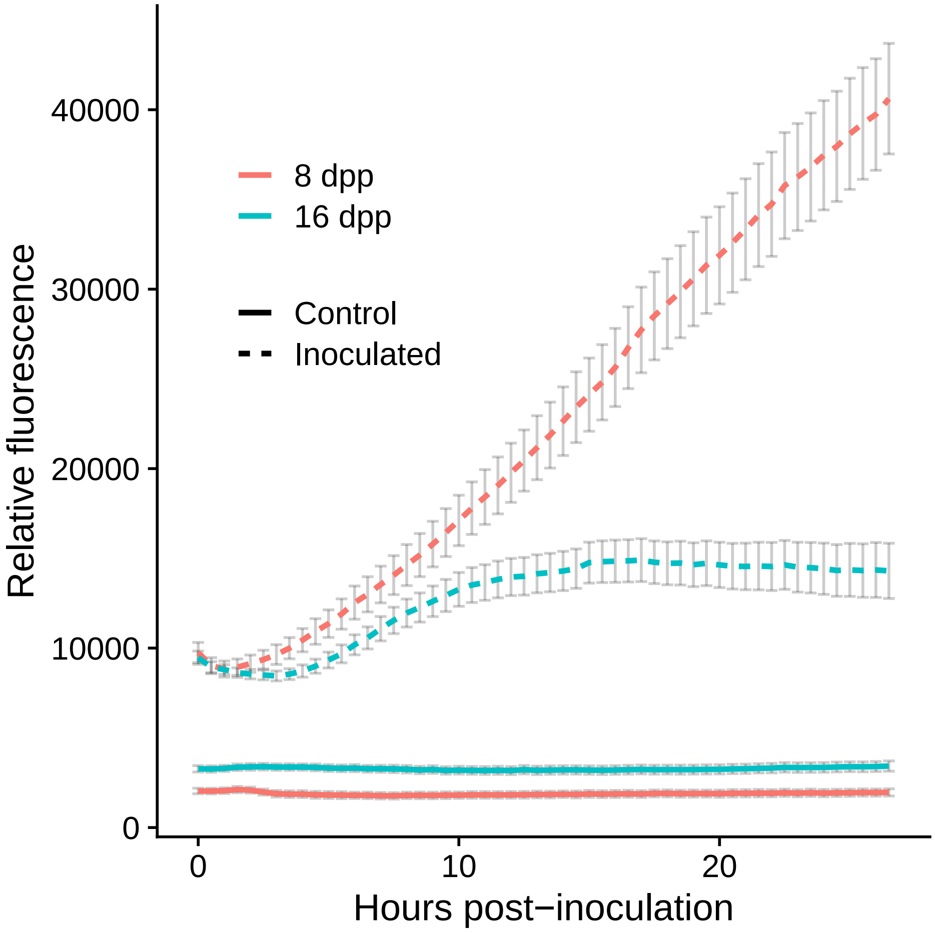

Figure 1:

```
mic <- cowplot::ggdraw() + cowplot::draw_image(image = "fluMic2.png")
poin <- cowplot::ggdraw() + cowplot::draw_image(image = "poinsett.png")

cowplot::plot_grid(drPlot, poin, mic,
                   ncol = 1,
                   labels = "AUTO",
                   rel_heights = c(3, 1.5, 1.8))
```

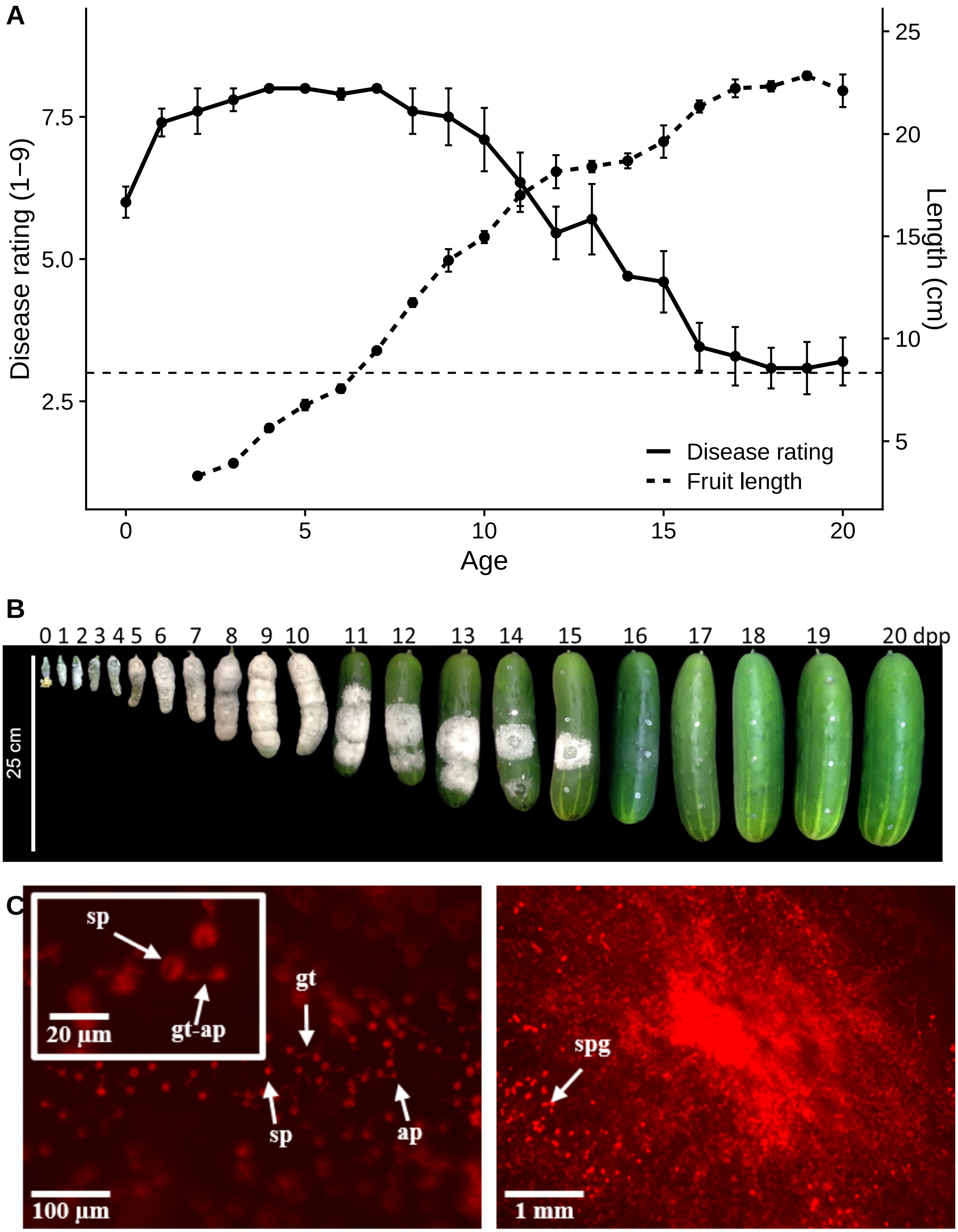


```
pdf(file = "fig1.pdf", width = 6.69, 8.85)
cowplot::plot_grid(drPlot, poin, mic,
                   ncol = 1,
```

```
                   labels = "AUTO",
                   rel_heights = c(3, 1.5, 1.8))
dev.off()
```

```
## pdf
##   2
```

Transcriptome Experiment 1

Salmon mapping

```
mkdir infectionRNAseq
mkdir genome
mkdir cl9930-v2

# Download the genome files
wget ftp://cucurbitgenomics.org/pub/cucurbit/genome/cucumber/Chinese_long/v2/*
```

Make a cdna fasta file for the cuke transcriptome

```
module load cufflinks/2.2.1

gzip -dc "~/infectionRNAseq/genome/cl9930-v2/cucumber_ChineseLong_v2.gff3.gz" > \
cucumber_ChineseLong_v2.gff3

gzip -dc "~/infectionRNAseq/genome/cl9930-v2/cucumber_ChineseLong_v2_genome.fa.gz" > \
cucumber_ChineseLong_v2_genome.fa

gffread -g cucumber_ChineseLong_v2_genome.fa \
-w cucumber_ChineseLong_v2_cdna.fa \
cucumber_ChineseLong_v2.gff3
```

```
#!/bin/bash -login
#PBS -l walltime=02:00:00
#PBS -l nodes=1:ppn=17,feature=gbe,mem=8gb
#PBS -t 1-30

#set wd
cd ${PBS_O_WORKDIR}
mkdir FastQC/
mkdir FastQC/Cleaned

# get the filename in the files.txt file at position PBS_ARRAY_INDEX
SAMPLENAME=`ls *001.fastq.gz | head -n +${PBS_ARRAYID} | tail -n 1`

module load Trimmomatic

java -jar $TRIM/trimmomatic SE \
-threads 16 \
-phred33 ${SAMPLENAME} ${SAMPLENAME%.fast*}_trimmed.fastq.gz \
ILLUMINACLIP:$ADAPTERS/TruSeq-SE.fa:2:30:10 \
LEADING:3 \
TRAILING:3 \
SLIDINGWINDOW:4:15 \
```

```
MINLEN:35

module load FastQC/0.11.3
fastqc -f fastq -noextract ${SAMPLENAME%.fast*}_trimmed.fastq.gz -o FastQC/Cleaned
```

Building a salmon index file

```
salmon index -t cucumber_ChineseLong_v2_cdna.fa -i cucumber_ChineseLong_v2_salmon_index
```

Run Salmon on the samples:

```
for fn in `ls rawReads/*trimmed.fastq.gz`;
do
nodir=${fn#rawReads/}
echo "Processing sample ${fn%_S*}"
salmon quant -i genome/cl9930-v2/cucumber_ChineseLong_v2_salmon_index -l A \
         -r ${fn} \
         -p 8 -o quants/${nodir%_S*}_quant
done
```

Differntial expression with DESeq2

load GO term functions and files.

```
source("topGO functions.R")

CukeGO <- read.csv(file = "CukeGO.csv", header = TRUE, stringsAsFactors = FALSE)

#prepare as named list for use with topGO
GOList<-setNames(nm = CukeGO$X, strsplit(CukeGO$GOTerm, "; "))
```

Differential expression analysis

Import using tximport

```
samples <- read.csv(file = "samples_exp1.csv", row.names = 1)
files <- file.path("quants", samples$directory, "quant.sf")
names(files) <- row.names(samples)

samples$timepoint <- as.factor(paste0("T", samples$timepoint))
samples$age <- relevel(samples$age, "8dpp")
samples$timepoint <- fct_relevel(samples$timepoint, "T0", "T4", "T24", "T48")

samples$condition <-
    fct_relevel(
    paste0(samples$age, "_", samples$timepoint),
    "8dpp_T0",
    "8dpp_T4",
    "8dpp_T24",
    "8dpp_T48",
    "16dpp_T0",
    "16dpp_T4",
    "16dpp_T24",
    "16dpp_T48"
    )
```

Table 1: The experimental design

age	timepoint	Reps
8dpp	T0	3
8dpp	T4	3
8dpp	T24	3
8dpp	T48	3
16dpp	T0	3
16dpp	T4	3
16dpp	T24	3
16dpp	T48	3

```
samples$name <- row.names(samples)

# make tx2gene file
tx2gene <- read.table(file = files[1], sep = "\t", header = TRUE)
tx2gene <- tx2gene[1]
tx2gene$geneid <- str_replace(tx2gene$Name, pattern = "\\..*", replacement = "")

files <- file.path("quants", samples$directory, "quant.sf")
names(files) <- row.names(samples)

txiAll <- tximport(files, type = "salmon", tx2gene = tx2gene, dropInfReps = TRUE)

ddsTxi <- DESeqDataSetFromTximport(txiAll, colData = samples, design = ~ condition)
ddsTxi_filt <- ddsTxi[rowSums(counts(ddsTxi)) >= 25, ]

dds_exp1 <- DESeq(ddsTxi_filt)
```

Reads mapping summary

```
# Import JSON files for sample read mapping results

jsons <- file.path("quants", samples$directory, "aux_info", "meta_info.json")
samples$totalReads <- sapply(jsons, FUN = function(x) {
    fromJSON(file = x)$num_processed
})

samples$Mapped <- sapply(jsons, FUN = function(x) {
    fromJSON(file = x)$num_mapped
})
```

```
expDesign <- samples %>%
    group_by(age, timepoint) %>% summarise(Reps = n())
knitr::kable(as.data.frame(expDesign), caption = "The experimental design") %>%
    kable_styling(bootstrap_options = "striped", full_width = FALSE)
```

```
samples %>%
    mutate(sampleName = rownames(.),
    Unmapped = totalReads - Mapped) %>%
    arrange(age, timepoint) %>%
    mutate(sampleName = fct_inorder(sampleName)) %>%
    gather(
```

```r
    key = "Reads",
    value = "Count",
    -directory,
    -age,
    -name,
    -timepoint,
    -condition,
    -sampleName,
    -totalReads
    ) %>%
    mutate(Percent = round(Count / totalReads * 100, digits = 1)) %>%
    group_by(Reads) %>%
    summarize(mean(Count), mean(Percent))
```

```
## # A tibble: 2 x 3
##   Reads    `mean(Count)` `mean(Percent)`
##   <chr>            <dbl>           <dbl>
## 1 Mapped       19649607.            82.3
## 2 Unmapped      4179976.            17.7
```

```r
samples %>%
    mutate(sampleName = rownames(.),
    Unmapped = totalReads - Mapped) %>%
    arrange(age, timepoint) %>%
    mutate(sampleName = fct_inorder(sampleName)) %>%
    gather(
    key = "Reads",
    value = "Count",
    -directory,
    -age,
    -name,
    -timepoint,
    -condition,
    -sampleName,
    -totalReads
    ) %>%
    ggplot(aes(
        x = sampleName,
        y = Count,
        fill = Reads,
        label = paste0(round(Count / totalReads * 100, digits = 1), "%")
        )) +
        geom_bar(stat = "identity", position = "stack") +
        geom_text(
        size = 2,
        stat = "identity",
        position = "stack",
        hjust = 0.5
        ) +
        theme(axis.text.x = element_text(angle = 90, vjust = 0.5))
```

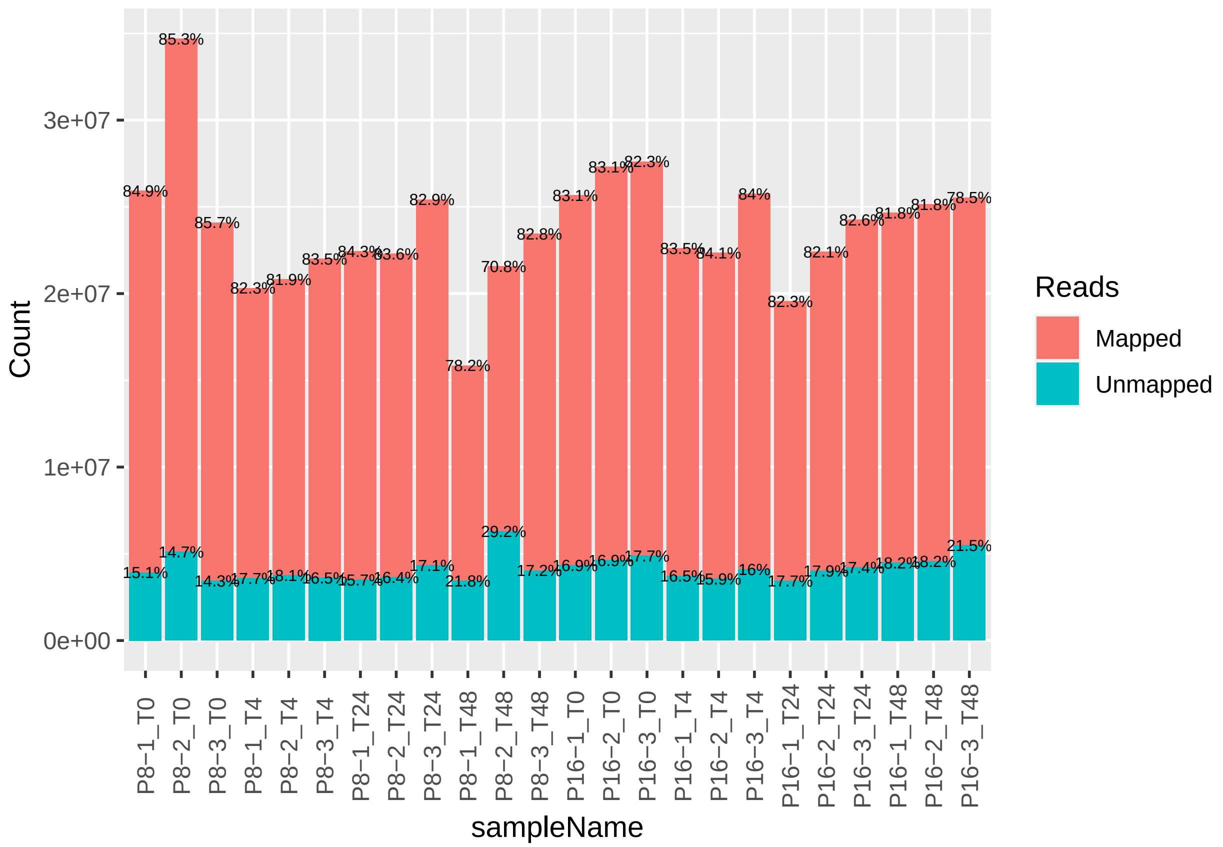

Principal component analysis

```
rld <- rlog(dds_exp1, blind = TRUE)

pcaData <- plotPCA(rld, returnData=TRUE, ntop = 500)

percentVar <- round(100 * attr(pcaData, "percentVar"))

pcaData <- pcaData %>%
    mutate(Age = dds_exp1$age,
           Timepoint = dds_exp1$timepoint)

pcaPlot1 <- ggplot(pcaData, aes(PC1, PC2, color = Timepoint, shape = Age)) +
    geom_point(size=5) +
    xlab(paste0("PC1: ",percentVar[1],"% variance")) +
    ylab(paste0("PC2: ",percentVar[2],"% variance")) +
    cowplot::theme_cowplot() +
    theme(strip.text =         element_text(
        colour = "grey10",
        size = rel(0.8),
        margin = margin(0.8 * 7, 0.8 * 7, 0.8 * 7, 0.8 * 7)
        ),
        legend.position="bottom") +
    scale_color_viridis_d(direction = -1)
   #ggrepel::geom_text_repel(aes(label = name)) # for samples names if needed

pcaPlot1
```

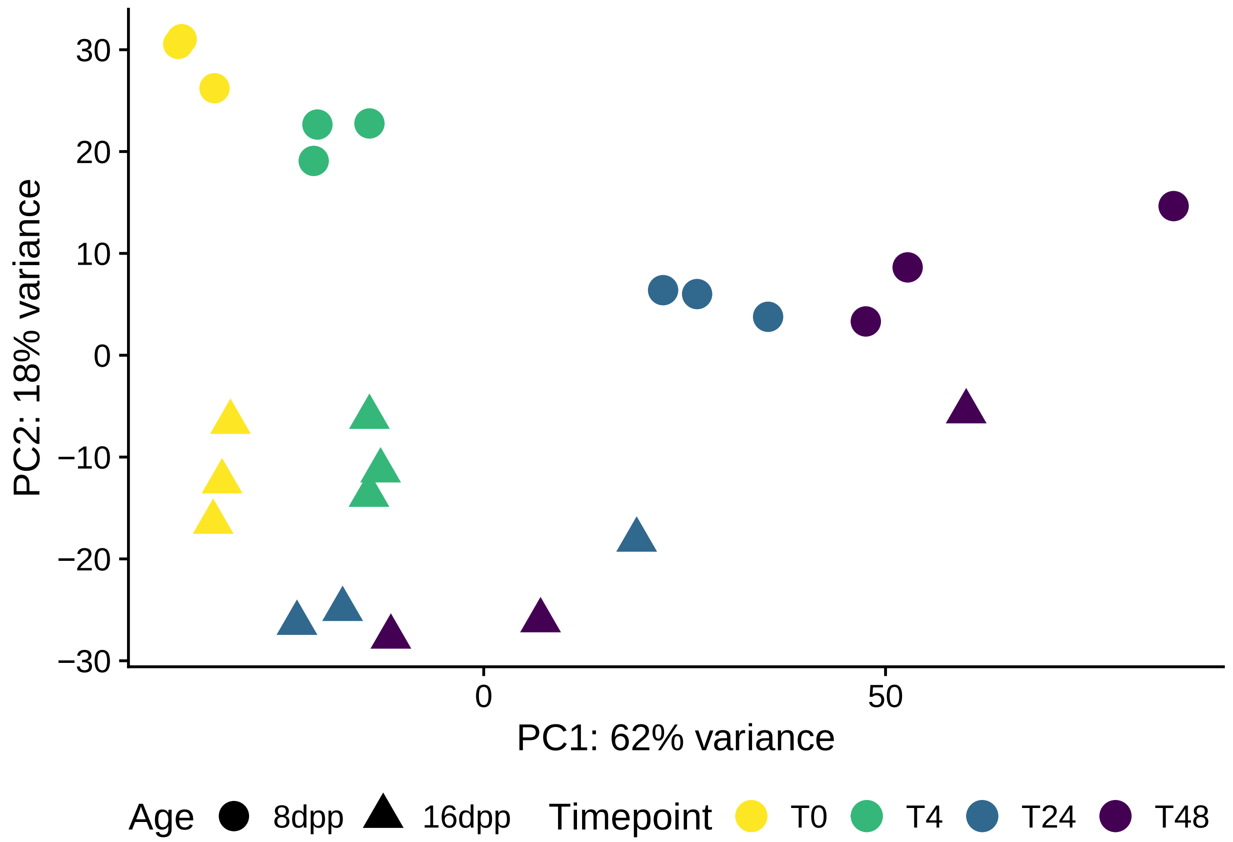

Get between sample within treatment Pearson's correlations and plot

```
rld_df <- as.data.frame(assay(rld))
cor(rld_df[, dds_exp1$age == "16dpp" & dds_exp1$timepoint == "T0"], method="pearson")
```

```
##            P16-1_T0  P16-2_T0  P16-3_T0
## P16-1_T0 1.0000000 0.9962891 0.9958321
## P16-2_T0 0.9962891 1.0000000 0.9938945
## P16-3_T0 0.9958321 0.9938945 1.0000000
```

```
cor(rld_df[, dds_exp1$age == "16dpp" & dds_exp1$timepoint == "T4"], method="pearson")
```

```
##            P16-1_T4  P16-2_T4  P16-3_T4
## P16-1_T4 1.0000000 0.9970227 0.9963668
## P16-2_T4 0.9970227 1.0000000 0.9968560
## P16-3_T4 0.9963668 0.9968560 1.0000000
```

```
cor(rld_df[, dds_exp1$age == "16dpp" & dds_exp1$timepoint == "T24"], method="pearson")
```

```
##             P16-1_T24 P16-2_T24 P16-3_T24
## P16-1_T24 1.0000000 0.9935373 0.9768389
## P16-2_T24 0.9935373 1.0000000 0.9753957
## P16-3_T24 0.9768389 0.9753957 1.0000000
```

```
cor(rld_df[, dds_exp1$age == "16dpp" & dds_exp1$timepoint == "T48"], method="pearson")
```

```
##             P16-1_T48 P16-2_T48 P16-3_T48
## P16-1_T48 1.0000000 0.9933915 0.9750264
```

```
## P16-2_T48 0.9933915 1.0000000 0.9633022
## P16-3_T48 0.9750264 0.9633022 1.0000000
```

```r
cor(rld_df[, dds_exp1$age == "8dpp" & dds_exp1$timepoint == "T0"], method="pearson")
```

```
##           P8-1_T0   P8-2_T0   P8-3_T0
## P8-1_T0 1.0000000 0.9943148 0.9922322
## P8-2_T0 0.9943148 1.0000000 0.9967608
## P8-3_T0 0.9922322 0.9967608 1.0000000
```

```r
cor(rld_df[, dds_exp1$age == "8dpp" & dds_exp1$timepoint == "T4"], method="pearson")
```

```
##           P8-1_T4   P8-2_T4   P8-3_T4
## P8-1_T4 1.0000000 0.9933884 0.9943525
## P8-2_T4 0.9933884 1.0000000 0.9964352
## P8-3_T4 0.9943525 0.9964352 1.0000000
```

```r
cor(rld_df[, dds_exp1$age == "8dpp" & dds_exp1$timepoint == "T24"], method="pearson")
```

```
##            P8-1_T24  P8-2_T24  P8-3_T24
## P8-1_T24 1.0000000 0.9947419 0.9934408
## P8-2_T24 0.9947419 1.0000000 0.9965143
## P8-3_T24 0.9934408 0.9965143 1.0000000
```

```r
cor(rld_df[, dds_exp1$age == "8dpp" & dds_exp1$timepoint == "T48"], method="pearson")
```

```
##            P8-1_T48  P8-2_T48  P8-3_T48
## P8-1_T48 1.0000000 0.9724116 0.9888864
## P8-2_T48 0.9724116 1.0000000 0.9690032
## P8-3_T48 0.9888864 0.9690032 1.0000000
```

```r
cor<-as.matrix(cor(rld_df, method="pearson"))
rownames(cor) <- colnames(cor) <- with(colData(dds_exp1), paste(age, timepoint, sep="-"))
pheatmap::pheatmap(cor)
```

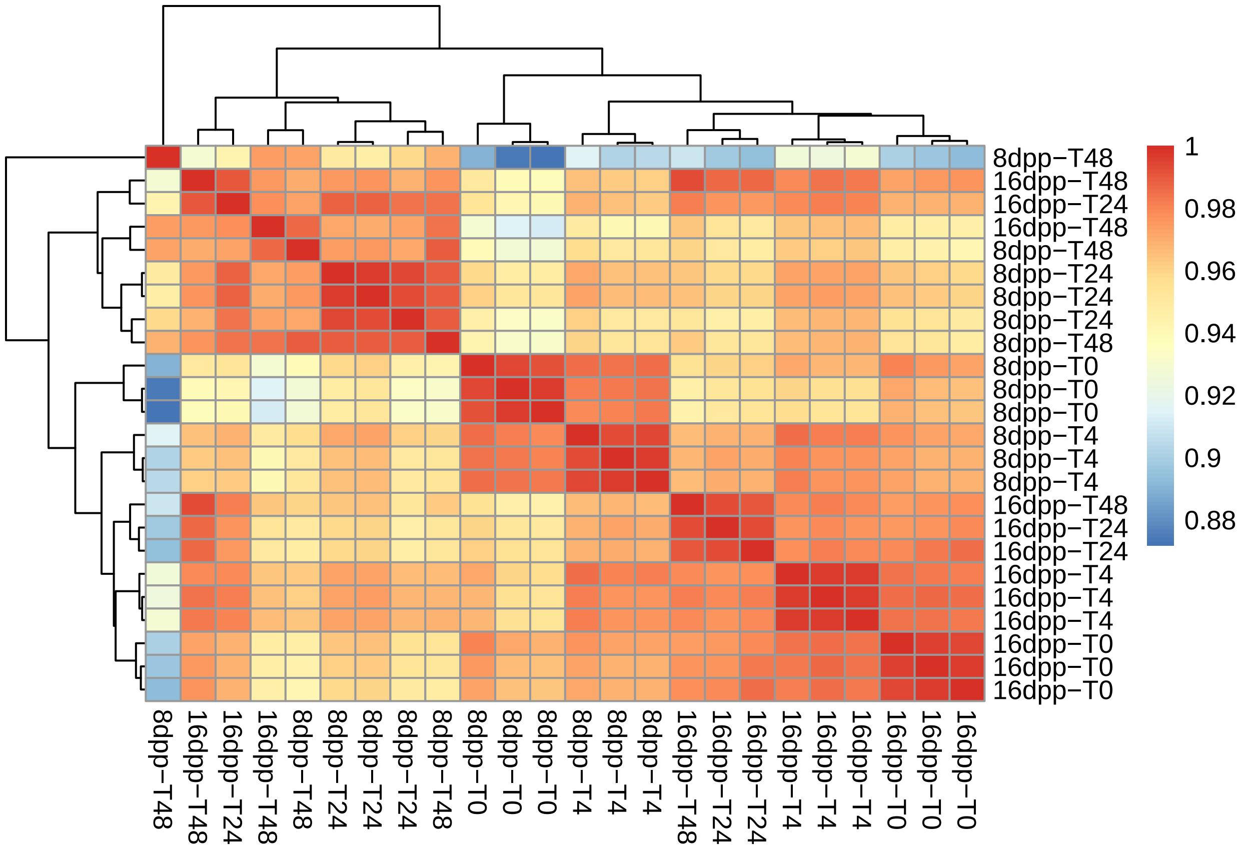

Plot Euclidean distances between samples as well

```
distsRL <- dist(t(assay(rld)))
mat <- as.matrix(distsRL)
rownames(mat) <-  colData(rld)$condition
colnames(mat) <-  colData(rld)$sampleNO

#hmcol <- colorRampPalette(brewer.pal(9, "Blues"))(255)
pheatmap(mat)
```

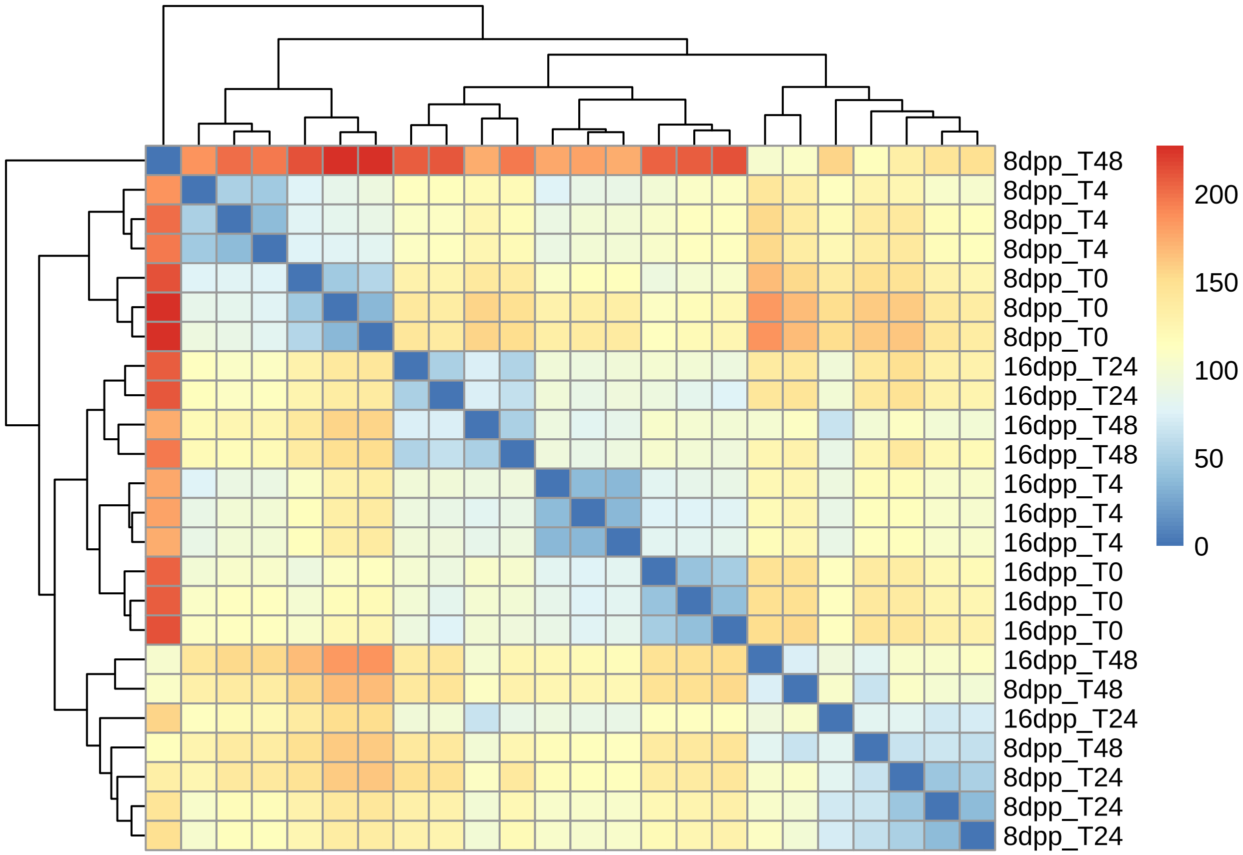

Comparison of 8 dpp and 16 dpp T0 - uninoculated fruit:

```
DE_AgeT0 <- lfcShrink(dds_exp1, "condition_16dpp_T0_vs_8dpp_T0")

summary(DE_AgeT0, 0.05)
```

```
##
## out of 19667 with nonzero total read count
## adjusted p-value < 0.05
## LFC > 0 (up)       : 3413, 17%
## LFC < 0 (down)     : 4391, 22%
## outliers [1]       : 18, 0.092%
## low counts [2]     : 0, 0%
## (mean count < 1)
## [1] see 'cooksCutoff' argument of ?results
## [2] see 'independentFiltering' argument of ?results
```

```
DE_AgeT0 %>%
    as.data.frame() %>%
    mutate(geneid = rownames(.)) %>%
    dplyr::filter(padj < 0.05 & abs(log2FoldChange) >= 1) %>%
    mutate(Direction = fct_relevel(ifelse(log2FoldChange > 0, "Up", "Down"), "Up")) %>%
    group_by(Direction) %>%
    do({tgd <- runTopGoAnalysis(DEgeneSet = .$geneid,
                                dds = dds_exp1,
                                GOdb = GOList,
                                onts = "BP",
```

```
                                nodeSize = 100)
        exportGOtable(tgd, "Fisher.weight01", n = 15) %>%
            mutate(Fisher.weight01 = as.numeric(Fisher.weight01))
            }
        )
```

```
## # A tibble: 30 x 7
## # Groups:   Direction [2]
##    Direction GO.ID   Term         Annotated Significant Expected Fisher.weight01
##    <fct>     <chr>   <chr>            <int>       <int>    <dbl>           <dbl>
##  1 Up        GO:001~ secondary m~       251          26    11.8         0.000075
##  2 Up        GO:000~ peptide met~       643          16    30.2         0.00016
##  3 Up        GO:004~ multi-organ~       136          17     6.39        0.00044
##  4 Up        GO:000~ sulfur comp~       256          24    12.0         0.00057
##  5 Up        GO:005~ cofactor me~       486          30    22.8         0.00072
##  6 Up        GO:000~ metabolic p~      9213         451   433.          0.00109
##  7 Up        GO:001~ response to~       294          28    13.8         0.00189
##  8 Up        GO:004~ regulation ~       181          17     8.5         0.00515
##  9 Up        GO:004~ root develo~       376          24    17.7         0.00518
## 10 Up        GO:005~ oxidation-r~      1249          78    58.7         0.00569
## # ... with 20 more rows
```

Extract results from dds. These are comparisons (contrasts) of consequtive timepoints within each age. Log2 Fold Changes are shrunken using the "normal" method.

```
comparsion <- 1:3
timepoints <- c("T0", "T4", "T24", "T48")


conditions <- paste0("8dpp_", timepoints)
timeContrasts8 <- bind_rows(lapply(comparsion, function(x) {
    lfc <- lfcShrink(dds_exp1, contrast = c("condition", conditions[x + 1], conditions[x]))
    as.data.frame(lfc) %>%
    mutate(
    geneid = rownames(.),
    Contrast = paste0(timepoints[x + 1]," vs ", timepoints[x])
    )
}))

conditions <- paste0("16dpp_", timepoints)
timeContrasts16 <- bind_rows(lapply(comparsion, function(x) {
    lfc <- lfcShrink(dds_exp1, contrast = c("condition", conditions[x + 1], conditions[x]))
    as.data.frame(lfc) %>%
    mutate(
    geneid = rownames(.),
    Contrast = paste0(timepoints[x + 1]," vs ", timepoints[x])
    )
}))

timeContrasts <- bind_rows("8dpp" = timeContrasts8, "16dpp" = timeContrasts16, .id = "Age") %>%
    mutate(Contrast = fct_inorder(Contrast),
           Age = fct_relevel(Age, "8dpp")
           )
```

Plot a summary of the differentially expressed genes in each contrast.

```r
sumPlot1 <- timeContrasts %>%
    filter(padj < 0.05 & abs(log2FoldChange) >= 1) %>%
    mutate(Direction = fct_relevel(ifelse(log2FoldChange > 0, "Up", "Down"), "Up")) %>%
    group_by(Contrast, Age, Direction) %>%
    ggplot(aes(x = Direction, fill = Age)) +
    geom_bar(position = "dodge") +
    geom_text(stat = 'count', aes(label = ..count..), vjust = -0.5, position = position_dodge(0.90)) +
    # facet_wrap(~ Contrast) +
    scale_y_continuous(expand = expand_scale(mult = c(0.05, 0.1)), name = "Number of DEGs") +
    cowplot::theme_cowplot(font_size = 14) +
    theme(strip.text = element_text(
        colour = "grey10",
        size = rel(0.8),
        margin = margin(0.5 * 7, 0.5 * 7, 0.5 * 7, 0.5 * 7)
        ),
        legend.position = c(0.825, 0.825),
        axis.line=element_line()) +
    lemon::facet_rep_wrap(~ Contrast) +
     cowplot::panel_border()

sumPlot1
```

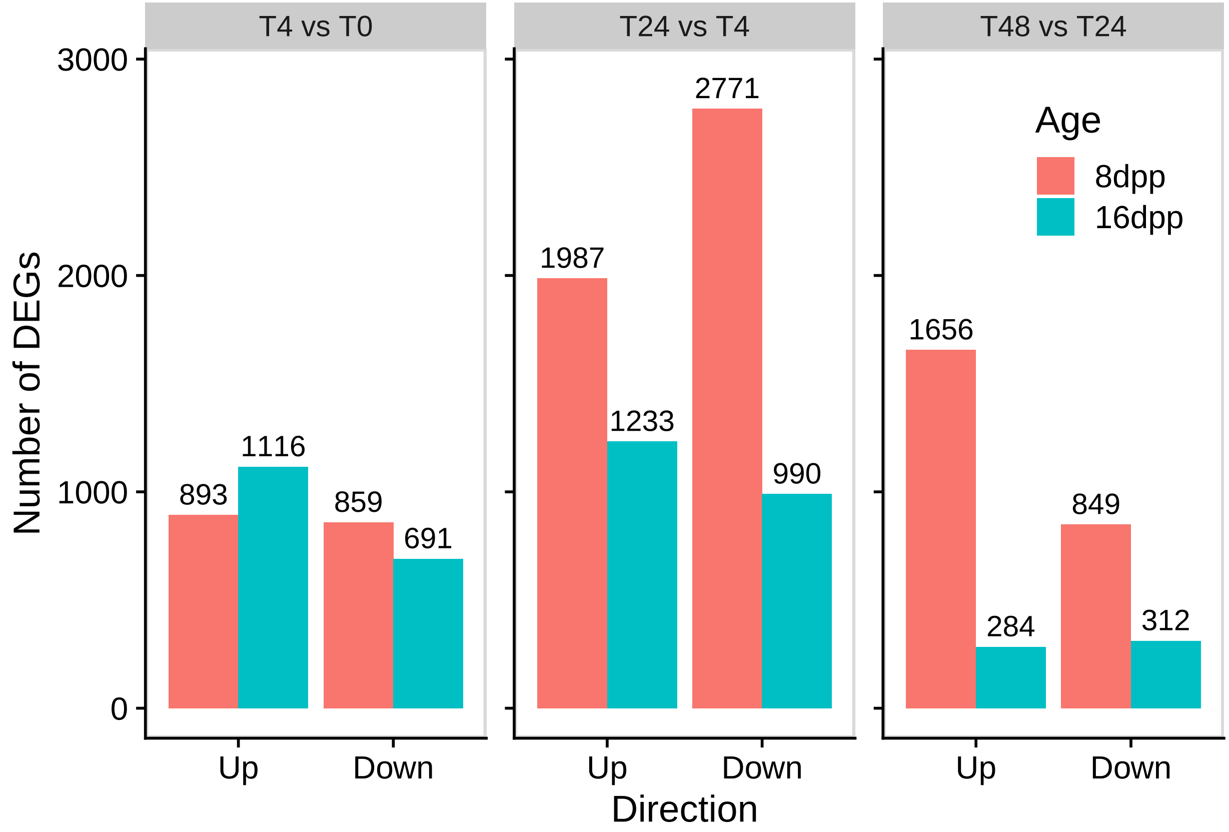

Extract Gene Ontology terms for each contrast:

```r
allGOres <- timeContrasts %>%
    filter(padj < 0.05 & abs(log2FoldChange) >= 1) %>%
    mutate(Direction = fct_relevel(ifelse(log2FoldChange > 0, "Up", "Down"), "Up")) %>%
```

```
    group_by(Contrast, Age, Direction) %>%
    # filter(Age == "16dpp", Contrast == "T4 vs T0", Direction == "Up") %>%
    do({tgd <- runTopGoAnalysis(DEgeneSet = .$geneid,
                                dds = dds_exp1,
                                GOdb = GOList,
                                onts = "BP",
                                nodeSize = 100)
       exportGOtable(tgd, "Fisher.weight01", n = 500) %>%
           mutate(Fisher.weight01 = as.numeric(Fisher.weight01))
           }
       )
```

Tables of results

```
for (i in unique(allGOres$Contrast)) {
print(
kable(allGOres %>%
          filter(Contrast == i ) %>%
          top_n(n = 10, wt = dplyr::desc(Fisher.weight01)), digits = 20) %>%
    kable_styling(bootstrap_options = "striped", full_width = FALSE, latex_options="scale_down") %>%
    kableExtra::kable_styling(latex_options = c("HOLD_position"))
)
}
```

Contrast	Age	Direction	GO.ID	Term	Annotated	Significant	Expected	Fisher.weight01
T4 vs T0	8dpp	Up	GO:0006979	response to oxidative stress	380	66	17.47	2.10e-19
T4 vs T0	8dpp	Up	GO:0009611	response to wounding	259	43	11.91	1.90e-13
T4 vs T0	8dpp	Up	GO:0006952	defense response	1018	118	46.81	2.50e-13
T4 vs T0	8dpp	Up	GO:0009699	phenylpropanoid biosynthetic process	133	29	6.12	1.70e-12
T4 vs T0	8dpp	Up	GO:0080167	response to karrikin	141	29	6.48	7.80e-12
T4 vs T0	8dpp	Up	GO:0042737	drug catabolic process	151	30	6.94	8.60e-12
T4 vs T0	8dpp	Up	GO:1901565	organonitrogen compound catabolic proces...	489	41	22.48	2.50e-11
T4 vs T0	8dpp	Up	GO:0050832	defense response to fungus	239	36	10.99	3.10e-10
T4 vs T0	8dpp	Up	GO:0010200	response to chitin	134	26	6.16	3.70e-10
T4 vs T0	8dpp	Up	GO:0019439	aromatic compound catabolic process	215	23	9.89	1.20e-07
T4 vs T0	8dpp	Down	GO:0071555	cell wall organization	323	48	14.75	1.40e-09
T4 vs T0	8dpp	Down	GO:0007017	microtubule-based process	137	20	6.26	4.00e-06
T4 vs T0	8dpp	Down	GO:0006073	cellular glucan metabolic process	171	21	7.81	3.50e-05
T4 vs T0	8dpp	Down	GO:0016114	terpenoid biosynthetic process	110	16	5.02	3.80e-05
T4 vs T0	8dpp	Down	GO:0009664	plant-type cell wall organization	101	15	4.61	5.20e-05
T4 vs T0	8dpp	Down	GO:0000272	polysaccharide catabolic process	130	17	5.94	8.80e-05
T4 vs T0	8dpp	Down	GO:0051707	response to other organism	839	40	38.31	3.80e-04
T4 vs T0	8dpp	Down	GO:0009651	response to salt stress	524	40	23.93	1.04e-03
T4 vs T0	8dpp	Down	GO:0009826	unidimensional cell growth	234	23	10.69	3.11e-03
T4 vs T0	8dpp	Down	GO:0007169	transmembrane receptor protein tyrosine ...	135	14	6.16	3.49e-03
T4 vs T0	16dpp	Up	GO:0009611	response to wounding	259	61	15.32	0.00e+00
T4 vs T0	16dpp	Up	GO:0006979	response to oxidative stress	380	67	22.47	7.80e-17
T4 vs T0	16dpp	Up	GO:0080167	response to karrikin	141	37	8.34	6.50e-15
T4 vs T0	16dpp	Up	GO:0042737	drug catabolic process	151	38	8.93	1.20e-14
T4 vs T0	16dpp	Up	GO:0010200	response to chitin	134	35	7.92	4.10e-14
T4 vs T0	16dpp	Up	GO:0009699	phenylpropanoid biosynthetic process	133	33	7.86	1.10e-12
T4 vs T0	16dpp	Up	GO:1901565	organonitrogen compound catabolic proces...	489	53	28.92	7.60e-12
T4 vs T0	16dpp	Up	GO:1901605	alpha-amino acid metabolic process	276	40	16.32	1.40e-10
T4 vs T0	16dpp	Up	GO:0046395	carboxylic acid catabolic process	131	29	7.75	4.70e-10
T4 vs T0	16dpp	Up	GO:0050832	defense response to fungus	239	40	14.13	2.10e-09
T4 vs T0	16dpp	Down	GO:0009734	auxin-activated signaling pathway	150	18	5.53	1.10e-05
T4 vs T0	16dpp	Down	GO:0006355	regulation of transcription, DNA-templat...	1745	97	64.38	3.90e-05
T4 vs T0	16dpp	Down	GO:0071555	cell wall organization	323	26	11.92	7.40e-05
T4 vs T0	16dpp	Down	GO:0009699	phenylpropanoid biosynthetic process	133	15	4.91	1.20e-04
T4 vs T0	16dpp	Down	GO:0009059	macromolecule biosynthetic process	2827	126	104.30	1.18e-03
T4 vs T0	16dpp	Down	GO:0008152	metabolic process	9213	350	339.92	1.39e-03
T4 vs T0	16dpp	Down	GO:0007169	transmembrane receptor protein tyrosine ...	135	13	4.98	1.47e-03
T4 vs T0	16dpp	Down	GO:0042445	hormone metabolic process	261	14	9.63	1.92e-03
T4 vs T0	16dpp	Down	GO:0007275	multicellular organism development	2197	101	81.06	2.11e-03
T4 vs T0	16dpp	Down	GO:0080167	response to karrikin	141	13	5.20	2.17e-03

Contrast	Age	Direction	GO.ID	Term	Annotated	Significant	Expected	Fisher.weight01
T24 vs T4	8dpp	Up	GO:0006952	defense response	1018	172	103.56	2.80e-11
T24 vs T4	8dpp	Up	GO:0006260	DNA replication	119	36	12.11	1.10e-09
T24 vs T4	8dpp	Up	GO:0006412	translation	564	94	57.38	1.30e-09
T24 vs T4	8dpp	Up	GO:0046686	response to cadmium ion	335	66	34.08	9.90e-08
T24 vs T4	8dpp	Up	GO:0009751	response to salicylic acid	145	33	14.75	7.00e-06
T24 vs T4	8dpp	Up	GO:0006468	protein phosphorylation	935	135	95.12	7.70e-05
T24 vs T4	8dpp	Up	GO:0009651	response to salt stress	524	81	53.31	8.10e-05
T24 vs T4	8dpp	Up	GO:0042254	ribosome biogenesis	273	47	27.77	1.60e-04
T24 vs T4	8dpp	Up	GO:0097659	nucleic acid-templated transcription	1867	178	189.93	2.00e-04
T24 vs T4	8dpp	Up	GO:0042742	defense response to bacterium	306	51	31.13	2.80e-04
T24 vs T4	8dpp	Down	GO:0055114	oxidation-reduction process	1249	267	184.64	4.40e-11
T24 vs T4	8dpp	Down	GO:0015979	photosynthesis	201	114	29.71	1.10e-10
T24 vs T4	8dpp	Down	GO:0009416	response to light stimulus	640	140	94.61	5.40e-06
T24 vs T4	8dpp	Down	GO:0006073	cellular glucan metabolic process	171	46	25.28	2.60e-05
T24 vs T4	8dpp	Down	GO:0005975	carbohydrate metabolic process	804	158	118.86	3.50e-05
T24 vs T4	8dpp	Down	GO:0009735	response to cytokinin	238	58	35.18	6.00e-05
T24 vs T4	8dpp	Down	GO:0071702	organic substance transport	923	119	136.45	1.20e-04
T24 vs T4	8dpp	Down	GO:0042446	hormone biosynthetic process	185	46	27.35	2.00e-04
T24 vs T4	8dpp	Down	GO:0042546	cell wall biogenesis	143	29	21.14	3.50e-04
T24 vs T4	8dpp	Down	GO:0007169	transmembrane receptor protein tyrosine ...	135	35	19.96	4.90e-04
T24 vs T4	16dpp	Up	GO:0019684	photosynthesis, light reaction	115	23	7.30	7.50e-07
T24 vs T4	16dpp	Up	GO:0055114	oxidation-reduction process	1249	119	79.33	3.90e-06
T24 vs T4	16dpp	Up	GO:0097659	nucleic acid-templated transcription	1867	129	118.59	1.90e-05
T24 vs T4	16dpp	Up	GO:0006325	chromatin organization	230	26	14.61	1.30e-04
T24 vs T4	16dpp	Up	GO:0009658	chloroplast organization	148	22	9.40	1.60e-04
T24 vs T4	16dpp	Up	GO:0051649	establishment of localization in cell	439	17	27.88	4.50e-04
T24 vs T4	16dpp	Up	GO:0009416	response to light stimulus	640	72	40.65	7.40e-04
T24 vs T4	16dpp	Up	GO:0080167	response to karrikin	141	19	8.96	1.50e-03
T24 vs T4	16dpp	Up	GO:1903046	meiotic cell cycle process	102	15	6.48	1.91e-03
T24 vs T4	16dpp	Up	GO:0016311	dephosphorylation	149	14	9.46	1.92e-03
T24 vs T4	16dpp	Down	GO:0009611	response to wounding	259	37	13.81	4.10e-08
T24 vs T4	16dpp	Down	GO:0009873	ethylene-activated signaling pathway	158	27	8.42	7.10e-08
T24 vs T4	16dpp	Down	GO:0033036	macromolecule localization	710	31	37.85	1.30e-06
T24 vs T4	16dpp	Down	GO:0006979	response to oxidative stress	380	37	20.26	1.40e-06
T24 vs T4	16dpp	Down	GO:0050832	defense response to fungus	239	31	12.74	4.10e-06
T24 vs T4	16dpp	Down	GO:0071702	organic substance transport	923	56	49.20	9.30e-06
T24 vs T4	16dpp	Down	GO:0009753	response to jasmonic acid	205	26	10.93	3.60e-05
T24 vs T4	16dpp	Down	GO:0010200	response to chitin	134	18	7.14	2.70e-04
T24 vs T4	16dpp	Down	GO:0042737	drug catabolic process	151	19	8.05	4.30e-04
T24 vs T4	16dpp	Down	GO:0016999	antibiotic metabolic process	128	17	6.82	4.50e-04

Contrast	Age	Direction	GO.ID	Term	Annotated	Significant	Expected	Fisher.weight01
T48 vs T24	8dpp	Up	GO:0009611	response to wounding	259	63	21.87	7.400e-15
T48 vs T24	8dpp	Up	GO:0046395	carboxylic acid catabolic process	131	33	11.06	7.700e-09
T48 vs T24	8dpp	Up	GO:1901565	organonitrogen compound catabolic proces...	489	65	41.29	1.000e-08
T48 vs T24	8dpp	Up	GO:0009753	response to jasmonic acid	205	40	17.31	4.300e-07
T48 vs T24	8dpp	Up	GO:0051707	response to other organism	839	119	70.85	5.400e-07
T48 vs T24	8dpp	Up	GO:0010200	response to chitin	134	30	11.31	5.900e-07
T48 vs T24	8dpp	Up	GO:0009414	response to water deprivation	318	51	26.85	6.100e-06
T48 vs T24	8dpp	Up	GO:0031669	cellular response to nutrient levels	113	25	9.54	6.300e-06
T48 vs T24	8dpp	Up	GO:0042594	response to starvation	108	24	9.12	8.900e-06
T48 vs T24	8dpp	Up	GO:1901605	alpha-amino acid metabolic process	276	40	23.31	1.300e-05
T48 vs T24	8dpp	Down	GO:0019684	photosynthesis, light reaction	115	22	5.32	1.200e-08
T48 vs T24	8dpp	Down	GO:0009416	response to light stimulus	640	59	29.59	2.700e-07
T48 vs T24	8dpp	Down	GO:0006633	fatty acid biosynthetic process	167	24	7.72	7.500e-07
T48 vs T24	8dpp	Down	GO:0015979	photosynthesis	201	36	9.29	2.900e-05
T48 vs T24	8dpp	Down	GO:0090567	reproductive shoot system development	408	24	18.86	2.000e-04
T48 vs T24	8dpp	Down	GO:0071555	cell wall organization	323	30	14.93	6.000e-04
T48 vs T24	8dpp	Down	GO:0007275	multicellular organism development	2197	113	101.57	6.200e-04
T48 vs T24	8dpp	Down	GO:0009607	response to biotic stimulus	856	48	39.57	7.900e-04
T48 vs T24	8dpp	Down	GO:0000272	polysaccharide catabolic process	130	15	6.01	9.800e-04
T48 vs T24	8dpp	Down	GO:0033036	macromolecule localization	710	22	32.82	1.300e-03
T48 vs T24	16dpp	Up	GO:0006950	response to stress	2648	72	39.15	1.400e-08
T48 vs T24	16dpp	Up	GO:0009628	response to abiotic stimulus	1764	35	26.08	2.900e-07
T48 vs T24	16dpp	Up	GO:0009664	plant-type cell wall organization	101	9	1.49	1.900e-05
T48 vs T24	16dpp	Up	GO:0055114	oxidation-reduction process	1249	37	18.46	3.700e-05
T48 vs T24	16dpp	Up	GO:0070887	cellular response to chemical stimulus	889	27	13.14	3.800e-05
T48 vs T24	16dpp	Up	GO:0006633	fatty acid biosynthetic process	167	10	2.47	1.900e-04
T48 vs T24	16dpp	Up	GO:0050790	regulation of catalytic activity	180	10	2.66	3.500e-04
T48 vs T24	16dpp	Up	GO:0002376	immune system process	327	11	4.83	6.900e-04
T48 vs T24	16dpp	Up	GO:0009753	response to jasmonic acid	205	10	3.03	9.700e-04
T48 vs T24	16dpp	Up	GO:0019220	regulation of phosphate metabolic proces...	109	7	1.61	1.170e-03
T48 vs T24	16dpp	Down	GO:0055114	oxidation-reduction process	1249	40	21.75	1.300e-04
T48 vs T24	16dpp	Down	GO:0000160	phosphorelay signal transduction system	192	10	3.34	2.700e-04
T48 vs T24	16dpp	Down	GO:0042446	hormone biosynthetic process	185	10	3.22	1.530e-03
T48 vs T24	16dpp	Down	GO:0008610	lipid biosynthetic process	473	19	8.24	3.870e-03
T48 vs T24	16dpp	Down	GO:0009408	response to heat	178	9	3.10	4.040e-03
T48 vs T24	16dpp	Down	GO:0006325	chromatin organization	230	8	4.01	7.350e-03
T48 vs T24	16dpp	Down	GO:0050896	response to stimulus	4815	114	83.85	8.160e-03
T48 vs T24	16dpp	Down	GO:0048878	chemical homeostasis	318	8	5.54	8.530e-03
T48 vs T24	16dpp	Down	GO:0007169	transmembrane receptor protein tyrosine ...	135	7	2.35	9.420e-03
T48 vs T24	16dpp	Down	GO:0009414	response to water deprivation	318	12	5.54	1.008e-02

Extract the top terms in T4 vs T0 regardless of age:

```
allGOres %>%
    filter(Contrast == "T4 vs T0") %>%
    filter(Direction == "Up") %>%
    group_by(Age) %>%
    top_n(n = 10, wt = dplyr::desc(Fisher.weight01)) %>%
    pull(Term) %>% unique()
```

```
##  [1] "response to oxidative stress"
##  [2] "response to wounding"
##  [3] "defense response"
##  [4] "phenylpropanoid biosynthetic process"
##  [5] "response to karrikin"
##  [6] "drug catabolic process"
##  [7] "organonitrogen compound catabolic proces..."
##  [8] "defense response to fungus"
##  [9] "response to chitin"
## [10] "aromatic compound catabolic process"
## [11] "alpha-amino acid metabolic process"
## [12] "carboxylic acid catabolic process"
```

Plot heatmaps of GO terms over consequtive contrasts (Figure 3):

```
library(ggdendro)
source("myheatmap.R")

legend <- cowplot::get_legend(allGOres %>%
                         ungroup() %>%
                         filter(Fisher.weight01 < 0.01) %>%
                         #separate(col = Contrast, into = c("Age", "Contrast", "Direction"), sep = "_",
                         mutate(Term = factor(Term, unique(Term)),
                                Contrast = paste(Age, Contrast, sep = "_")) %>%
                         #mutate(Term = factor(Term,) %>%
                         #mutate(Age = fct_relevel(Age, "P8")) %>%
                         ggplot() +
                         geom_tile(aes(
                             x = Age,
                             y = factor(str_trunc(as.character(Term), 40, "right")),
                             fill = -(Fisher.weight01)
                         )) +
                         geom_vline(xintercept = 1.5, color = "grey92") +
                         scale_y_discrete(position = "right") +
                         scale_fill_gradient(name = "-log10(p-value)",
                                             low = "#ffeda0",
                                             high = "#f03b20",
                                             limits = c(1.30103, 25)) +
                         theme(legend.position = "bottom",
                               text = element_text(size = 10),
                               plot.margin = unit(c(0, 0, 0, 0), "cm"),
                               legend.margin = unit(c(0, 0, 0, 0), "cm")
                               )
)

### Plot all

p1 <- myheatmap2(allGOres ,"Up", trim = 35, pval = 0.01)
p2 <- myheatmap2(allGOres, "Down", trim = 35, pval = 0.01)

pall <- cowplot::ggdraw(cowplot::plot_grid(
    cowplot::plot_grid(
        p1,
        p2,
        ncol = 2,
        align = 'h',
        rel_widths = c(1, 1),
        labels = "AUTO"
    ),
    cowplot::plot_grid(
        NULL,
        legend,
        NULL,
        ncol = 3,
        rel_widths = c(0.8, 0.2, 1)
    ),
    nrow = 2,
```

```
    rel_heights = c(1, 0.05)
))

pall
```

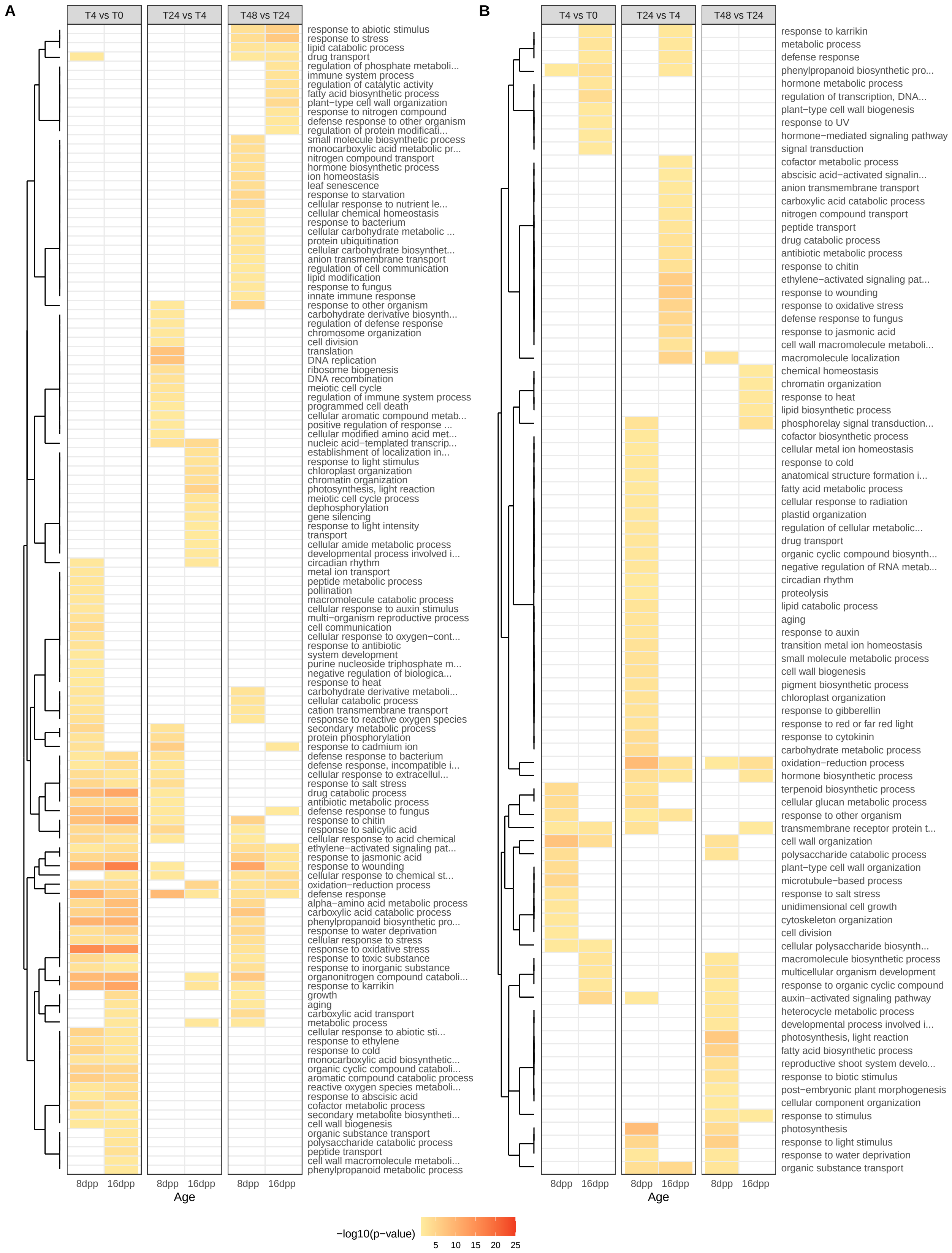
A
T4 vs T0
T24 vs T4
T48 vs T24
response to abiotic stimulus
response to stress
lipid catabolic process
drug transport
regulation of phosphate metaboli...
immune system process
regulation of catalytic activity
fatty acid biosynthetic process
plant−type cell wall organization
response to nitrogen compound
defense response to other organism
regulation of protein modificati...
small molecule biosynthetic process
monocarboxylic acid metabolic pr...
nitrogen compound transport
hormone biosynthetic process
ion homeostasis
leaf senescence
response to starvation
cellular response to nutrient le...
cellular chemical homeostasis
response to bacterium
cellular carbohydrate metabolic ...
protein ubiquitination
cellular carbohydrate biosynthet...
anion transmembrane transport
regulation of cell communication
lipid modification
response to fungus
innate immune response
response to other organism
carbohydrate derivative biosynth...
regulation of defense response
chromosome organization
cell division
translation
DNA replication
ribosome biogenesis
DNA recombination
meiotic cell cycle
regulation of immune system process
programmed cell death
cellular aromatic compound metab...
positive regulation of response ...
cellular modified amino acid met...
nucleic acid−templated transcrip...
establishment of localization in...
response to light stimulus
chloroplast organization
chromatin organization
photosynthesis, light reaction
meiotic cell cycle process
dephosphorylation
gene silencing
response to light intensity
transport
cellular amide metabolic process
developmental process involved i...
circadian rhythm
metal ion transport
peptide metabolic process
pollination
macromolecule catabolic process
cellular response to auxin stimulus
multi−organism reproductive process
cell communication
cellular response to oxygen−cont...
response to antibiotic
system development
purine nucleoside triphosphate m...
negative regulation of biologica...
response to heat
carbohydrate derivative metaboli...
cellular catabolic process
cation transmembrane transport
response to reactive oxygen species
secondary metabolic process
protein phosphorylation
response to cadmium ion
defense response to bacterium
defense response, incompatible i...
cellular response to extracellul...
response to salt stress
drug catabolic process
antibiotic metabolic process
defense response to fungus
response to chitin
response to salicylic acid
cellular response to acid chemical
ethylene−activated signaling pat...
response to jasmonic acid
response to wounding
cellular response to chemical st...
oxidation−reduction process
defense response
alpha−amino acid metabolic process
carboxylic acid catabolic process
phenylpropanoid biosynthetic pro...
response to water deprivation
cellular response to stress
response to oxidative stress
response to toxic substance
response to inorganic substance
organonitrogen compound cataboli...
response to karrikin
growth
aging
carboxylic acid transport
metabolic process
cellular response to abiotic sti...
response to ethylene
response to cold
monocarboxylic acid biosynthetic...
organic cyclic compound cataboli...
aromatic compound catabolic process
reactive oxygen species metaboli...
response to abscisic acid
cofactor metabolic process
secondary metabolite biosyntheti...
cell wall biogenesis
organic substance transport
polysaccharide catabolic process
peptide transport
cell wall macromolecule metaboli...
phenylpropanoid metabolic process
8dpp 16dpp 8dpp 16dpp 8dpp 16dpp
Age
B
T4 vs T0
T24 vs T4
T48 vs T24
response to karrikin
metabolic process
defense response
phenylpropanoid biosynthetic pro...
hormone metabolic process
regulation of transcription, DNA...
plant−type cell wall biogenesis
response to UV
hormone−mediated signaling pathway
signal transduction
cofactor metabolic process
abscisic acid−activated signalin...
anion transmembrane transport
carboxylic acid catabolic process
nitrogen compound transport
peptide transport
drug catabolic process
antibiotic metabolic process
response to chitin
ethylene−activated signaling pat...
response to wounding
response to oxidative stress
defense response to fungus
response to jasmonic acid
cell wall macromolecule metaboli...
macromolecule localization
chemical homeostasis
chromatin organization
response to heat
lipid biosynthetic process
phosphorelay signal transduction...
cofactor biosynthetic process
cellular metal ion homeostasis
response to cold
anatomical structure formation i...
fatty acid metabolic process
cellular response to radiation
plastid organization
regulation of cellular metabolic...
drug transport
organic cyclic compound biosynth...
negative regulation of RNA metab...
circadian rhythm
proteolysis
lipid catabolic process
aging
response to auxin
transition metal ion homeostasis
small molecule metabolic process
cell wall biogenesis
pigment biosynthetic process
chloroplast organization
response to gibberellin
response to red or far red light
response to cytokinin
carbohydrate metabolic process
oxidation−reduction process
hormone biosynthetic process
terpenoid biosynthetic process
cellular glucan metabolic process
response to other organism
transmembrane receptor protein t...
cell wall organization
polysaccharide catabolic process
plant−type cell wall organization
microtubule−based process
response to salt stress
unidimensional cell growth
cytoskeleton organization
cell division
cellular polysaccharide biosynth...
macromolecule biosynthetic process
multicellular organism development
response to organic cyclic compound
auxin−activated signaling pathway
heterocycle metabolic process
developmental process involved i...
photosynthesis, light reaction
fatty acid biosynthetic process
reproductive shoot system develo...
response to biotic stimulus
post−embryonic plant morphogenesis
cellular component organization
response to stimulus
photosynthesis
response to light stimulus
response to water deprivation
organic substance transport
8dpp 16dpp 8dpp 16dpp 8dpp 16dpp
Age
−log10(p−value)
5 10 15 20 25

```
pdf("fig3.pdf", width = 12, height = 16)
pall
dev.off()
```

```
## pdf
##   2
```

Venn diagrams of differntially expressed genes at each consecutive contrast:

```
# Imports required functions. Originally from http://faculty.ucr.edu/~tgirke/Documents/R_BioCond/My_R_S
source("overLapper.R.txt")

# ellipse plotting functions:
source(file = "plotellipse.R")
```

```
venninput <- timeContrasts %>%
    filter(padj < 0.05 & abs(log2FoldChange) >= 1) %>%
    filter(Contrast == "T4 vs T0") %>%
    mutate(Direction = fct_relevel(ifelse(log2FoldChange > 0, "Up", "Down"), "Up")) %>%
    group_by(Contrast, Age, Direction) %>%
    do(data = (.$geneid)) %>%
    arrange(Direction, desc(Age)) %>%
    with(set_names(data, paste(Contrast, Age, Direction)))


OLlist <-
    overLapper(setlist = venninput,
               sep = "_",
               type = "vennsets")

counts <- list(sapply(OLlist$Venn_List, length))

venndf <- data.frame(
    count = as.vector(lengths(OLlist$Venn_List)),
    y = c(4, 4, 3, 1, 4, 3, 1, 3, 1, 2, 3, 1, 2, 2, 2),
    x = c(1, 3, 4, 4, 2, 1, 1, 3, 3, 4, 2, 2, 1, 3, 2)
)

#create venn ellipses
ellipse1 <-
    as.data.frame(
        plotellipse(
            center = c(2.6, 1.4),
            radius = c(1.15, 2.25),
            rotate = 90,
            segments = 360,
            xlab = "",
            ylab = "",
            col = lines[1],
            axes = FALSE,
            main = mymain,
            sub = mysub,
            lwd = mylwd
        )
    )
```

```r
ellipse2 <-
    as.data.frame(
        plotellipse(
            center = c(2.5, 2.5),
            radius = c(1.15, 2.25),
            rotate = 90,
            segments = 360,
            xlab = "",
            ylab = "",
            col = lines[1],
            axes = FALSE,
            main = mymain,
            sub = mysub,
            lwd = mylwd
        )
    )
ellipse3 <-
    as.data.frame(
        plotellipse(
            center = c(2.5, 2.5),
            radius = c(1.15, 2.25),
            rotate = 0,
            segments = 360,
            xlab = "",
            ylab = "",
            col = lines[1],
            axes = FALSE,
            main = mymain,
            sub = mysub,
            lwd = mylwd
        )
    )
ellipse4 <-
    as.data.frame(
        plotellipse(
            center = c(1.4, 2.6),
            radius = c(1.15, 2.25),
            rotate = 0,
            segments = 360,
            xlab = "",
            ylab = "",
            col = lines[1],
            axes = FALSE,
            main = mymain,
            sub = mysub,
            lwd = mylwd
        )
    )

library("sp")
poly1 <- Polygon(ellipse1)
poly2 <- Polygon(ellipse2)
poly3 <- Polygon(ellipse3)
```

```r
poly4 <- Polygon(ellipse4)
# create SpatialPolygons objects
p1 <- SpatialPolygons(list(Polygons(list(poly1), "p1")))
p2 <- SpatialPolygons(list(Polygons(list(poly2), "p2")))
p3 <- SpatialPolygons(list(Polygons(list(poly3), "p3")))
p4 <- SpatialPolygons(list(Polygons(list(poly4), "p4")))

# highlight intersections
library(rgeos)
int1 <- gDifference(gDifference(gIntersection(p1, p4), p2), p3)
highlight1<-as.data.frame(slot(int1@polygons[[1]]@Polygons[[1]], "coords"))
highlight1$group <- "1"

int2 <- gDifference(gDifference(gIntersection(p2, p3), p1), p4)
highlight2<-as.data.frame(slot(int2@polygons[[1]]@Polygons[[1]], "coords"))
highlight2$group <- "1"

int3 <- gDifference(gDifference(gIntersection(p1, p2), p3), p4)
highlight3<-as.data.frame(slot(int3@polygons[[1]]@Polygons[[1]], "coords"))
highlight3$group <- "2"

int4 <- gDifference(gDifference(gIntersection(p3, p4), p1), p2)
highlight4<-as.data.frame(slot(int4@polygons[[1]]@Polygons[[1]], "coords"))
highlight4$group <- "2"

highlights <- rbind(highlight1, highlight2, highlight3, highlight4)


ellipses <- rbind(ellipse1, ellipse2, ellipse3, ellipse4)
ellipses$contrast <-
    as.factor(rep(
        c(
            "16dppT4vsT0_up",
            "8dppT4vsT0_up",
            "16dppT4vsT0_down",
            "8dppT4vsT0_down"
        ),
        each = 361
    ))
vennlabels <-
    data.frame(
        contrast = factor(
            c(
                "16dpp_T4vsT0_up",
                "8dpp_T4vsT0_up",
                "16dpp_T4vsT0_down",
                "8dpp_T4vsT0_down"
            )
        ),
        x = c(-2.85, -2, 2, 2.85),
        y = c(4.75, 5.5, 5.5, 4.75)
    )
```

```r
T4venn <- ggplot(data = venndf,
       aes(x = (x - y) / sqrt(2),
           y = (y + x) / sqrt(2))) +
    geom_path(data = ellipses,
              aes(
                  x = (V2 - V1) / sqrt(2),
                  y = (V1 + V2) / sqrt(2),
                  color = contrast
              ),
              size = 1) +
    geom_polygon(data = highlights,
                 aes(x = (y - x) / sqrt(2),
                     y = (y + x) / sqrt(2),
                     fill = group),
                 alpha = 0.25
    ) +
    scale_color_manual(values = c("#e41a1c", "#377eb8", "#4daf4a", "#984ea3")) +
    geom_text(
        data = vennlabels,
        aes(
            x = x,
            y = y,
            label = gsub('_', '\n', contrast)
            ),
        color = c("#377eb8", "#984ea3", "#e41a1c", "#4daf4a"),
        fontface = "bold"
    ) +
    geom_text(
        label = venndf$count,
        nudge_y = -0.10,
        fontface = "bold"
    ) +
    cowplot::theme_cowplot() +
    theme(
        legend.position = "none",
        axis.line = element_blank(),
        axis.text.x = element_blank(),
        axis.text.y = element_blank(),
        axis.ticks = element_blank(),
        axis.title.x = element_blank(),
        axis.title.y = element_blank(),
        panel.background = element_blank(),
        panel.border = element_blank(),
        panel.grid.major = element_blank(),
        panel.grid.minor = element_blank(),
        plot.background = element_blank(),
        plot.margin = unit(c(0.1, 0, 0, 0), "cm")
    ) +
    scale_x_continuous(expand = expand_scale(mult = 0.1)) +
    scale_y_continuous(expand = expand_scale(mult = 0.1)) +
    coord_cartesian(clip = "off", expand = TRUE)
```

GO terms of uniquely upregulated genes in 16dpp at T4vsT0

```
tgd <- runTopGoAnalysis(DEgeneSet = OLlist$Venn_List$`T4 vs T0 16dpp Up`, dds = dds_exp1, GOdb = GOList
kable(exportGOtable(tgd, "Fisher.weight01", n = 10) %>%
          mutate(Fisher.weight01 = as.numeric(Fisher.weight01)), digits = 20) %>%
    kableExtra::kable_styling(latex_options = c("HOLD_position", "scale_down"),
                              full_width = FALSE)
```

GO.ID	Term	Annotated	Significant	Expected	Fisher.weight01
GO:0009611	response to wounding	259	29	8.49	7.90e-09
GO:0015833	peptide transport	601	13	19.69	6.30e-06
GO:1901605	alpha-amino acid metabolic process	276	20	9.04	5.90e-05
GO:0009414	response to water deprivation	318	23	10.42	3.40e-04
GO:1901565	organonitrogen compound catabolic proces...	489	25	16.02	3.80e-04
GO:0010200	response to chitin	134	13	4.39	4.60e-04
GO:1901698	response to nitrogen compound	218	22	7.14	1.23e-03
GO:0046395	carboxylic acid catabolic process	131	12	4.29	1.25e-03
GO:0040007	growth	534	24	17.49	1.27e-03
GO:0009620	response to fungus	308	22	10.09	1.68e-03

Write these genes and their Arabidopsis best hits (from cucurbitgenomics.org) to a csv (Supp File 1):

```
timeContrasts %>%
    filter(
        padj < 0.05 & abs(log2FoldChange) >= 1,
        geneid %in% OLlist$Venn_List$`T4 vs T0 16dpp Up`,
        Age == "16dpp",
        Contrast == "T4 vs T0"
    ) %>%
    rowwise() %>%
    mutate(Annotation = paste(html_text(
        html_nodes(
            read_html(paste0(
                "http://cucurbitgenomics.org/feature/gene/", str_replace(geneid, " ", "")
            )),
            "#11 .odd:nth-child(1) td:nth-child(1) , #11 .odd:nth-child(1) td:nth-child(2) , #11 .odd:n
        )
    ), collapse = "__")) %>%
    separate(
        Annotation,
        into = c("Match Name", "E-value", "Identity", "Description"),
        sep = "__"
    ) %>%
    write.csv(file = "Supplementary File 1_Genes_Up16dppT4")
```

GO terms of uniquely upregulated genes in 8dpp at T4vsT0

```
tgd <- runTopGoAnalysis(DEgeneSet = OLlist$Venn_List$`T4 vs T0 8dpp Up`,
                        dds = dds_exp1,
                        GOdb = GOList,
                        onts = "BP",
                        nodeSize = 100)
kable(exportGOtable(tgd, "Fisher.weight01", n = 10) %>%
          mutate(Fisher.weight01 = as.numeric(Fisher.weight01)), digits = 20) %>%
    kableExtra::kable_styling(latex_options = c("HOLD_position", "scale_down"),
                              full_width = FALSE)
```

GO.ID	Term	Annotated	Significant	Expected	Fisher.weight01
GO:0044703	multi-organism reproductive process	136	8	2.67	7.10e-06
GO:0006518	peptide metabolic process	643	10	12.61	9.90e-05
GO:0019748	secondary metabolic process	251	12	4.92	1.40e-04
GO:0007154	cell communication	1524	41	29.88	1.80e-04
GO:0009636	response to toxic substance	152	11	2.98	2.10e-04
GO:0006979	response to oxidative stress	380	19	7.45	7.90e-04
GO:0006575	cellular modified amino acid metabolic p...	102	8	2.00	8.90e-04
GO:0046686	response to cadmium ion	335	16	6.57	9.80e-04
GO:0009651	response to salt stress	524	21	10.27	1.61e-03
GO:1901565	organonitrogen compound catabolic proces...	489	13	9.59	1.77e-03

```
venninput <- timeContrasts %>%
    filter(padj < 0.05 & abs(log2FoldChange) >= 1) %>%
    filter(Contrast == "T24 vs T4") %>%
    mutate(Direction = fct_relevel(ifelse(log2FoldChange > 0, "Up", "Down"), "Up")) %>%
    group_by(Contrast, Age, Direction) %>%
    do(data = (.$geneid)) %>%
    arrange(Direction, desc(Age)) %>%
    with(set_names(data, paste(Contrast, Age, Direction)))

OLlist <-
    overLapper(setlist = venninput,
               sep = "_",
               type = "vennsets")

counts <- list(sapply(OLlist$Venn_List, length))

venndf <- data.frame(
    count = as.vector(lengths(OLlist$Venn_List)),
    y = c(4, 4, 3, 1, 4, 3, 1, 3, 1, 2, 3, 1, 2, 2, 2),
    x = c(1, 3, 4, 4, 2, 1, 1, 3, 3, 4, 2, 2, 1, 3, 2)
)

ellipses <- rbind(ellipse1, ellipse2, ellipse3, ellipse4)
ellipses$contrast <-
    as.factor(rep(
        c(
            "16dppT24vsT4_up",
            "8dppT24vsT4_up",
            "16dppT24vsT4_down",
            "8dppT24vsT4_down"
        ),
        each = 361
    ))
vennlabels <-
    data.frame(
        contrast = factor(
            c(
                "16dpp_T24vsT4_up",
                "8dpp_T24vsT4_up",
                "16dpp_T24vsT4_down",
                "8dpp_T24vsT4_down"
            )
```

```
        ),
        x = c(-2.85, -2, 2, 2.85),
        y = c(4.75, 5.5, 5.5, 4.75)
    )

T24venn <- ggplot(data = venndf,
       aes(x = (x - y) / sqrt(2),
           y = (y + x) / sqrt(2))) +
    geom_path(data = ellipses,
              aes(
                  x = (V2 - V1) / sqrt(2),
                  y = (V1 + V2) / sqrt(2),
                  color = contrast
              ),
              size = 1) +
    geom_polygon(data = highlights,
                 aes(x = (y - x) / sqrt(2),
                     y = (y + x) / sqrt(2),
                     fill = group),
                 alpha = 0.25
    ) +
    scale_color_manual(values = c("#e41a1c", "#377eb8", "#4daf4a", "#984ea3")) +
    geom_text(
        data = vennlabels,
        aes(
            x = x,
            y = y,
            label = gsub('_', '\n', contrast)
            ),
        color = c("#377eb8", "#984ea3", "#e41a1c", "#4daf4a"),
        fontface = "bold"
    ) +
    geom_text(
        label = venndf$count,
        nudge_y = -0.10,
        fontface = "bold"
    ) +
    cowplot::theme_cowplot() +
    theme(
        legend.position = "none",
        axis.line = element_blank(),
        axis.text.x = element_blank(),
        axis.text.y = element_blank(),
        axis.ticks = element_blank(),
        axis.title.x = element_blank(),
        axis.title.y = element_blank(),
        panel.background = element_blank(),
        panel.border = element_blank(),
        panel.grid.major = element_blank(),
        panel.grid.minor = element_blank(),
        plot.background = element_blank(),
        plot.margin = unit(c(0.1, 0, 0, 0), "cm")
    ) +
```

```
    scale_x_continuous(expand = expand_scale(mult = 0.1)) +
    scale_y_continuous(expand = expand_scale(mult = 0.1)) +
    coord_cartesian(clip = "off", expand = TRUE)
```

GO terms of inversely regulated genes at 24 hpi (Up 16 down 8dpp)

```
tgd <- runTopGoAnalysis(DEgeneSet = OLlist$Venn_List$`T24 vs T4 16dpp Up_T24 vs T4 8dpp Down`,
                        dds = dds_exp1,
                        GOdb = GOList,
                        onts = "BP",
                        nodeSize = 100)

kable(exportGOtable(tgd, "Fisher.weight01", n = 10) %>%
        mutate(Fisher.weight01 = as.numeric(Fisher.weight01)), digits = 20) %>%
    kableExtra::kable_styling(latex_options = c("HOLD_position", "scale_down"),
                              full_width = FALSE)
```

GO.ID	Term	Annotated	Significant	Expected	Fisher.weight01
GO:0019684	photosynthesis, light reaction	115	17	1.63	5.20e-13
GO:0055114	oxidation-reduction process	1249	35	17.68	7.60e-05
GO:0048869	cellular developmental process	662	12	9.37	9.30e-04
GO:0006732	coenzyme metabolic process	262	11	3.71	2.05e-03
GO:0007169	transmembrane receptor protein tyrosine ...	135	7	1.91	3.11e-03
GO:0009658	chloroplast organization	148	7	2.10	5.15e-03
GO:0006790	sulfur compound metabolic process	256	8	3.62	5.22e-03
GO:0015979	photosynthesis	201	22	2.85	5.58e-03
GO:0048229	gametophyte development	312	12	4.42	6.40e-03
GO:0043603	cellular amide metabolic process	751	14	10.63	8.06e-03

```
venninput <- timeContrasts %>%
    filter(padj < 0.05 & abs(log2FoldChange) >= 1) %>%
    filter(Contrast == "T48 vs T24") %>%
    mutate(Direction = fct_relevel(ifelse(log2FoldChange > 0, "Up", "Down"), "Up")) %>%
    group_by(Contrast, Age, Direction) %>%
    do(data = (.$geneid)) %>%
    arrange(Direction, desc(Age)) %>%
    with(set_names(data, paste(Contrast, Age, Direction)))


OLlist <-
    overLapper(setlist = venninput,
               sep = "_",
               type = "vennsets")

counts <- list(sapply(OLlist$Venn_List, length))

venndf <- data.frame(
    count = as.vector(lengths(OLlist$Venn_List)),
    y = c(4, 4, 3, 1, 4, 3, 1, 3, 1, 2, 3, 1, 2, 2, 2),
    x = c(1, 3, 4, 4, 2, 1, 1, 3, 3, 4, 2, 2, 1, 3, 2)
)

ellipses <- rbind(ellipse1, ellipse2, ellipse3, ellipse4)
ellipses$contrast <-
```

```
    as.factor(rep(
        c(
            "16dppT48vsT24_up",
            "8dppT48vsT24_up",
            "16dppT48vsT24_down",
            "8dppT48vsT24_down"
        ),
        each = 361
    ))

vennlabels <-
    data.frame(
        contrast = factor(
            c(
                "16dpp_T48vsT24_up",
                "8dpp_T48vsT24_up",
                "16dpp_T48vsT24_down",
                "8dpp_T48vsT24_down"
            )
        ),
        x = c(-2.85, -2, 2, 2.85),
        y = c(4.75, 5.5, 5.5, 4.75)
    )

T48venn <- ggplot(data = venndf,
      aes(x = (x - y) / sqrt(2),
          y = (y + x) / sqrt(2))) +
    geom_path(data = ellipses,
              aes(
                  x = (V2 - V1) / sqrt(2),
                  y = (V1 + V2) / sqrt(2),
                  color = contrast
              ),
              size = 1) +
    geom_polygon(data = highlights,
                 aes(x = (y - x) / sqrt(2),
                     y = (y + x) / sqrt(2),
                     fill = group),
                 alpha = 0.25
    ) +
    scale_color_manual(values = c("#e41a1c", "#377eb8", "#4daf4a", "#984ea3")) +
    geom_text(
        data = vennlabels,
        aes(
            x = x,
            y = y,
            label = gsub('_', '\n', contrast)
            ),
        color = c("#377eb8", "#984ea3", "#e41a1c", "#4daf4a"),
        fontface = "bold"
    ) +
    geom_text(
        label = venndf$count,
```

```
        nudge_y = -0.10,
        fontface = "bold"
    ) +
    cowplot::theme_cowplot() +
    theme(
        legend.position = "none",
        axis.line = element_blank(),
        axis.text.x = element_blank(),
        axis.text.y = element_blank(),
        axis.ticks = element_blank(),
        axis.title.x = element_blank(),
        axis.title.y = element_blank(),
        panel.background = element_blank(),
        panel.border = element_blank(),
        panel.grid.major = element_blank(),
        panel.grid.minor = element_blank(),
        plot.background = element_blank(),
        plot.margin = unit(c(0.1, 0, 0, 0), "cm")
    ) +
    scale_x_continuous(expand = expand_scale(mult = 0.1)) +
    scale_y_continuous(expand = expand_scale(mult = 0.1)) +
    coord_cartesian(clip = "on", expand = TRUE)
```

```
vennPlots <- cowplot::plot_grid(T4venn, T24venn, T48venn,
                                label_size = 16,
                                labels = c("C", "D", "E"),
                                align = "v",
                                axis = "l",
                                ncol = 1,
                                rel_widths = c(0.1, 1, 1, 1))
```

Make Figure 2 PCA, Summary of DEGs and Venn diagrams:

```
expPlots <- cowplot::plot_grid(pcaPlot1, sumPlot1,
                               align = "v",
                               labels = c("A", "B"),
                               label_size = 16,
                               axis = "l",
                               ncol = 1
                               )

cowplot::plot_grid(
    expPlots,
    NULL,
    vennPlots,
    ncol = 3,
    align = "v",
    axis = "b",
    rel_widths = c(2, 0.1, 1.5)
)
```

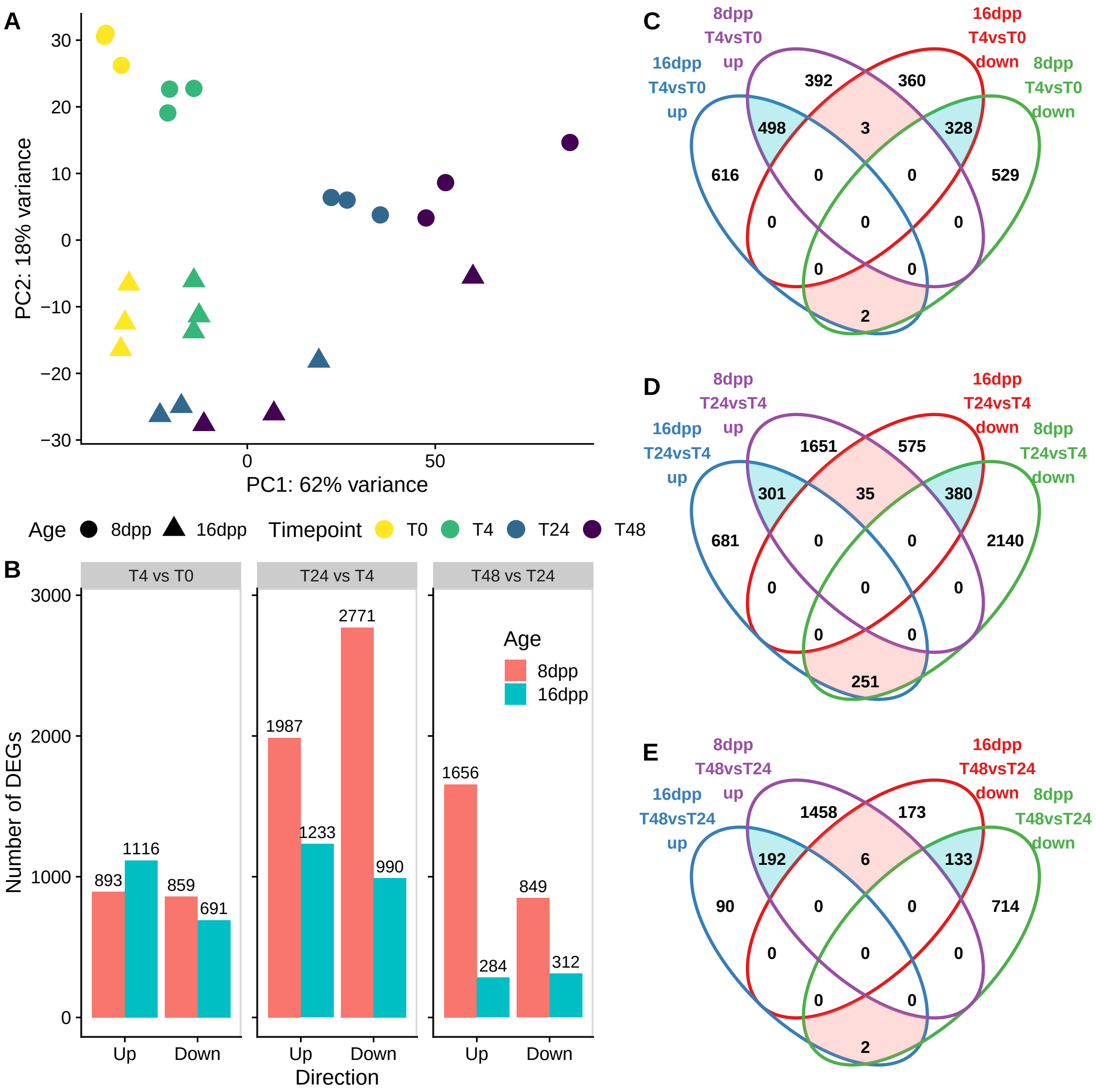


```
pdf("fig2.pdf", width = 12, height = 10)
cowplot::plot_grid(expPlots, NULL, vennPlots, ncol = 3, rel_widths = c(2, 0.1, 1.5))
dev.off()
```

```
## pdf
##   2
```

Transciptome Experiment 2

QuantSeq 3' mRNA mapping pipeline

Read cleanup and Quality control

```
mkdir infection24_3prime
cd infection24_3prime
mkdir qualitycheck

# To install multiQC we need to work with Anaconda python environment
module load Anaconda2/4.2.0
export PATH=$PATH:$HOME/anaconda2/bin
conda create --name multiQC
source activate multiQC

# Install the multiQC package
conda install -c bioconda multiqc
source deactivate

# QC before cleaning
module load fastQC
fastqc --outdir qualitycheck --format fastq --threads 8 *fastq.gz

export PATH=$PATH:$HOME/anaconda2/bin
source activate multiQC
multiqc -o ~/infection24_3prime/ -n multiqc_raw ~/infection24_3prime/qualitycheck/
source deactivate

# Make a polyA fasta file for trimming
printf '>\nAAAAAAAAAAAAAAAAAAA' > polyA.fa
gzip polyA.fa

# Trim and clean reads with BBMap
module load BBMap
for sample in *fastq.gz;
do cat $sample | bbduk.sh in=stdin.fq.gz out=${sample}_trimmed_clean \
ref=polyA.fa.gz,$ADAPTERS/TruSeq3-SE.fa \
ftl=12 k=13 ktrim=r useshortkmers=t mink=5 qtrim=r trimq=10 minlength=20 int=f ;
done

# QC after trimming
mkdir qc_trimmed_clean

fastqc --outdir qc_trimmed_clean --format fastq --threads 8 *fastq.gz_trimmed_clean

export PATH=$PATH:$HOME/anaconda2/bin
source activate multiQC
multiqc -o ~/infection24_3prime/ \
-n multiqc_clean ~/infection24_3prime/qc_trimmed_clean/
source deactivate
```

Make extended transcriptome file

Extract chromosome sizes:

```
wget https://github.com/bedops/bedops/releases/download/v2.4.35/bedops_linux_x86_64-v2.4.35.tar.bz2
tar jxvf bedops_linux_x86_64-v2.4.35.tar.bz2
export PATH=$PATH:$HOME/bin

cd ~/infectionRNAseq/genome/cl9930-v2
gff2bed < cucumber_ChineseLong_v2.gff3 > cucumber_ChineseLong_v2.bed

module load SAMtools
cut -f 1,2 cucumber_ChineseLong_v2_genome.fa.fai > chrom.sizes
```

Transcript 3'UTR extension script in R:

```
gff <- read.delim('cucumber_ChineseLong_v2.gff3', header = FALSE, sep = '\t')
chromSizes <- read.table(file = "chrom.sizes") %>%
  dplyr::rename(chrom = V1,
         maxLength = V2)

names(gff) <- c("chrom", "source", "feature",
                "start", "end", "score",
                "strand", "phase", "attr")

gff <- gff %>%
  mutate(parent = ifelse(str_detect(attr, "CsaUN"),
                       str_remove(str_extract(string = attr,
                                              pattern = "ID=CsaUNG[0-9]*"),
                                  pattern = "ID="),
                       str_remove(str_extract(string = attr,
                                              pattern = "ID=Csa[0-9]G[0-9]*"),
                                  pattern = "ID=")
                       )
         )

extend <- 1000
gff_name <- paste0("cucumber_ChineseLong_v2_extended3UTR_", extend,".gff3")

gff_new <- left_join(gff, chromSizes) %>%
  group_by(parent, feature) %>%
  mutate(id = row_number()) %>% # add id index for each feature
  mutate(
    sug_start = case_when(
      feature %in% c("gene", "mRNA") &
        strand == "-" ~ start - extend,
      feature == "three_prime_utr" &
        id == max(id) & strand == "-" ~ start - extend, #use max id to get the last 3'UTR
      feature == "exon" &
        id == max(id) & strand == "-" ~ start - extend, #use max id to get the last exon,
      TRUE ~ as.numeric(start)
    )
  ) %>%
  mutate(
    sug_end = case_when(
      feature %in% c("gene", "mRNA") &
        strand == "+" ~ end + extend,
      feature == "three_prime_utr" &
```

```
        id == max(id) & strand == "+" ~ end + extend, #use max id to get the last 3'UTR
      feature == "exon" &
        id == max(id) & strand == "+" ~ end + extend, #use max id to get the last exon
      TRUE ~ as.numeric(end)
    )
  )  %>%
  ungroup() %>%
  mutate(sug_start = as.integer(ifelse(sug_start <= 0, 1, sug_start)),  # limit on the left
         sug_end = as.integer(ifelse(sug_end > maxLength, maxLength, sug_end)) # limit on the right
  )

gff_new2 <- gff_new %>% # Find the distances between genes and the next start
  left_join(gff_new %>% # site on the same strand
              group_by(chrom, strand) %>%
              arrange(chrom, strand, start) %>%
              filter(feature %in% c("gene")) %>%
              mutate(next_gene = ifelse(strand == "-",
                                        lag(end, 1),
                                        lead(start, 1))
              ) %>% mutate(next_gene = ifelse(is.na(next_gene),
                                         ifelse(strand == "-", 0, maxLength + 1),
                                         next_gene))
            ) %>%
  group_by(chrom) %>%
    fill(next_gene) %>% # Identify suggested start sites that overlap the next gene
    mutate(
    start_ext = case_when(
      feature %in% c("gene", "mRNA") &
        sug_start <= next_gene & strand == "-" ~ next_gene + 1, # if there is an overlap stop one base
      feature == "three_prime_utr" &
        id == max(id) & sug_start <= next_gene & strand == "-" ~ next_gene + 1, #use max id to get the
      feature == "exon" &
        id == max(id) & sug_start <= next_gene & strand == "-" ~ next_gene + 1, #use max id to get the
      TRUE ~ as.numeric(sug_start)
    )
  ) %>%
  mutate(
    end_ext = case_when(
      feature %in% c("gene", "mRNA") &
        sug_end >= next_gene & strand == "+" ~ next_gene - 1,
      feature == "three_prime_utr" &
        id == max(id) & sug_end >= next_gene & strand == "+" ~ next_gene - 1, #use max id to get the la
      feature == "exon" &
        id == max(id) & sug_end >= next_gene & strand == "+" ~ next_gene - 1, #use max id to get the la
      TRUE ~ as.numeric(sug_end)
    )
  ) %>%
  ungroup() %>%
  mutate(start_ext = as.integer(ifelse(start_ext <= 0, 1, start_ext)),  # limit on the left
         end_ext = as.integer(ifelse(end_ext > maxLength, maxLength, end_ext)) # limit on the right
  ) %>%
  mutate(delta = (end_ext - start_ext) - (end - start)) %>% # check the difference before and after ext
  mutate(final_start = ifelse(delta < 0, start, start_ext),
```

```
         final_end = ifelse(delta < 0, end, end_ext),
         final_delta = (final_end - final_start) - (end - start))

gff_new2 %>%
  filter(feature == "gene") %>%
  ggplot() +
  geom_density(aes(x = final_delta))

gff_out <- gff_new2 %>%
  mutate(start = final_start,
         end = final_end
         ) %>%
  dplyr::select(-parent, -id, -maxLength,
         -sug_start, -sug_end, -start_ext,
         -end_ext, -final_start, -final_end,
         -delta, -final_delta, -next_gene
         )

write_delim(gff_out,
            path = gff_name,
            delim = "\t",
            col_names = FALSE)
```

Use cufflinks gffread to output a fasta file based on the new gff3 file:

```
module load Cufflinks/2.2.1
gffread -g ../genome/cl9930-v2/cucumber_ChineseLong_v2_genome.fa -w cucumber_ChineseLong_v2_cdna_extend[illegible]
```

Salmon mapping

```
wget https://github.com/COMBINE-lab/salmon/releases/download/v0.12.0/salmon-0.12.0_linux_x86_64.tar.gz
tar xvfz salmon-0.12.0_linux_x86_64.tar.gz

SALMON=salmon-0.12.0_linux_x86_64/bin

# Index transcriptome
$SALMON/salmon index -t cucumber_ChineseLong_v2_cdna_extended3UTR_1000.fa -i cucumber_ChineseLong_v2_cd[illegible]

# Perform the mapping using Salmon
mkdir quants_1000

for fn in `ls clean_reads/*fastq`;
do
nodir=${fn#clean_reads/}
echo "Processing sample ${nodir}"
$SALMON/salmon quant -i cucumber_ChineseLong_v2_cdna_extended3UTR_1000_salmon_index \
    -l SF \
    -r ${fn} \
    -p 16 \
    -o quants_1000/${nodir%_S*}_quant \
    --noLengthCorrection \
    --validateMappings;
done
```

Differential expression pipeline

Import data using tximport

```
dirs <- list.files("./quants_1000")
samples <- as_tibble(stringr::str_split(dirs, pattern = "\\.", n = 2, simplify = T)[, 1]) %>%
  separate(col = value, into = c("well", "age", "timepoint", "treatment", "rep"), sep = "_", remove = F
  dplyr::rename(name = value) %>%
  mutate(directory = dirs)

# duplicate the controll T0 samples to have Inoc T0 for statistical purposes
fake_inocT0 <- samples %>%
  filter(timepoint == "T0") %>%
  mutate(treatment = "Inoc",
         name = str_replace(name, "Cont", "InocFake"))

samples <- bind_rows(samples, fake_inocT0)

files <- file.path("quants_1000", samples$directory, "quant.sf")
names(files) <- samples$name

samples <- samples %>%
  mutate(
  timepoint = fct_relevel(timepoint, "T0", "T2", "T4", "T8", "T12", "T18", "T24"),
  age = fct_relevel(age, "8dpp"),
  treatment = fct_relevel(samples$treatment, "Cont"),
  condition = as_factor(
  paste0(samples$age, "_", samples$timepoint, "_", samples$treatment)
  ),
  condition = fct_relevel(
  condition,
  "8dpp_T0_Cont",
  "8dpp_T2_Cont",
  "8dpp_T4_Cont",
  "8dpp_T8_Cont",
  "8dpp_T12_Cont",
  "8dpp_T18_Cont",
  "8dpp_T24_Cont",
  "16dpp_T0_Cont",
  "16dpp_T2_Cont",
  "16dpp_T4_Cont",
  "16dpp_T8_Cont",
  "16dpp_T12_Cont",
  "16dpp_T18_Cont",
  "16dpp_T24_Cont",
  "8dpp_T0_Inoc",
  "8dpp_T2_Inoc",
  "8dpp_T4_Inoc",
  "8dpp_T8_Inoc",
  "8dpp_T12_Inoc",
  "8dpp_T18_Inoc",
  "8dpp_T24_Inoc",
  "16dpp_T0_Inoc",
  "16dpp_T2_Inoc",
```

```
  "16dpp_T4_Inoc",
  "16dpp_T8_Inoc",
  "16dpp_T12_Inoc",
  "16dpp_T18_Inoc",
  "16dpp_T24_Inoc"
  )
  )

# make tx2gene file
tx2gene <- read.table(file = files[1], sep = "\t", header = TRUE)
tx2gene <- tx2gene[1]
tx2gene$geneid <- str_replace(tx2gene$Name, pattern = "\\..*", replacement = "")

txiAll <- tximport(files, type = "salmon", tx2gene = tx2gene, dropInfReps = TRUE, countsFromAbundance =

ddsTxiAll <- DESeqDataSetFromMatrix(round(txiAll$counts), colData = samples, design = ~ condition)

# filter low expression genes: genes were 75 samples have less than 5 reads
ddsTxiAll_filt <- ddsTxiAll[rowSums(counts(ddsTxiAll) <= 5) < 75, ]
```

Reads mapping stats

```
# Import JSON files for sample read mapping results

jsons <- file.path("quants_1000", samples$directory, "aux_info", "meta_info.json")
samples$totalReads <- sapply(jsons, FUN = function(x) {
    fromJSON(file = x)$num_processed
})

samples$Mapped <- sapply(jsons, FUN = function(x) {
    fromJSON(file = x)$num_mapped
})
```

```
expDesign <- samples %>%
    group_by(age, timepoint, treatment) %>% summarise(Reps = n())

knitr::kable(as.data.frame(expDesign), caption = "The experimental design") %>%
    kable_styling(bootstrap_options = "striped", full_width = FALSE)
```

```
samples %>%
    mutate(sampleName = name,
           Unmapped = totalReads - Mapped) %>%
    arrange(age, timepoint) %>%
    mutate(sampleName = fct_inorder(sampleName)) %>%
    gather(key = "Reads", value = "Count", -directory, -age, -timepoint, -condition, -sampleName, -total
    ggplot(aes(x = sampleName, y = Count, fill = Reads,label = paste0(round(Count / totalReads * 100, di
    geom_bar(stat = "identity", position = "stack") +
    geom_text(size = 2, stat = "identity", position = "stack", hjust = 0.5) +
    theme(axis.text.x = element_text(angle = 90, vjust = 0.5))
```

Table 2: The experimental design

age	timepoint	treatment	Reps
8dpp	T0	Cont	3
8dpp	T0	Inoc	3
8dpp	T2	Cont	3
8dpp	T2	Inoc	3
8dpp	T4	Cont	3
8dpp	T4	Inoc	3
8dpp	T8	Cont	3
8dpp	T8	Inoc	3
8dpp	T12	Cont	3
8dpp	T12	Inoc	3
8dpp	T18	Cont	3
8dpp	T18	Inoc	3
8dpp	T24	Cont	3
8dpp	T24	Inoc	3
16dpp	T0	Cont	3
16dpp	T0	Inoc	3
16dpp	T2	Cont	3
16dpp	T2	Inoc	3
16dpp	T4	Cont	3
16dpp	T4	Inoc	3
16dpp	T8	Cont	3
16dpp	T8	Inoc	3
16dpp	T12	Cont	3
16dpp	T12	Inoc	3
16dpp	T18	Cont	3
16dpp	T18	Inoc	3
16dpp	T24	Cont	3
16dpp	T24	Inoc	3

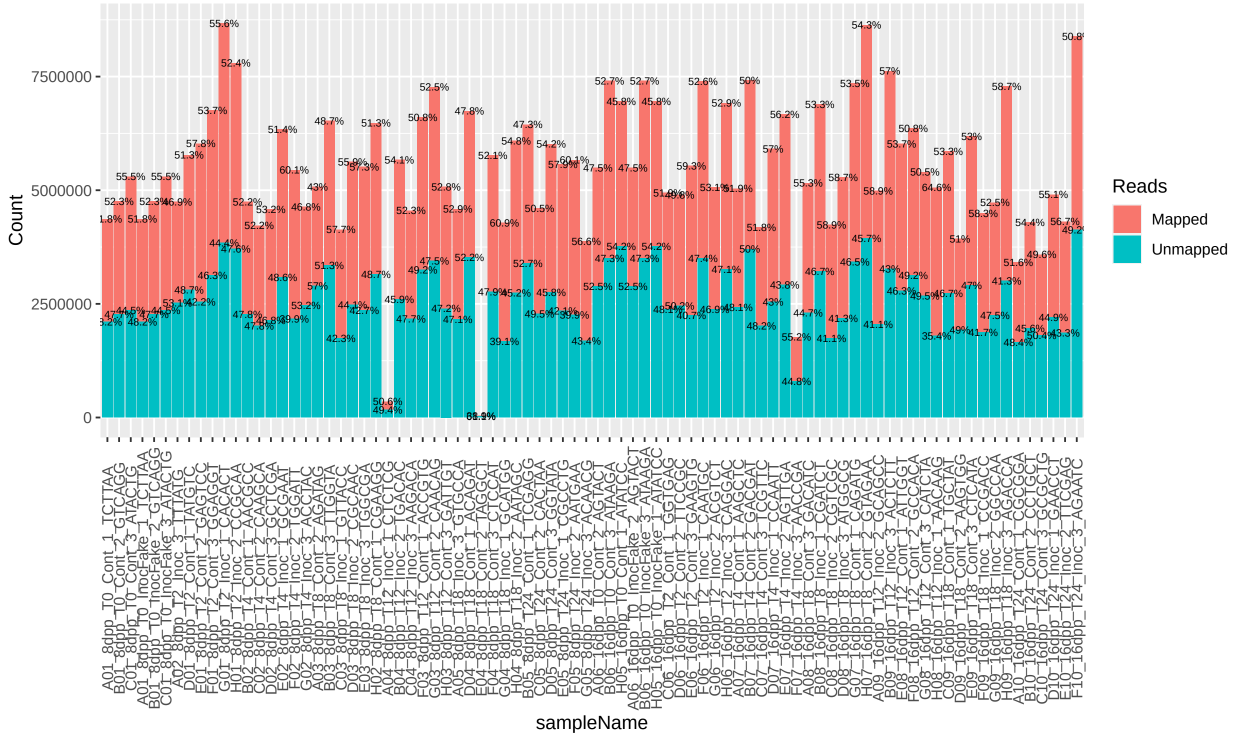


```r
# remove samples with less than 1M reads

ddsTxiAll_filt <- ddsTxiAll_filt[, samples$totalReads > 1e6]

# average reads mapping

samples %>%
  filter(totalReads > 1e6) %>%
  summarize(avgReadMapping = mean(Mapped / totalReads))
```

```
## # A tibble: 1 x 1
##   avgReadMapping
##            <dbl>
## 1          0.533
```

DESeq2 analysis

```r
dds <- DESeq(ddsTxiAll_filt)

save(dds, file = "infectionTC_8vs16_ContvsInoc_dds.RData")
```

Principal component analysis

```r
vst <- vst(dds, blind = TRUE)

pcaData <- plotPCA(vst, returnData=TRUE, ntop = 500)

percentVar <- round(100 * attr(pcaData, "percentVar"))

pcaData <- pcaData %>%
    mutate(Age = vst$age,
```

```
          Timepoint = vst$timepoint,
          Treatment = vst$treatment)

pcaExp2 <- ggplot(pcaData, aes(PC1, PC2, color = Timepoint, shape = Age)) +
    geom_point(size = 4, stroke = 2) +
    geom_point(aes(alpha = Treatment, fill = Timepoint), size = 5, stroke = 0) +
    scale_shape_manual(values = c(21, 24, 21, 24)) +
    scale_alpha_manual(values=c(0.05, 1, 0.05, 1)) +
    xlab(paste0("PC1: ",percentVar[1],"% variance")) +
    ylab(paste0("PC2: ",percentVar[2],"% variance")) +
    geom_point(data = filter(pcaData, Timepoint == "T0"),
               aes(x = PC1, y = PC2, shape = Timepoint),
               color = "black",
               shape = 8,
               size = 2) +
    cowplot::theme_cowplot() +
    guides(
    shape = guide_legend(order = 1),
    fill = guide_legend(order = 0, nrow=2, byrow=TRUE),
    alpha = guide_legend(title = "Treatment",
                                override.aes = list(shape = c(22, 15), stroke = 2, alpha = 1))
  ) +
  scale_color_viridis_d(direction = -1) +
  scale_fill_viridis_d(direction = -1) +
    theme(legend.position = "bottom",
          legend.direction = "horizontal") +
    coord_fixed(ratio = percentVar[2] / percentVar[1])

pcaExp2
```

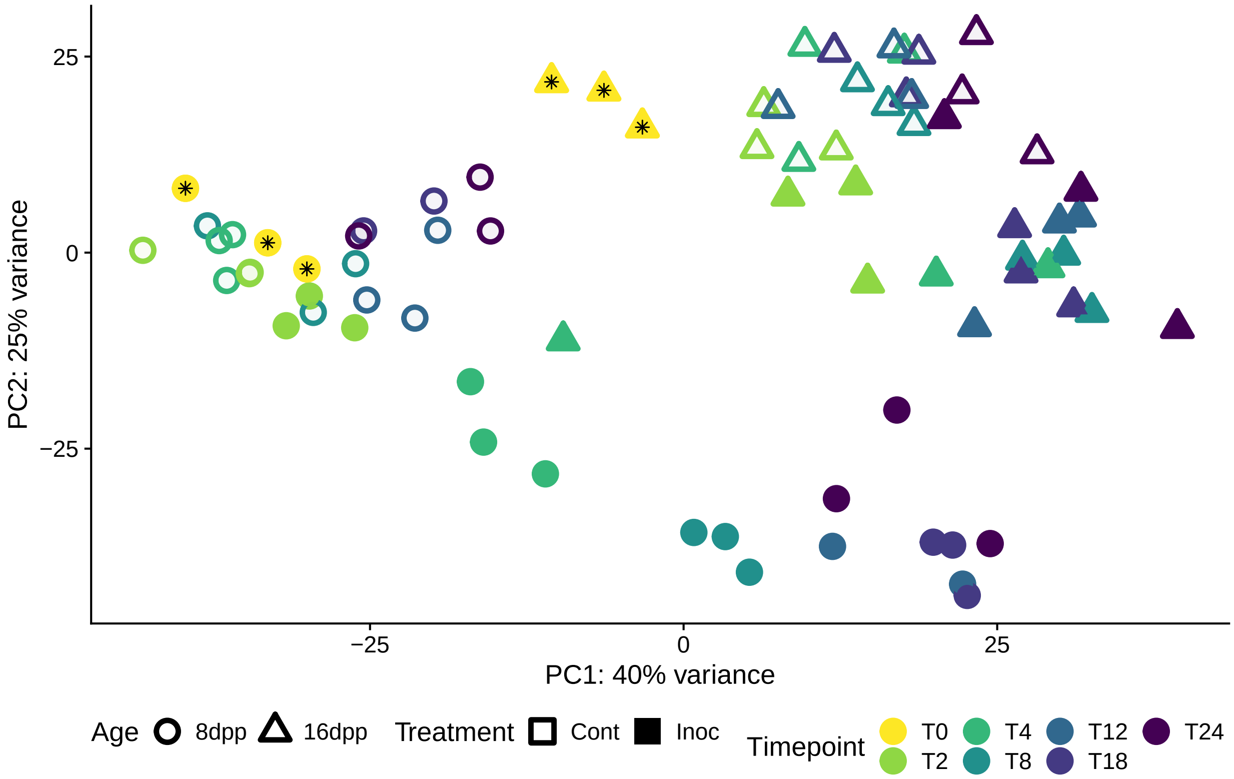

Differential expression contrast results

```
IvsC_T2_8dpp <- lfcShrink(dds, contrast = c("condition", "8dpp_T2_Inoc", "8dpp_T2_Cont"))
IvsC_T4_8dpp <- lfcShrink(dds, contrast = c("condition", "8dpp_T4_Inoc", "8dpp_T4_Cont"))
IvsC_T8_8dpp <- lfcShrink(dds, contrast = c("condition", "8dpp_T8_Inoc", "8dpp_T8_Cont"))
IvsC_T12_8dpp <- lfcShrink(dds, contrast = c("condition", "8dpp_T12_Inoc", "8dpp_T12_Cont"))
IvsC_T18_8dpp <- lfcShrink(dds, contrast = c("condition", "8dpp_T18_Inoc", "8dpp_T18_Cont"))
IvsC_T24_8dpp <- lfcShrink(dds, contrast = c("condition", "8dpp_T24_Inoc", "8dpp_T24_Cont"))

IvsC_T2_16dpp <- lfcShrink(dds, contrast = c("condition", "16dpp_T2_Inoc", "16dpp_T2_Cont"))
IvsC_T4_16dpp <- lfcShrink(dds, contrast = c("condition", "16dpp_T4_Inoc", "16dpp_T4_Cont"))
IvsC_T8_16dpp <- lfcShrink(dds, contrast = c("condition", "16dpp_T8_Inoc", "16dpp_T8_Cont"))
IvsC_T12_16dpp <- lfcShrink(dds, contrast = c("condition", "16dpp_T12_Inoc", "16dpp_T12_Cont"))
IvsC_T18_16dpp <- lfcShrink(dds, contrast = c("condition", "16dpp_T18_Inoc", "16dpp_T18_Cont"))
IvsC_T24_16dpp <- lfcShrink(dds, contrast = c("condition", "16dpp_T24_Inoc", "16dpp_T24_Cont"))

timeContrasts <-
  bind_rows(
  "IvsC_T2_8dpp" = as.data.frame(IvsC_T2_8dpp),
  "IvsC_T4_8dpp" = as.data.frame(IvsC_T4_8dpp),
  "IvsC_T8_8dpp" = as.data.frame(IvsC_T8_8dpp),
  "IvsC_T12_8dpp" = as.data.frame(IvsC_T12_8dpp),
  "IvsC_T18_8dpp" = as.data.frame(IvsC_T18_8dpp),
  "IvsC_T24_8dpp" = as.data.frame(IvsC_T24_8dpp),
  "IvsC_T2_16dpp" = as.data.frame(IvsC_T2_16dpp),
  "IvsC_T4_16dpp" = as.data.frame(IvsC_T4_16dpp),
  "IvsC_T8_16dpp" = as.data.frame(IvsC_T8_16dpp),
  "IvsC_T12_16dpp" = as.data.frame(IvsC_T12_16dpp),
```

```
  "IvsC_T18_16dpp" = as.data.frame(IvsC_T18_16dpp),
  "IvsC_T24_16dpp" = as.data.frame(IvsC_T24_16dpp)
  , .id = "Contrast") %>%
  mutate(geneid = rep(rownames(dds), 12))
```

Alluvial plots for all DE genes

```
lodesTC <- timeContrasts %>%
    mutate(Direction = case_when(
        padj < 0.05 & log2FoldChange >= 1 ~ "Up",
        padj < 0.05 & log2FoldChange <= -1 ~ "Down",
        TRUE ~ "notDE"
    )) %>%
    mutate(Direction = fct_relevel(Direction, "Down", "Up", "notDE"),
           Age = str_extract(Contrast, "[0-9]*dpp"),
           Contrast = str_remove(Contrast, "_[0-9]*dpp")) %>%
    mutate(Contrast = fct_inorder(Contrast),
           Age = fct_relevel(Age, "8dpp")
    ) %>%
    dplyr::select(geneid, Direction, Contrast, Age) %>%
    group_by(geneid, Age) %>%
    filter(sum(as.numeric(Direction)) < 18) %>% # because as.numeric('notDE') = 3
    summarise(flow = paste0(Direction, collapse = "_")) %>%
    group_by(Age, flow) %>%
    summarise(freq = n()) %>%
    separate(col = flow, into = c("T2", "T4", "T8", "T12", "T18", "T24")) %>%
    mutate(first = T4,
           second = T8,
           third = T12,
           forth = T18,
           fifth = T24,
           sixth = T24) %>%
    to_lodes_form(key = "Contrast", axes = 2:7) %>%
    mutate(diffExp = fct_relevel(stratum, "Up", "notDE")) %>%
    mutate(flowColor = case_when(
      Contrast == "T2" ~ first,
      Contrast == "T4" ~ second,
      Contrast == "T8" ~ third,
      Contrast == "T12" ~ forth,
      Contrast == "T18" ~ fifth,
      Contrast == "T24" ~ sixth
    )) %>%
  group_by(Age, Contrast, diffExp) %>%
  mutate(stratumSize = sum(freq),
         number = stratumSize/ n())

alluvialExp2 <- lodesTC%>%
    ggplot(
        aes(
            x = Contrast,
            stratum = diffExp,
            alluvium = alluvium,
            y = freq,
            fill = diffExp,
```

```
            label = freq
        )
    ) +
    geom_flow(
        aes(fill = as.factor(flowColor)),
        stat = "alluvium",
        alpha = 0.75,
        size = 0.5
    ) +
    geom_stratum(width = 0.5) +
    geom_text(aes(label = ifelse(stratumSize > 100, number, NA)),
              stat = "stratum",
              size = 3,
              position = position_nudge(y = 0)) +
    labs(x = "Timepoint vs. Control", y = "Number of genes") +
    scale_fill_manual(values = c("#fc8d62", "#8da0cb", "#66c2a5"),
                      breaks = c("Up", "notDE", "Down"),
                      name = "Differential\nexpression") +
    ggrepel::geom_text_repel(stat = "stratum",
                             size = 3,
                             nudge_y = 100,
                             aes(label = ifelse(stratumSize <= 100,
                                                number,
                                                NA
                                                )
                                 )
                             ) +
    cowplot::theme_cowplot(font_size = 14) +
    theme(strip.text = element_text(colour = "grey10",
                                    size = rel(0.8),
                                    margin = margin(0.5 * 7,
                                                    0.5 * 7,
                                                    0.5 * 7,
                                                    0.5 * 7)
                                    ),
          legend.position = c(0.85, 0.85)
          ) +
    lemon::facet_rep_wrap(~ Age) +
    cowplot::panel_border()

alluvialExp2
```

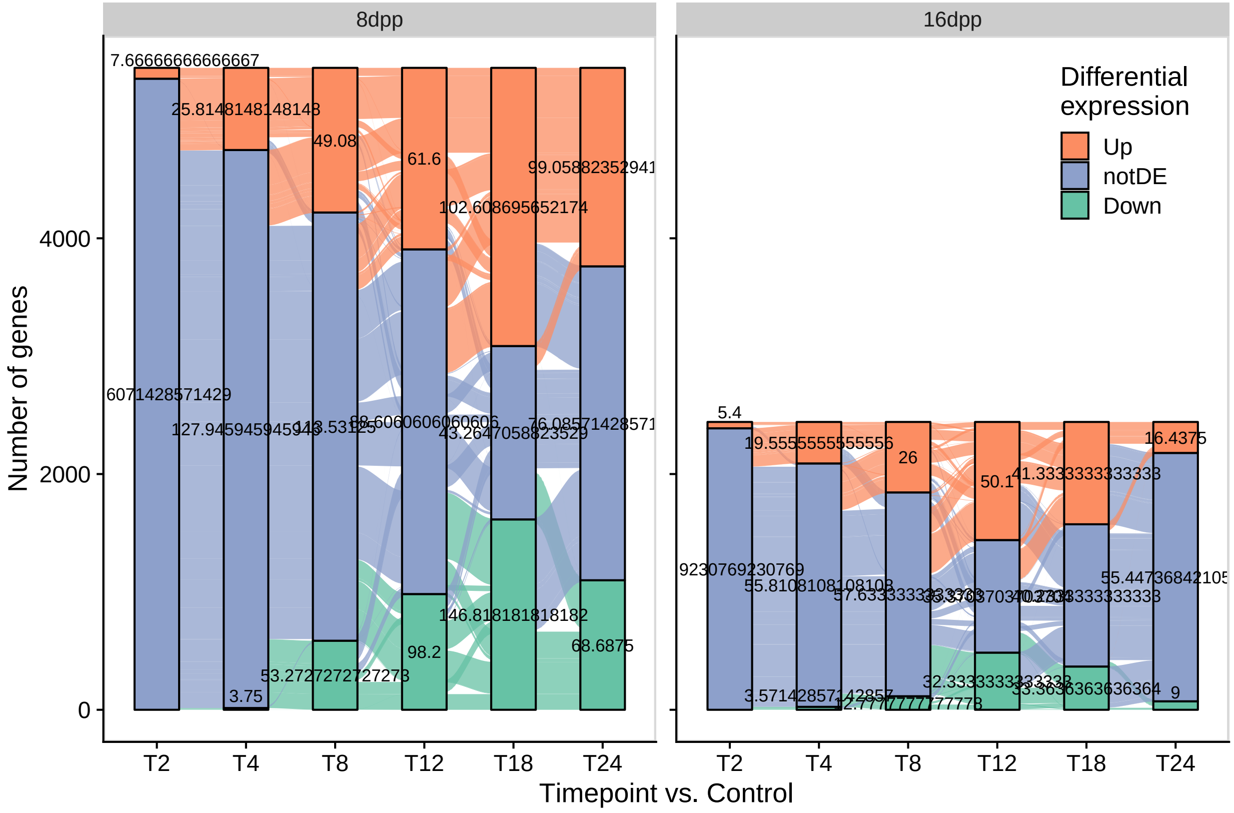

Get some summary numbers for alluvial results

```r
# upregulated genes at 4 hpi and are continuously upregulated in every subsequent timepoint
lodesTC %>%
    filter(Age == "8dpp", Contrast == "T4", diffExp == "Up") %>%
    filter_at(.vars = c("second","third", "forth", "fifth", "sixth"),
              all_vars(. == "Up")) %>%
    summarize(sum(freq))
```

```
## # A tibble: 1 x 4
## # Groups:   Age, Contrast [1]
##   Age   Contrast diffExp `sum(freq)`
##   <fct> <fct>    <fct>         <int>
## 1 8dpp  T4       Up              424
```

```r
# how many of the upregulated 8dpp genes are newly upregulated at T24
lodesTC %>%
    filter(Age == "8dpp", Contrast == "T24", diffExp == "Up") %>%
    filter_at(.vars = c("second","third", "forth"), all_vars(. != "Up")) %>%
    summarise(sum(freq))
```

```
## # A tibble: 1 x 4
## # Groups:   Age, Contrast [1]
##   Age   Contrast diffExp `sum(freq)`
##   <fct> <fct>    <fct>         <int>
## 1 8dpp  T24      Up              161
```

```r
# how many 16 dpp genes upregulated at 4 hpi, are not differentially expressed at 8 hpi
lodesTC %>%
```

```r
    filter(Age == "16dpp", Contrast == "T4", diffExp == "Up", second != "Up") %>%
    summarise(sum(freq))
```

```
## # A tibble: 1 x 4
## # Groups:   Age, Contrast [1]
##   Age   Contrast diffExp `sum(freq)`
##   <fct> <fct>    <fct>         <int>
## 1 16dpp T4       Up              140
```

```r
# how many continuously upregulated through 24 hpi
lodesTC %>%
    filter(Age == "16dpp", Contrast == "T4", diffExp == "Up") %>%
    filter_at(.vars = c("second","third", "forth", "fifth", "sixth"),
              all_vars(. == "Up")) %>%
    summarize(sum(freq))
```

```
## # A tibble: 1 x 4
## # Groups:   Age, Contrast [1]
##   Age   Contrast diffExp `sum(freq)`
##   <fct> <fct>    <fct>         <int>
## 1 16dpp T4       Up               45
```

```r
cowplot::plot_grid(pcaExp2, alluvialExp2, align = "v", axis = "l", ncol = 1, labels = "AUTO")
```

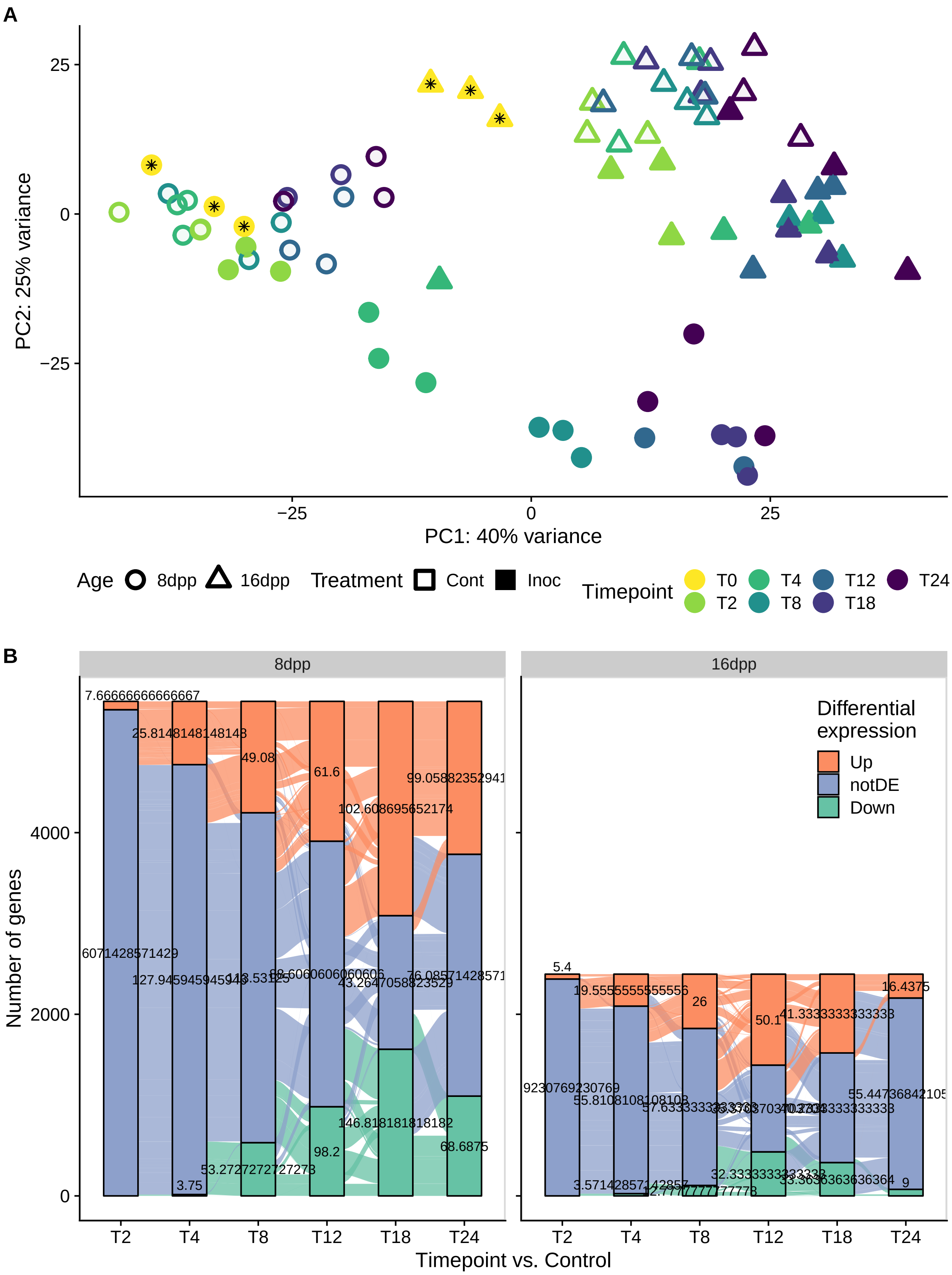
A
PC2: 25% variance
PC1: 40% variance
Age 8dpp 16dpp
Treatment Cont Inoc
Timepoint T0 T2 T4 T8 T12 T18 T24
B
8dpp
16dpp
Number of genes
Timepoint vs. Control
Differential expression
Up
notDE
Down

```r
pdf(file = "fig5.pdf", width = 9, height =  12)
cowplot::plot_grid(pcaExp2, alluvialExp2, align = "v", axis = "l", ncol = 1, labels = "AUTO")
dev.off()
```

```
## pdf
##   2
```

Just as a check we will compare the experiments using pca Supplemental Figure 4.

```r
vst_48hrs <- vst(dds_exp1, blind = TRUE)
vst_48hrs <- vst_48hrs[, vst_48hrs$timepoint != "T48"]

vst_filt <- vst[, vst$timepoint %in% c("T0", "T4", "T24")]

vst_48hrs <- vst_48hrs[rownames(vst_48hrs) %in% rownames(vst_filt), ]
vst_filt <- vst_filt[rownames(vst_filt) %in% rownames(vst_48hrs), ]

vst_48hrs_8dpp <- vst_48hrs[, vst_48hrs$age == "8dpp"]
vst_48hrs_16dpp <- vst_48hrs[, vst_48hrs$age == "16dpp"]


vst_filt_8dpp <- vst_filt[, vst_filt$age == "8dpp" & vst_filt$treatment == "Inoc"]
vst_filt_16dpp <- vst_filt[, vst_filt$age == "16dpp" & vst_filt$treatment == "Inoc"]

both_8dpp <- cbind(assay(vst_filt_8dpp), assay(vst_48hrs_8dpp))
both_16dpp <- cbind(assay(vst_filt_16dpp), assay(vst_48hrs_16dpp))
bothAges <- cbind(both_8dpp, both_16dpp)

pcaBoth <- prcomp(t(bothAges))

percentVar <- pcaBoth$sdev^2 / sum( pcaBoth$sdev^2 )

as.data.frame(prcomp(t(bothAges))$x) %>%
  mutate(sample = rownames(.),
         timepoint = fct_relevel(str_extract(sample, pattern = "T[0-9]*"), "T24", after = Inf),
         exp = c(rep(c("1", "2"), each = 9), rep(c("1", "2"), each = 9)),
         age = rep(c("8dpp", "16dpp"), each = 18)
           ) %>%
    mutate(Age = fct_relevel(age, "8dpp")) %>%
  ggplot(aes(x = PC1, y = PC2, color = timepoint, shape = Age)) +
    geom_point(size = 4, stroke = 2) +
    geom_point(aes(fill = timepoint, shape = Age, alpha = exp), size = 5, stroke = 0) +
    cowplot::theme_cowplot(font_size = 14) +
    scale_shape_manual(values = c(21, 24, 21, 24)) +
    scale_alpha_manual(values = c(0.05, 1, 0.05, 1)) +
    guides(alpha = guide_legend(title = "Experiment",
                                override.aes = list(shape = c(22, 15), stroke = 2, alpha = 1))) +
    xlab(paste0("PC1: ",round(percentVar[1] * 100),"% variance")) +
    ylab(paste0("PC2: ",round(percentVar[2] * 100),"% variance")) +
    theme(
        strip.text = element_text(
        colour = "grey10",
        size = rel(0.8),
        margin = margin(0.5 * 7, 0.5 * 7, 0.5 * 7, 0.5 * 7)
        )) +
```

```
    # lemon::facet_rep_wrap(~ Age, scales = "free", ncol = 1) +
    cowplot::panel_border()
```

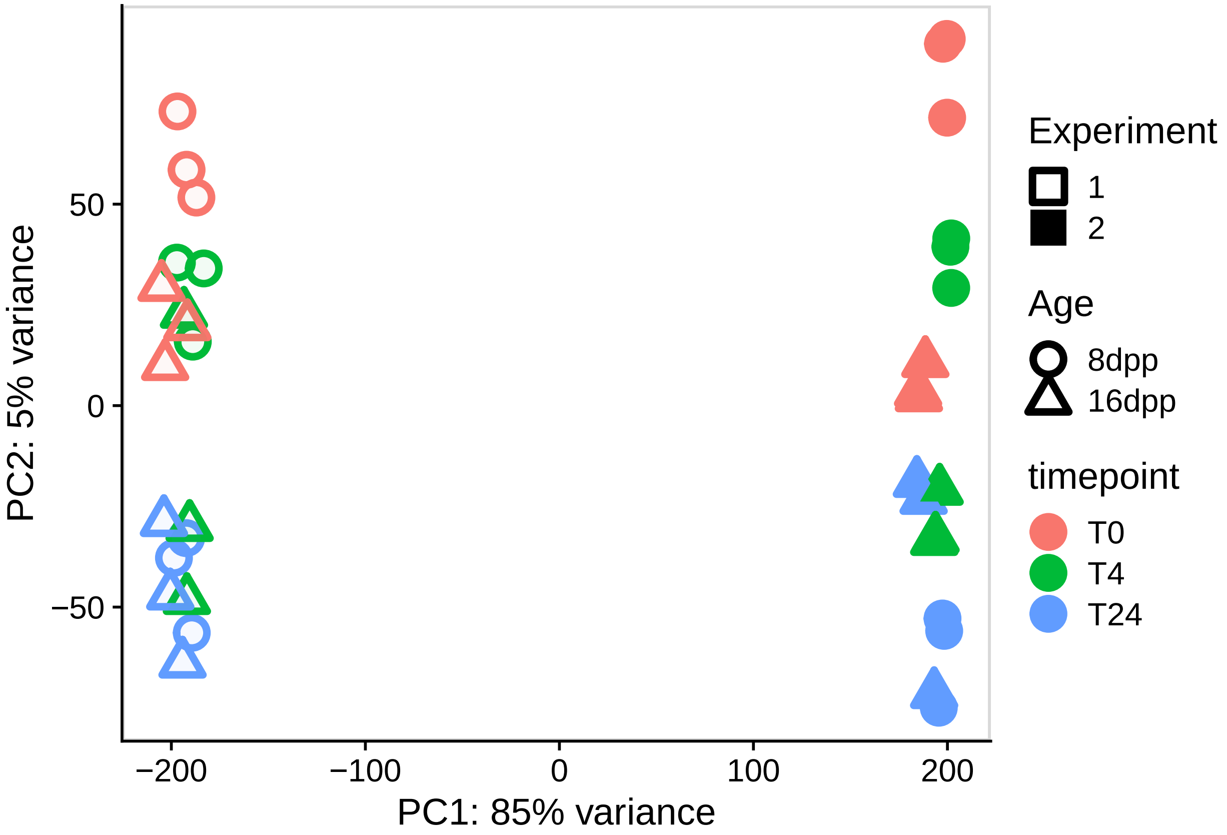

Correct for batch effects:

```
bothAges_BatchEff <- limma::removeBatchEffect(x = bothAges,
                                               batch = c(rep(c("1", "2"), each = 9),
                                                         rep(c("1", "2"), each = 9))
                                               )

pcaBoth_BatchEff <- prcomp(t(bothAges_BatchEff))

percentVar <- pcaBoth_BatchEff$sdev^2 / sum( pcaBoth_BatchEff$sdev^2 )

as.data.frame(prcomp(t(bothAges_BatchEff))$x) %>%
  mutate(sample = rownames(.),
         timepoint = fct_relevel(str_extract(sample, pattern = "T[0-9]*"), "T24", after = Inf),
         exp = c(rep(c("1", "2"), each = 9), rep(c("1", "2"), each = 9)),
         age = rep(c("8dpp", "16dpp"), each = 18)
           ) %>%
    mutate(Age = fct_relevel(age, "8dpp")) %>%
  ggplot(aes(x = PC1, y = PC2, color = timepoint, shape = Age)) +
    geom_point(size = 4, stroke = 2) +
    geom_point(aes(fill = timepoint, shape = Age, alpha = exp), size = 5, stroke = 0) +
    cowplot::theme_cowplot(font_size = 14) +
    scale_shape_manual(values = c(21, 24, 21, 24)) +
```

```
    scale_alpha_manual(values = c(0.05, 1, 0.05, 1)) +
    guides(alpha = guide_legend(title = "Experiment",
                                override.aes = list(shape = c(22, 15), stroke = 2, alpha = 1))) +
    xlab(paste0("PC1: ",round(percentVar[1] * 100),"% variance")) +
    ylab(paste0("PC2: ",round(percentVar[2] * 100),"% variance")) +
    theme(
        strip.text = element_text(
        colour = "grey10",
        size = rel(0.8),
        margin = margin(0.5 * 7, 0.5 * 7, 0.5 * 7, 0.5 * 7)
        )) +
    # lemon::facet_rep_wrap(~ Age, scales = "free", ncol = 1) +
    cowplot::panel_border()
```

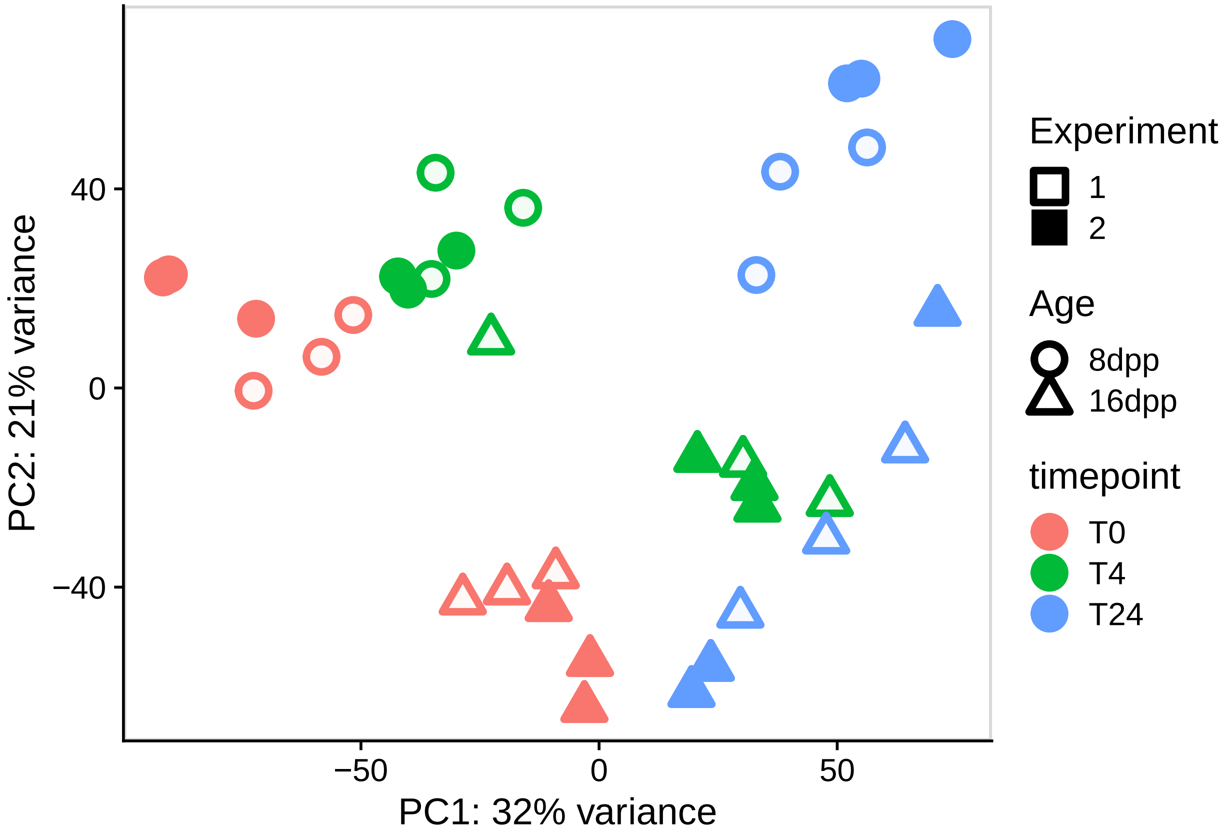

Weighted gene co-expression analysis

We then separate the samples in to the relevant networks

```
select_union <- rownames(vst) ### ALL GENES ###

topVar_16dpp <- assay(vst)[select_union, vst$treatment == "Inoc" & vst$age == "16dpp"]

topVar_8dpp <- assay(vst)[select_union, vst$treatment == "Inoc" & vst$age == "8dpp"]


infection8dpp <- t(topVar_8dpp)
```

```
infection16dpp <- t(topVar_16dpp)

control16dpp <- t(assay(vst)[, vst$treatment == "Cont" & vst$age == "16dpp"])
control8dpp <- t(assay(vst)[, vst$treatment == "Cont" & vst$age == "8dpp"])
```

Checking and filtering genes and samples based on WGCNA guidlines

```
gsg <- goodSamplesGenes(infection16dpp, verbose = 3)
```

```
##  Flagging genes and samples with too many missing values...
##   ..step 1
##   ..Excluding 1 genes from the calculation due to too many missing samples or zero variance.
##   ..step 2
```

```
gsg$allOK
```

```
## [1] FALSE
```

```
if (!gsg$allOK)
{
# Optionally, print the gene and sample names that were removed:
if (sum(!gsg$goodGenes) > 0)
printFlush(paste("Removing genes:",
                 paste(colnames(infection16dpp)[!gsg$goodGenes], collapse = ", ")))

if (sum(!gsg$goodSamples) > 0)
printFlush(paste("Removing samples:",
                 paste(rownames(infection16dpp)[!gsg$goodSamples], collapse = ", ")))

# Remove the offending genes and samples from the data:
infection16dpp <- infection16dpp[gsg$goodSamples, gsg$goodGenes]
}
```

```
## Removing genes: Csa3G141830
```

```
gsg <- goodSamplesGenes(infection8dpp, verbose = 3)
```

```
##  Flagging genes and samples with too many missing values...
##   ..step 1
```

```
gsg$allOK
```

```
## [1] TRUE
```

```
if (!gsg$allOK)
{
# Optionally, print the gene and sample names that were removed:
if (sum(!gsg$goodGenes) > 0)
printFlush(paste("Removing genes:",
                 paste(colnames(infection8dpp)[!gsg$goodGenes], collapse = ", ")))

if (sum(!gsg$goodSamples) > 0)
printFlush(paste("Removing samples:",
                 paste(rownames(infection8dpp)[!gsg$goodSamples], collapse = ", ")))

# Remove the offending genes and samples from the data:
infection8dpp <- infection8dpp[gsg$goodSamples, gsg$goodGenes]
}
```

```r
if (!identical(colnames(infection16dpp), colnames(infection8dpp))) {
  message("Genes not identical")
  message(paste("Removing genes from 16dpp:",
                paste(colnames(infection16dpp)[!colnames(infection16dpp) %in% colnames(infection8dpp)],
                      collapse = ", ")))
  infection16dpp <- infection16dpp[, colnames(infection16dpp) %in% colnames(infection8dpp)]
  message(paste("Removing genes from 8dpp:",
                paste(colnames(infection16dpp)[!colnames(infection8dpp) %in% colnames(infection16dpp)],
                      collapse = ", ")))
  infection8dpp <- infection8dpp[, colnames(infection8dpp) %in% colnames(infection16dpp)]
} else {
message("Treatments have same genes")
}
```

Plotting sample dendrograms to identify if there are outlier samples that act as outgroups to the whole set.

```r
## Plot the sample dendros
sampleTree16 <- hclust(dist(infection16dpp), method = "average")
sampleTree8 <- hclust(dist(infection8dpp), method = "average")
```

Looks like all are good to include in the analysis

```r
par(cex = 0.6);
par(mar = c(0,4,2,0))
plot(sampleTree16, main = "Sample clustering to detect outliers 16dpp", sub="", xlab="", cex.lab = 1.5,
     cex.axis = 1.5, cex.main = 2)
```

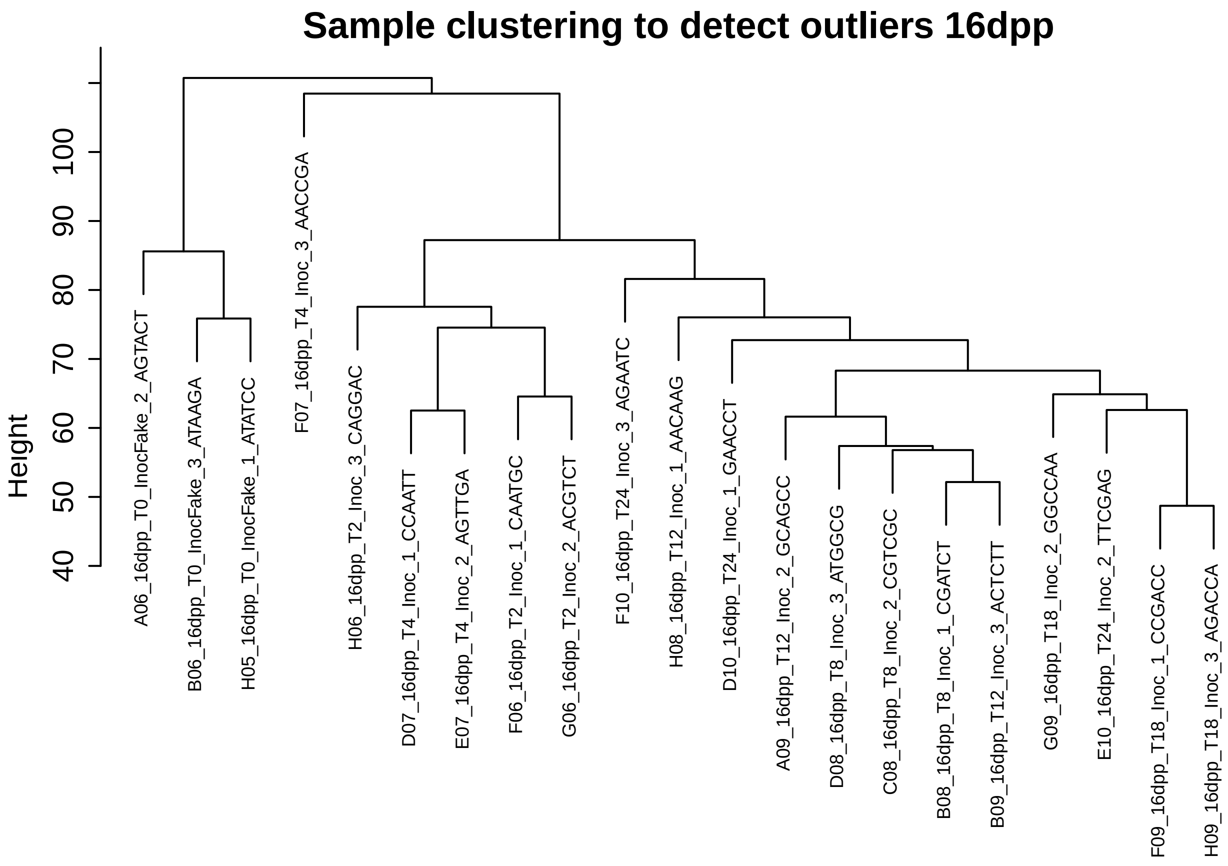

```r
plot(sampleTree8, main = "Sample clustering to detect outliers 8dpp", sub="", xlab="", cex.lab = 1.5,
     cex.axis = 1.5, cex.main = 2)
```

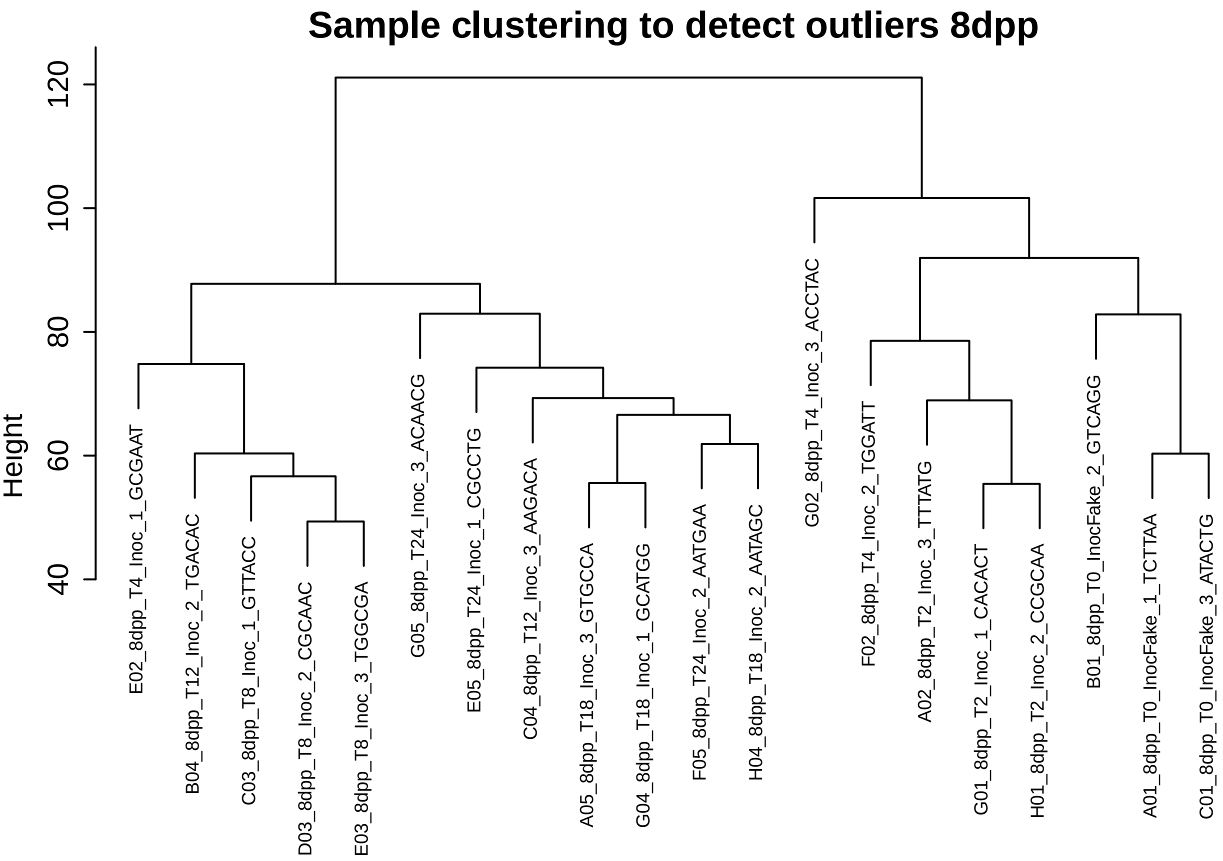

Calculate scale free topology to try to choose the best power for adjacency calculation We are using signed networks and the bicor correlation function.

```r
# Choose a set of soft-thresholding powers
powers <- c(c(1:10), seq(from = 12, to=30, by=2))
# Call the network topology analysis function
sft16 <-
  pickSoftThreshold(
    infection16dpp,
    powerVector = powers,
    verbose = 0,
    networkType = "signed",
    corFnc = "bicor",
    corOptions = "maxPOutliers = 0.1"
  )

sft8 <-
  pickSoftThreshold(
  infection8dpp,
  powerVector = powers,
  verbose = 0,
  networkType = "signed",
  corFnc = "bicor",
```

```
  corOptions = "maxPOutliers = 0.1"
  )

bind_rows("16dpp" = sft16$fitIndices, "8dpp" = sft8$fitIndices, .id = "network") %>%
ggplot() +
  geom_text(aes(x = Power, y = -sign(slope) * SFT.R.sq, label = Power, color = network)) +
  geom_abline(intercept = 0.8, slope = 0, color = "red") +
  ylab("Scale Free Topology Model Fit,signed R^2")
```

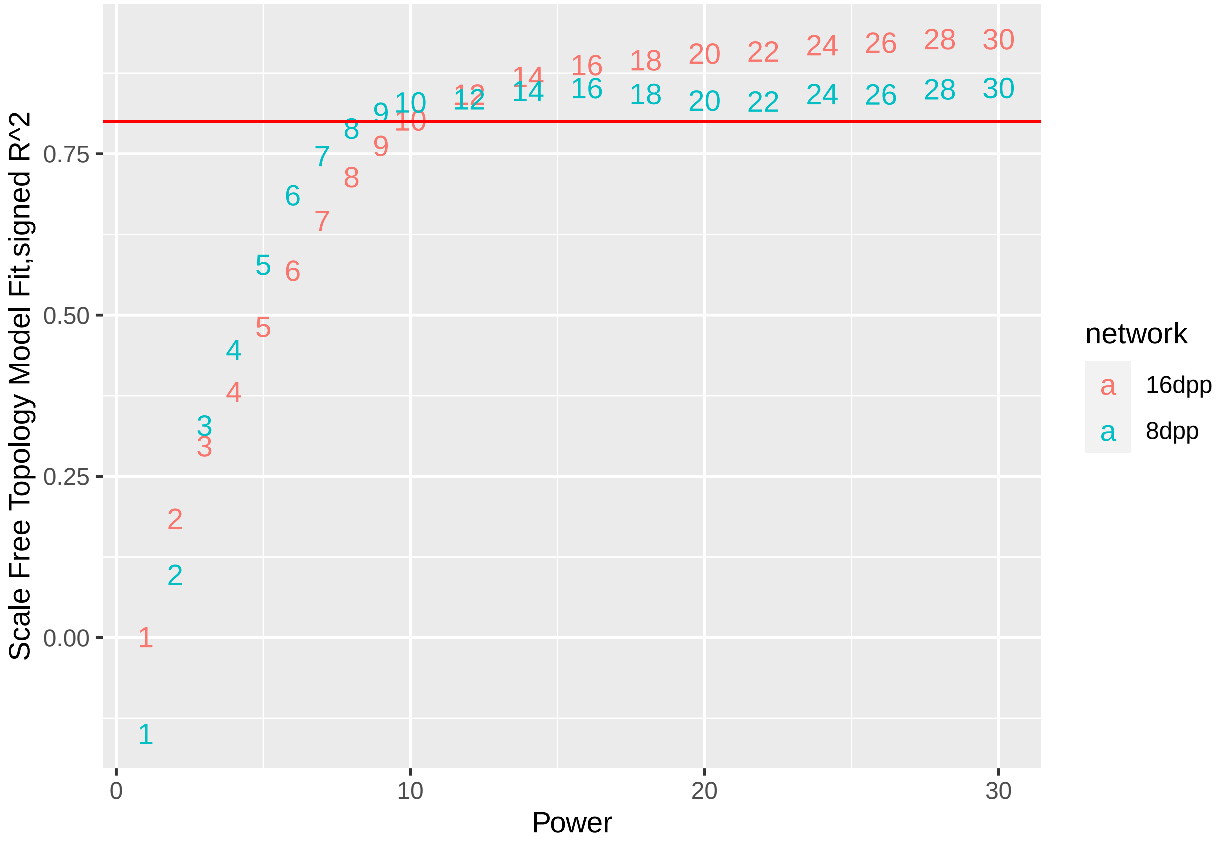

We choose 12 as a power because the both networks reach a scale free topology R^2 fit of greater than 0.8

```
# calculate adjacency matrix using bicor
softPower <- 12;
adjacency16 <-
  adjacency(
  infection16dpp,
  power = softPower,
  type = "signed",
  corFnc = "bicor",
  corOptions = "maxPOutliers = 0.1"
  )

adjacency8 <-
  adjacency(
  infection8dpp,
  power = softPower,
  type = "signed",
```

```
  corFnc = "bicor",
  corOptions = "maxPOutliers = 0.1"
  )
```

We then take the adjacency matrix and calculate the Topological Overlap Matrix

```
# TOMType doesn't matter in signed networks
dissTOM16 <- 1 - TOMsimilarity(adjacency16, TOMType = "unsigned")
```

```
## ..connectivity..
## ..matrix multiplication (system BLAS)..
## ..normalization..
## ..done.
```

```
dissTOM8 <- 1 - TOMsimilarity(adjacency8, TOMType = "unsigned")
```

```
## ..connectivity..
## ..matrix multiplication (system BLAS)..
## ..normalization..
## ..done.
```

Plot the gene dendrograms based on their dissimilarty

```
geneTree16 <- hclust(as.dist(dissTOM16), method = "average");
geneTree8 <- hclust(as.dist(dissTOM8), method = "average");

plot(geneTree16, xlab = "", sub = "", main = "Gene clustering on TOM-based dissimilarity 16dpp",
     labels = FALSE, hang = 0.04);
```

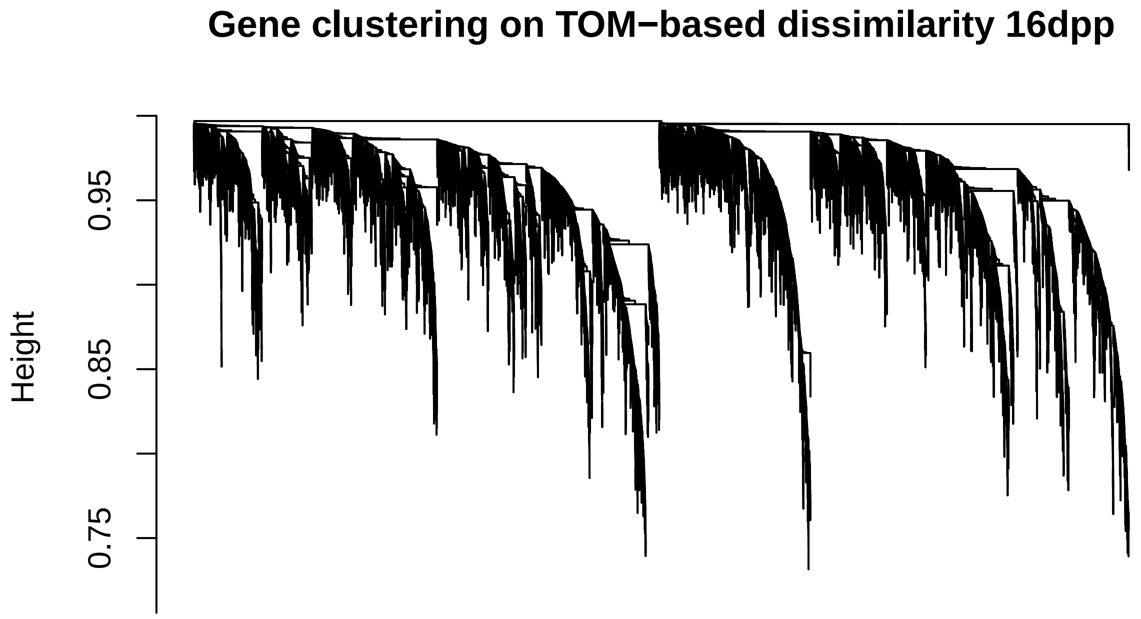

```r
plot(geneTree8, xlab = "", sub = "", main = "Gene clustering on TOM-based dissimilarity 8dpp",
     labels = FALSE, hang = 0.04);
```

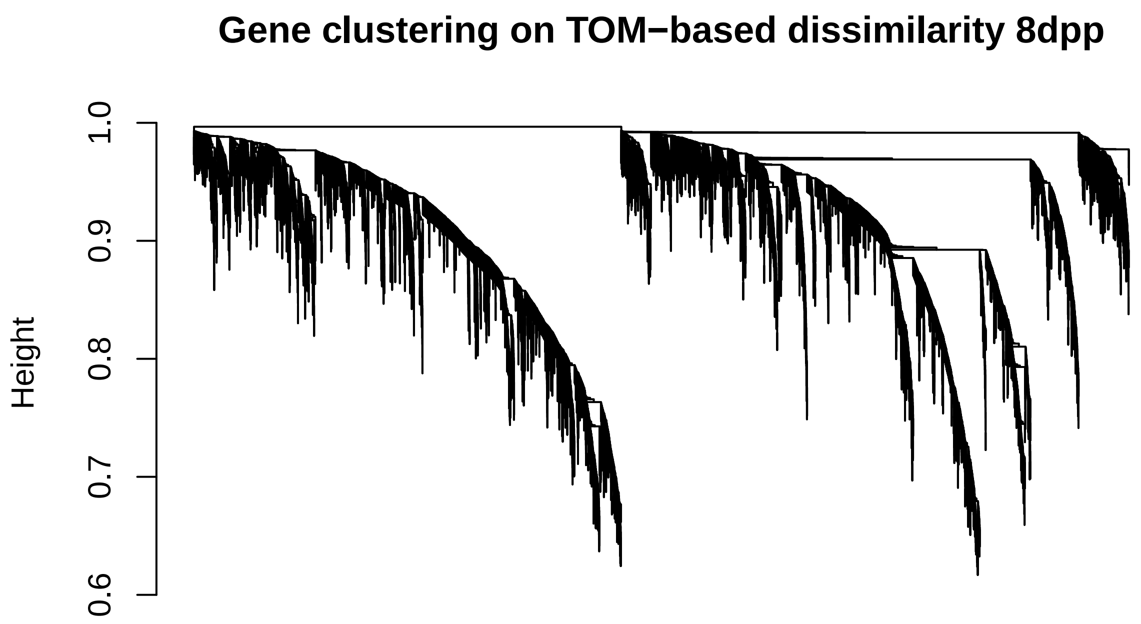

We then use the dynamic tree cut algorithm to identify first pass modules. These will then be merged.

```r
minModuleSize <- 30;
# Module identification using dynamic tree cut:
dynamicMods16 <- cutreeDynamic(dendro = geneTree16,
                              distM = dissTOM16,
                              deepSplit = 2,
                              pamRespectsDendro = FALSE,
                              minClusterSize = minModuleSize);
```

```
##  ..cutHeight not given, setting it to 0.995  ===>  99% of the (truncated) height range in dendro.
##  ..done.
```

```r
dynamicMods8 <- cutreeDynamic(dendro = geneTree8,
                               distM = dissTOM8,
                               deepSplit = 2,
                               pamRespectsDendro = FALSE,
                               minClusterSize = minModuleSize);
```

```
##  ..cutHeight not given, setting it to 0.994  ===>  99% of the (truncated) height range in dendro.
##  ..done.
```

```r
# Convert numeric lables into colors
dynamicColors16 <- labels2colors(dynamicMods16)
table(dynamicColors16)
```

```
## dynamicColors16
##   antiquewhite4         bisque4           black            blue           brown
##              57              94             614            1444             797
##          brown4          coral1          coral2            cyan       darkgreen
##              94              59              55             334             208
##        darkgrey     darkmagenta  darkolivegreen      darkorange     darkorange2
##             181             133             134             170              96
##         darkred    darkseagreen4   darkslateblue   darkturquoise     floralwhite
##             208              63              93             182              98
##           green     greenyellow          grey60       honeydew1           ivory
##             671             404             262              72             102
##  lavenderblush3       lightcyan      lightcyan1      lightgreen      lightpink4
##              73             270             103             241              75
## lightsteelblue1     lightyellow         magenta          maroon     mediumorchid
##             104             227             527              75              54
##   mediumpurple3    midnightblue    navajowhite2          orange      orangered4
##             104             276              75             178             111
##   paleturquoise palevioletred3            pink           plum1           plum2
##             152              78             570             117              92
##          purple             red       royalblue     saddlebrown          salmon
##             451             653             227             157             351
##         salmon4         sienna3         skyblue        skyblue1        skyblue2
##              84             128             162              46              53
##        skyblue3       steelblue             tan        thistle1        thistle2
##             119             154             370              91              91
##       turquoise          violet           white          yellow         yellow4
##            1746             136             163             752              52
##     yellowgreen
##             126
```

```
dynamicColors8 <- labels2colors(dynamicMods8)
table(dynamicColors8)
```

```
## dynamicColors8
##         black          blue         brown          cyan     darkgreen
##           633          2381          2152           219           137
##      darkgrey    darkorange       darkred darkturquoise         green
##           119            96           150           131          1147
##   greenyellow        grey60     lightcyan    lightgreen   lightyellow
##           383           185           210           182           159
##       magenta  midnightblue        orange paleturquoise          pink
##           451           218           115            70           542
##        purple           red     royalblue   saddlebrown        salmon
##           445           890           151            86           233
##       skyblue     steelblue           tan     turquoise        violet
##            94            86           364          2608            48
##         white        yellow
##            94          1460
```

ID first module eigengenes and then cluster based on dissimilarity

```
## calculate eigengenes

MEList16 <- moduleEigengenes(infection16dpp, colors = dynamicColors16)
MEs16 <- MEList16$eigengenes
```

```
MEList8 <- moduleEigengenes(infection8dpp, colors = dynamicColors8)
MEs8 <- MEList8$eigengenes

# Calculate dissimilarity of module eigengenes
MEDiss16 <- 1-cor(MEs16)
MEDiss8 <- 1-cor(MEs8)
```

Let's merge at three different thresholds

```
# Cluster module eigengenes
METree16 <- hclust(as.dist(MEDiss16), method = "average");
# Plot the result

par(cex = 0.6);
par(mar = c(0,4,2,0))
plot(METree16, main = "Clustering of module eigengenes",
     xlab = "", sub = "")
abline(h=0.15, col = "red")
abline(h=0.20, col = "blue")
abline(h=0.25, col = "green")
abline(h=0.30, col = "purple")
```

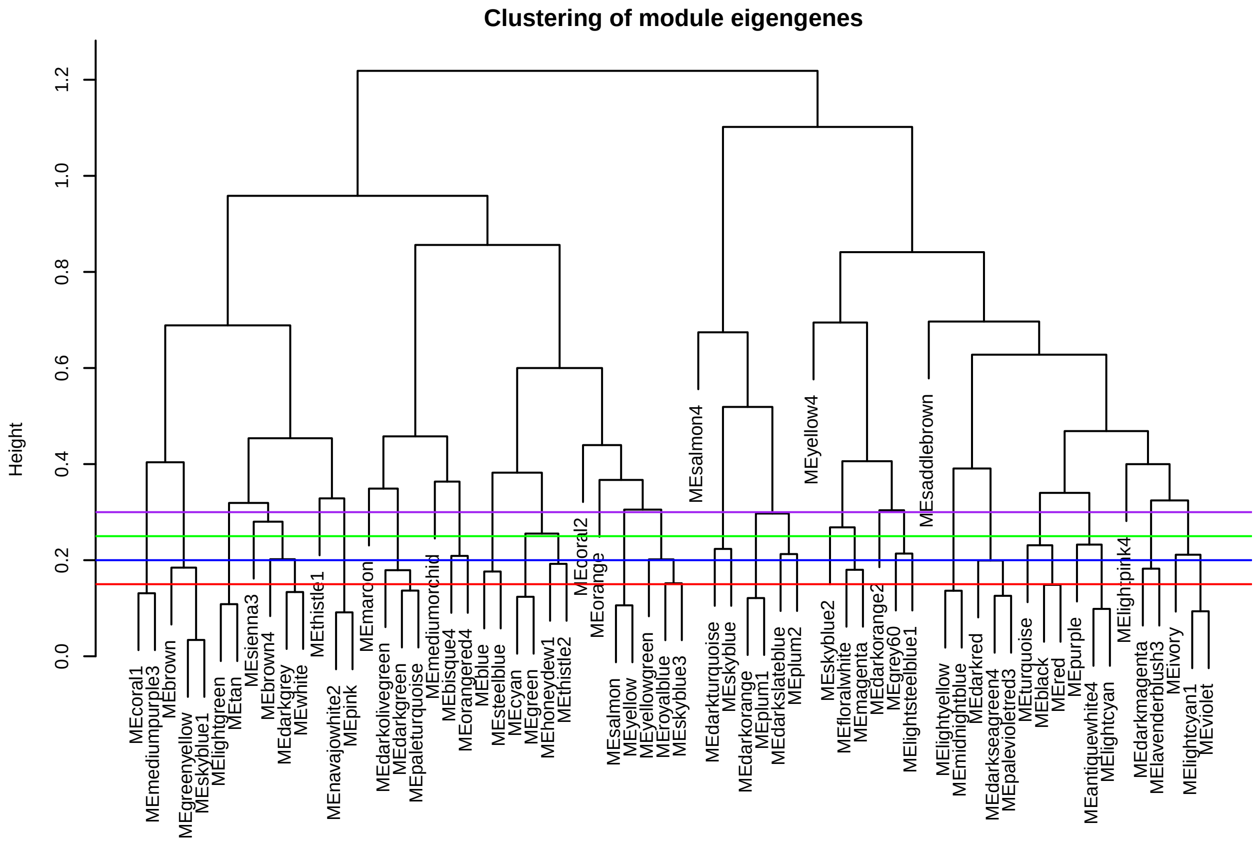


```
## find a good merge

MEDissThres <- 0.15
# Call an automatic merging function
merge16.15 <-
```

```r
mergeCloseModules(infection16dpp,
dynamicColors16,
cutHeight = MEDissThres,
verbose = 0)
# The merged module colors
merged16Colors.15 <- merge16.15$colors

# Eigengenes of the new merged modules:
merged16MEs.15 <- merge16.15$newMEs


MEDissThres <- 0.20
# Call an automatic merging function
merge16.20 <-
mergeCloseModules(infection16dpp,
dynamicColors16,
cutHeight = MEDissThres,
verbose = 0)
# The merged module colors
merged16Colors.20 <- merge16.20$colors

# Eigengenes of the new merged modules:
merged16MEs.20 <- merge16.20$newMEs

MEDissThres <- 0.25
# Call an automatic merging function
merge16.25 <-
mergeCloseModules(infection16dpp,
dynamicColors16,
cutHeight = MEDissThres,
verbose = 0)
# The merged module colors
merged16Colors.25 <- merge16.25$colors

# Eigengenes of the new merged modules:
merged16MEs.25 <- merge16.25$newMEs

MEDissThres <- 0.30
# Call an automatic merging function
merge16.30 <-
mergeCloseModules(infection16dpp,
dynamicColors16,
cutHeight = MEDissThres,
verbose = 0)
# The merged module colors
merged16Colors.30 <- merge16.30$colors

# Eigengenes of the new merged modules:
merged16MEs.30 <- merge16.30$newMEs
```

And the same for the 8dpp network

```r
# Cluster module eigengenes
METree8 <- hclust(as.dist(MEDiss8), method = "average");
```

```r
# Plot the result

par(cex = 0.6);
par(mar = c(0,4,2,0))
plot(METree8, main = "Clustering of module eigengenes",
     xlab = "", sub = "")
abline(h=0.15, col = "red")
abline(h=0.20, col = "blue")
abline(h=0.25, col = "green")
abline(h=0.30, col = "purple")
```

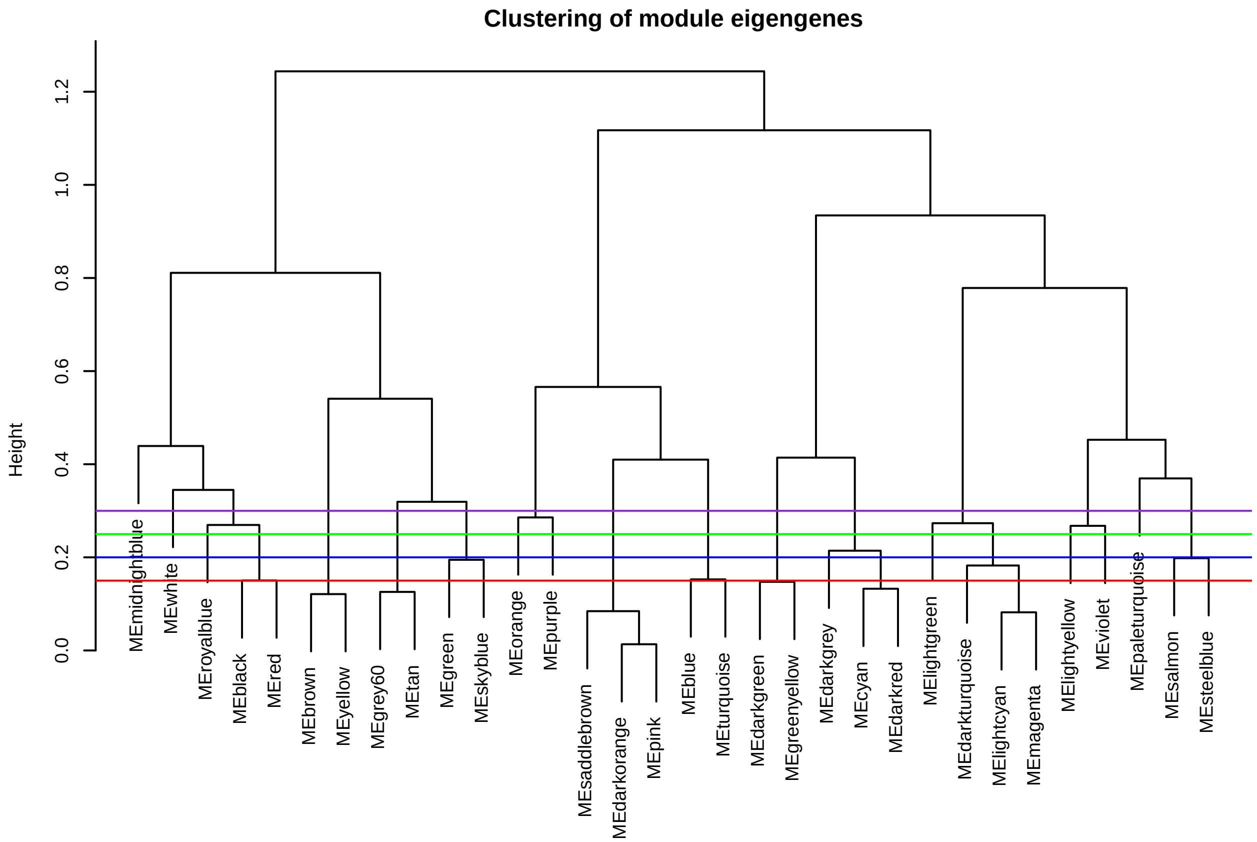


```r
## find a good merge

MEDissThres <- 0.15
# Call an automatic merging function
merge8.15 <-
mergeCloseModules(infection8dpp,
dynamicColors8,
cutHeight = MEDissThres,
verbose = 0)
# The merged module colors
merged8Colors.15 <- merge8.15$colors

# Eigengenes of the new merged modules:
merged8MEs.15 <- merge8.15$newMEs
```

```
MEDissThres <- 0.20
# Call an automatic merging function
merge8.20 <-
mergeCloseModules(infection8dpp,
dynamicColors8,
cutHeight = MEDissThres,
verbose = 0)
# The merged module colors
merged8Colors.20 <- merge8.20$colors

# Eigengenes of the new merged modules:
merged8MEs.20 <- merge8.20$newMEs

MEDissThres <- 0.25
# Call an automatic merging function
merge8.25 <-
mergeCloseModules(infection8dpp,
dynamicColors8,
cutHeight = MEDissThres,
verbose = 0)
# The merged module colors
merged8Colors.25 <- merge8.25$colors

# Eigengenes of the new merged modules:
merged8MEs.25 <- merge8.25$newMEs

MEDissThres <- 0.30
# Call an automatic merging function
merge8.30 <-
mergeCloseModules(infection8dpp,
dynamicColors8,
cutHeight = MEDissThres,
verbose = 0)
# The merged module colors
merged8Colors.30 <- merge8.30$colors

# Eigengenes of the new merged modules:
merged8MEs.30 <- merge8.30$newMEs
```

Ok lets compare the merged modules to the orginal dynamic tree cut and the gene dendrogram

```
plotDendroAndColors(geneTree16, cbind(dynamicColors16, merged16Colors.15, merged16Colors.20, merged16Col
                    c("Dynamic Tree Cut", "Merged 0.15", "Merged 0.20", "Merged 0.25", "Merged 0.30"),
                    dendroLabels = FALSE, hang = 0.03,
                    addGuide = TRUE, guideHang = 0.05,
                    main = "Cluster dendrogram - 16dpp")
```

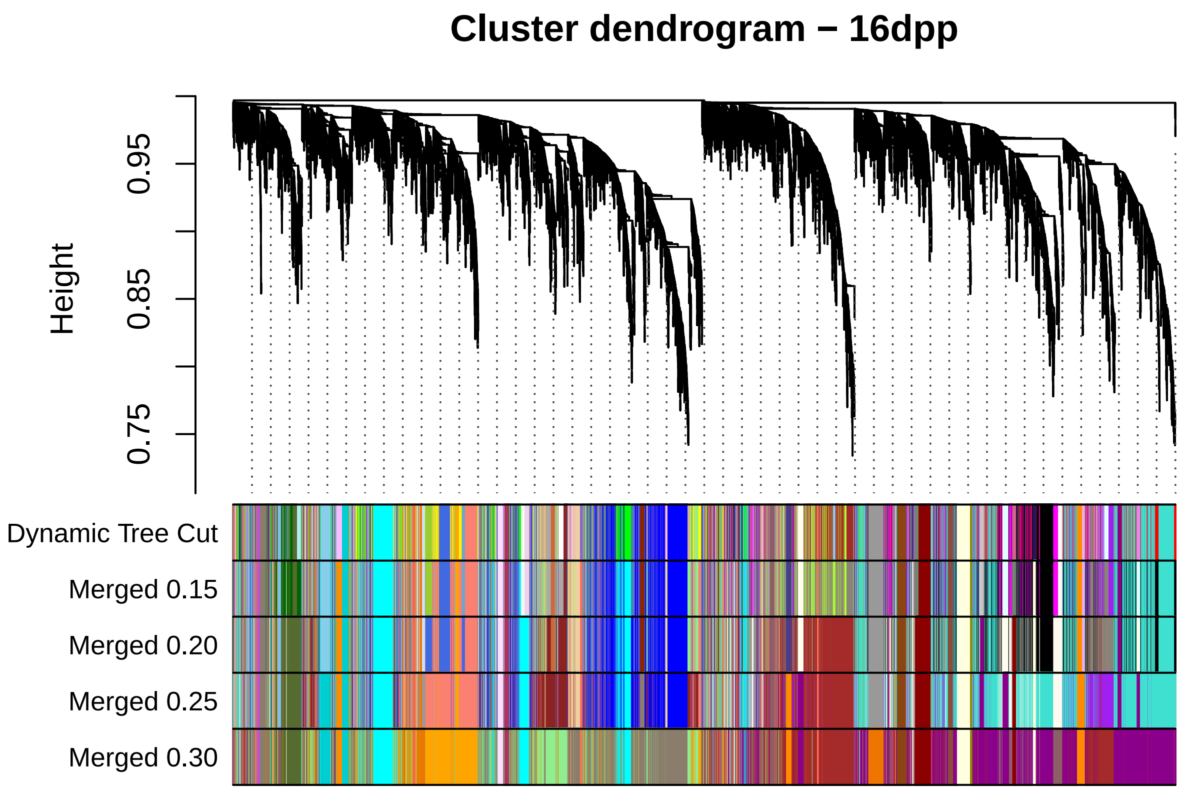


```
plotDendroAndColors(geneTree8, cbind(dynamicColors8, merged8Colors.15, merged8Colors.20, merged8Colors.
                    c("Dynamic Tree Cut", "Merged 0.15", "Merged 0.20", "Merged 0.25", "Merged 0.30"),
                    dendroLabels = FALSE, hang = 0.03,
                    addGuide = TRUE, guideHang = 0.05,
                    main = "Cluster dendrogram - 8dpp")
```

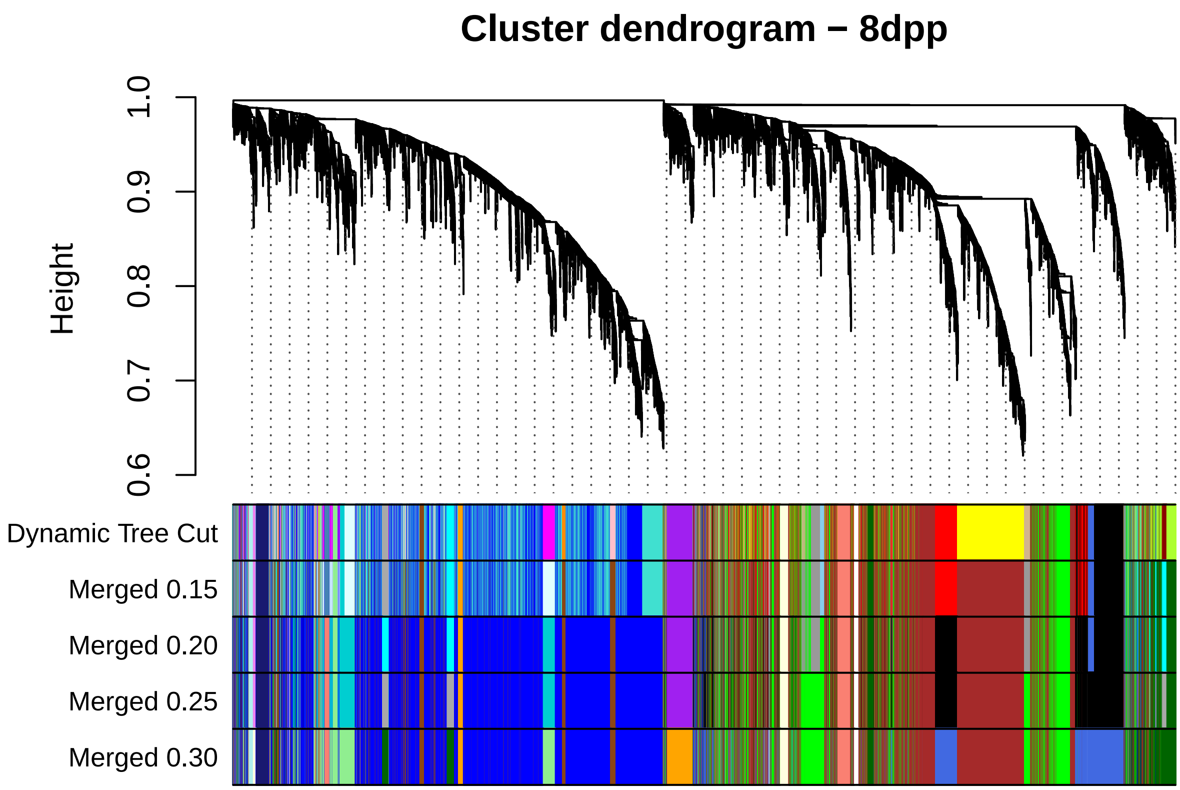

We choose the 0.25 merge level

```r
# Rename to selected merged moduleColors
moduleColors16 <- merged16Colors.25
moduleColors8 <- merged8Colors.25

# Construct numerical labels corresponding to the colors
finalColors16 <- names(sort(table(moduleColors16), decreasing = TRUE))
finalColors16 <- finalColors16[!finalColors16 %in% "grey"]

finalColors8 <- names(sort(table(moduleColors8), decreasing = TRUE))
finalColors8 <- finalColors8[!finalColors8 %in% "grey"]

colorOrder <- c("grey", finalColors16)
moduleLabels16 <- match(moduleColors16, colorOrder) - 1

colorOrder <- c("grey", finalColors8)
moduleLabels8 <- match(moduleColors8, colorOrder) - 1

table(moduleLabels16)
```

```
## moduleLabels16
##    1    2    3    4    5    6    7    8    9   10   11   12   13   14   15   16
## 3013 1598 1575 1247 1177 1168  778  645  625  547  503  494  472  366  349  344
##   17   18   19   20   21   22   23   24   25   26   27   28   29
##  205  178  163  157   96   91   84   75   75   55   54   53   52
```

```r
table(moduleLabels8)
```

```
## moduleLabels8
##    1    2    3    4    5    6    7    8    9   10   11   12   13   14   15   16
## 4989 3612 1790 1674  792  724  520  488  445  319  218  182  159  115   94   70
##   17
##   48
```

```r
#overlapTable(moduleLabels16, moduleLabels8)
```

Recolor with prettier colors

```r
qual_col_pals <- brewer.pal.info[brewer.pal.info$category == 'qual',]

col_vector <- unique(unlist(mapply(brewer.pal, qual_col_pals$maxcolors, rownames(qual_col_pals))))

# recolor modules so there are no overlaps between the network colors
RNGkind(sample.kind = "Rounding")
set.seed(7)
customColorOrder16 <-
c(
"grey",
sample(col_vector,
       size = length(unique(moduleLabels16[!moduleLabels16 %in% 0])),
       replace = FALSE
       )
)
moduleColors16.custom <- customColorOrder16[moduleLabels16 + 1]

set.seed(9)
customColorOrder8 <-
c(
"grey",
sample(col_vector[!col_vector %in% unique(moduleColors16.custom)],
       size = length(unique(moduleLabels8[!moduleLabels8 %in% 0])),
       replace = FALSE)
)
moduleColors8.custom <- customColorOrder8[moduleLabels8 + 1]
```

Selected the new MEs after merging

```r
MEs16Lables <- moduleEigengenes(infection16dpp, colors = moduleLabels16)$eigengenes
MEs8Lables <- moduleEigengenes(infection8dpp, colors = moduleLabels8)$eigengenes

MEs16Colors <- moduleEigengenes(infection16dpp, colors = moduleColors16.custom)$eigengenes
MEs8Colors <- moduleEigengenes(infection8dpp, colors = moduleColors8.custom)$eigengenes
```

Plot the final gene trees and modules

```r
plotDendroAndColors(geneTree16,
                    cbind(moduleColors16.custom),
                    "Resistant\nmodules\n(16dpp)",
                    dendroLabels = FALSE, hang = 0.03,
                    addGuide = TRUE, guideHang = 0.05,
                    main = "Resistant-aged (16dpp) fruit\ngene dendrogram and module colors")
```

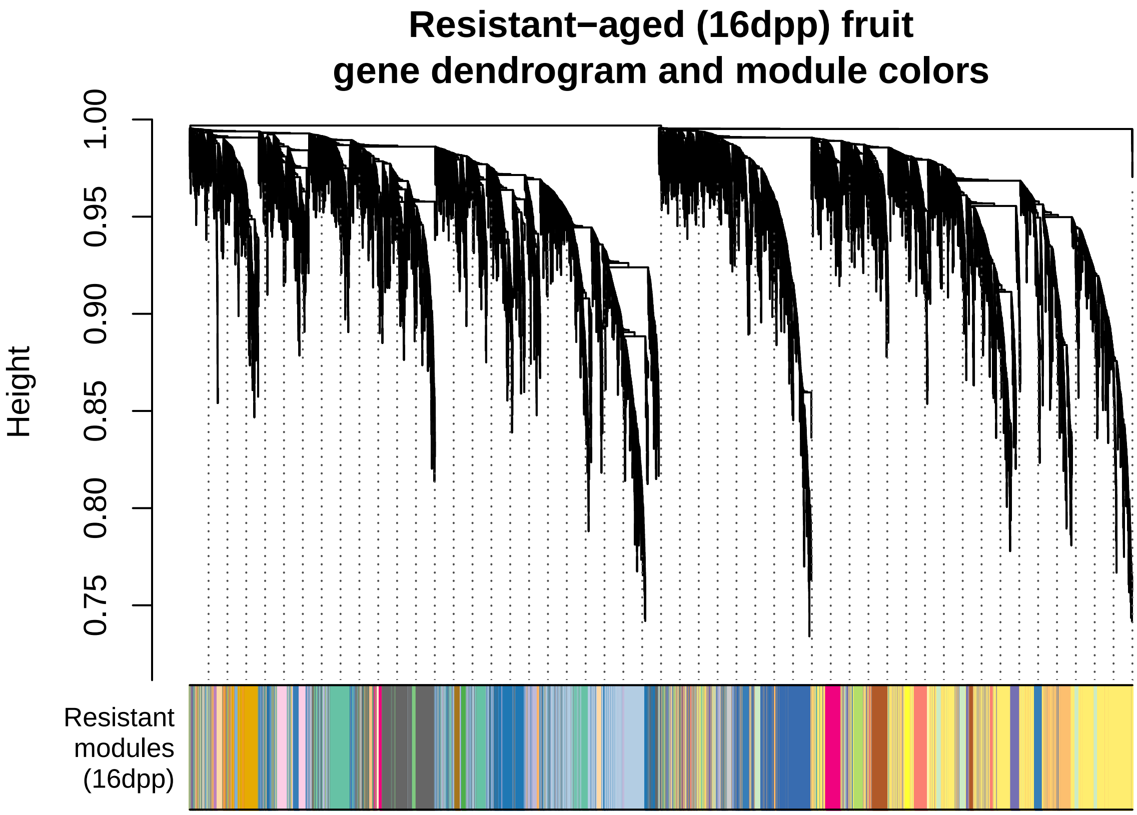


```r
plotDendroAndColors(geneTree8,
                    cbind(moduleColors8.custom),
                    "Susceptible\nmodules\n(8dpp)",
                    dendroLabels = FALSE, hang = 0.03,
                    addGuide = TRUE, guideHang = 0.05,
                    main = "Susceptible-aged (8dpp) fruit\ngene dendrogram and module colors")
```

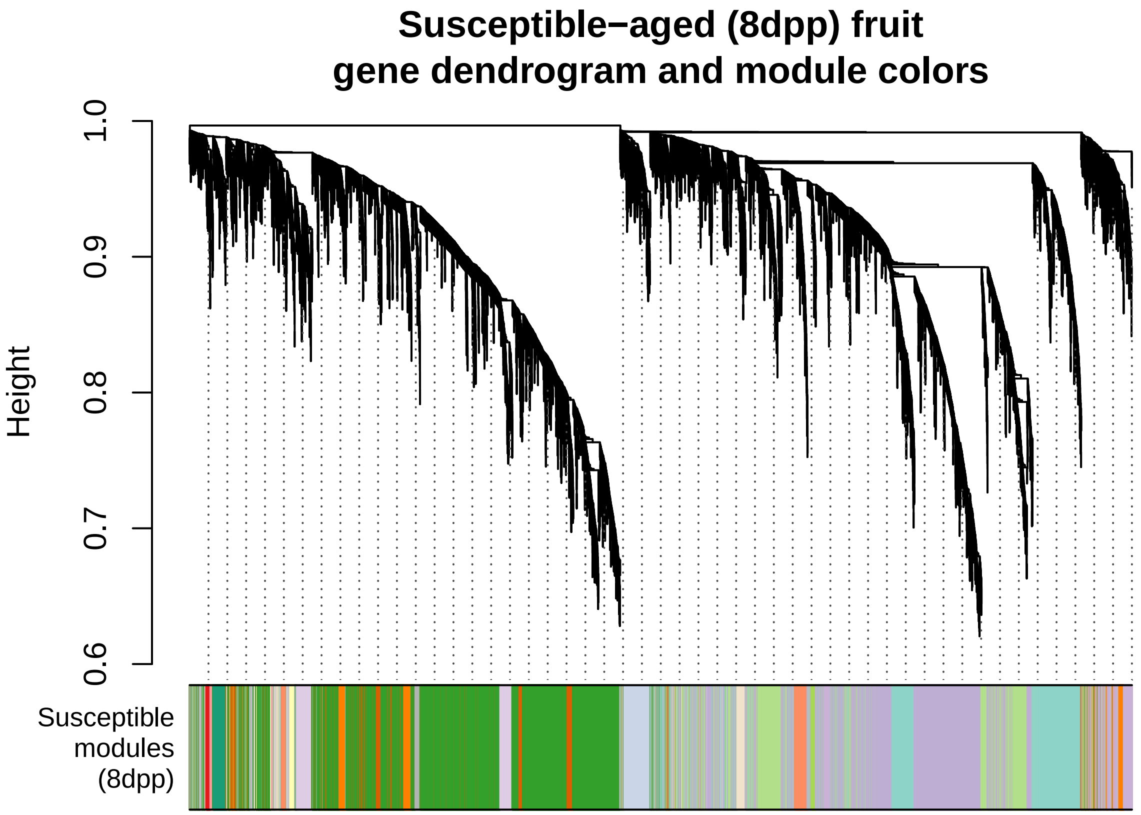


```
#
# plotDendroAndColors(geneTree8,
#                     cbind(moduleColors8.custom),
#                     "Susceptible\nmodules\n(8dpp)",
#                     dendroLabels = FALSE, hang = 0.03,
#                     addGuide = TRUE, guideHang = 0.05,
#                     main = "Susceptible-aged (8dpp) fruit\ngene dendrogram and module colors")

pdf(file = "fig6A.pdf", width = 6.5, height = 4.5)
    plotDendroAndColors(geneTree8,
                    cbind(moduleColors8.custom),
                    "Susceptible\nmodules\n(8dpp)",
                    dendroLabels = FALSE, hang = 0.03,
                    addGuide = TRUE, guideHang = 0.05,
                    main = "Susceptible-aged (8dpp) fruit\ngene dendrogram and module colors")
dev.off()
```

```
## pdf
##   2
```

```
pdf(file = "fig6B.pdf", width = 6.5, height = 4.5)
    plotDendroAndColors(geneTree16,
                    cbind(moduleColors16.custom),
                    "Resistant\nmodules\n(16dpp)",
                    dendroLabels = FALSE, hang = 0.03,
                    addGuide = TRUE, guideHang = 0.05,
                    main = "Resistant-aged (16dpp) fruit\ngene dendrogram and module colors")
```

```
dev.off()
```

```
## pdf
##   2
```

Let's examine the module preservation. This step is really long computationally and extremely verbose so this is not run here to save time and space in the document.

```
# Number of data sets that we work with
nSets <- 2;
# Object that will contain the expression data
multiExpr <- list();
multiExpr[[1]] = list(data = infection16dpp)
multiExpr[[2]] = list(data = infection8dpp)

# Names for the two sets
setLabels <- c("16dpp", "8dpp");
# Important: components of multiExpr must carry identificating names
names(multiExpr) <- setLabels
# Display the dimensions of the expression data (if you are confused by this construct, ignore it):
lapply(multiExpr, lapply, dim)


# Create an object (list) holding the module labels for each set:
colorList <- list(moduleColors16.custom, moduleColors8.custom);
# Components of the list must be named so that the names can be matched to the names of multiExpr
names(colorList) <- setLabels;

mp_infected <-
  modulePreservation(
    multiExpr,
    colorList,
    networkType = "signed",
    corFnc = "bicor",
    corOptions = "maxPOutliers = 0.1",
    referenceNetworks = c(1:2),
    loadPermutedStatistics = FALSE,
    nPermutations = 200,
    verbose = 3
  )
```

Preservation stats for 16dpp network:

```
presData16dppRef <- tibble(moduleColor = rownames(mp_infected$preservation$Z[[1]][[2]]),
                           Zsummary = mp_infected$preservation$Z[[1]][[2]]$Zsummary.pres,
                           medianRank = mp_infected$preservation$observed[[1]][[2]]$medianRank.pres,
                           moduleSize = mp_infected$preservation$Z[[1]][[2]]$moduleSize) %>%
  filter(!moduleColor == "gold") %>%
  mutate(moduleLabel = match(moduleColor, names(sort(table(moduleColors16.custom), decreasing = TRUE))))

pr1_16 <- presData16dppRef %>%
  ggplot(aes(x = moduleSize, y = Zsummary)) +
  geom_point(aes(color = moduleColor), size = 4) +
  ggrepel::geom_text_repel(aes(label = moduleLabel), force = 5) +
  geom_hline(yintercept = 2, linetype = 2, color = "red") +
  geom_hline(yintercept = 10, linetype = 2, color = "darkgreen") +
```

```
  scale_color_identity()

pr2_16 <- presData16dppRef %>%
  ggplot(aes(x = moduleSize, y = medianRank)) +
  geom_point(aes(color = moduleColor), size = 4) +
  ggrepel::geom_text_repel(aes(label = moduleLabel), force = 5) +
  scale_color_identity()

pr3_16 <- presData16dppRef %>%
  ggplot(aes(x = Zsummary, y = medianRank)) +
  geom_point(aes(color = moduleColor), size = 4) +
  ggrepel::geom_text_repel(aes(label = moduleLabel), force = 5) +
  scale_color_identity()
```

Now presrevation of the 8dpp network:

```
presData8dppRef <- tibble(moduleColor = rownames(mp_infected$preservation$Z[[2]][[1]]),
                          Zsummary = mp_infected$preservation$Z[[2]][[1]]$Zsummary.pres,
                          medianRank = mp_infected$preservation$observed[[2]][[1]]$medianRank.pres,
                          moduleSize = mp_infected$preservation$Z[[2]][[1]]$moduleSize) %>%
  filter(!moduleColor == "gold") %>%
  mutate(moduleLabel = match(moduleColor, names(sort(table(moduleColors8.custom), decreasing = TRUE))))

pr1_8 <- presData8dppRef %>%
  ggplot(aes(x = moduleSize, y = Zsummary)) +
  geom_point(aes(color = moduleColor), size = 4) +
  ggrepel::geom_text_repel(aes(label = moduleLabel), force = 5) +
  geom_hline(yintercept = 2, linetype = 2, color = "red") +
  geom_hline(yintercept = 10, linetype = 2, color = "darkgreen") +
  scale_color_identity()

pr2_8 <- presData8dppRef %>%
  ggplot(aes(x = moduleSize, y = medianRank)) +
  geom_point(aes(color = moduleColor), size = 4) +
  ggrepel::geom_text_repel(aes(label = moduleLabel), force = 5) +
  scale_color_identity()

pr3_8 <- presData8dppRef %>%
  ggplot(aes(x = Zsummary, y = medianRank)) +
  geom_point(aes(color = moduleColor), size = 4) +
  ggrepel::geom_text_repel(aes(label = moduleLabel), force = 5) +
  scale_color_identity()
```

```
cowplot::plot_grid(pr1_8, pr1_16, pr2_8, pr2_16, pr3_8, pr3_16, ncol = 2)
```

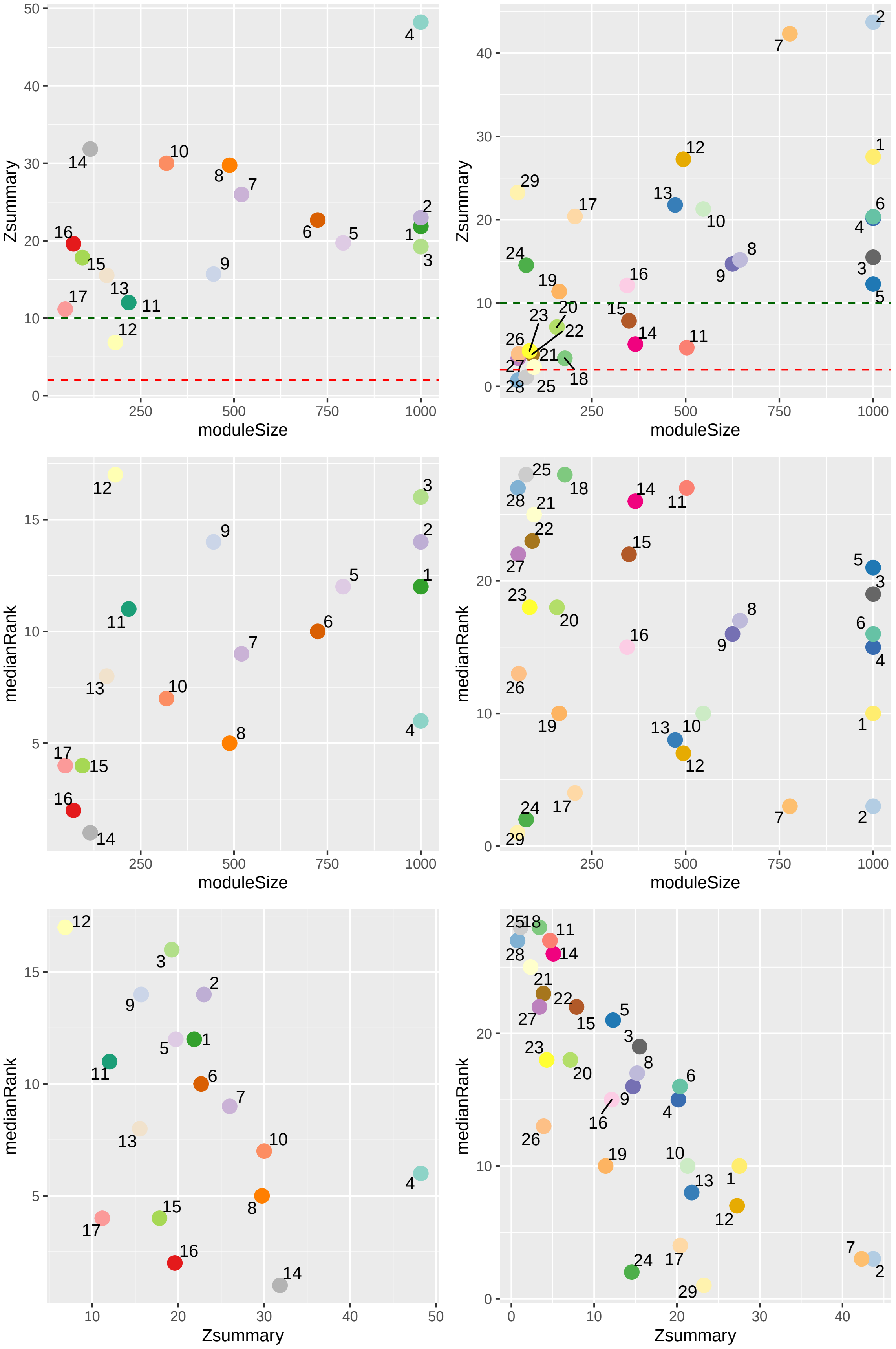
Zsummary
moduleSize
Zsummary
moduleSize
medianRank
moduleSize
medianRank
moduleSize
medianRank
Zsummary
medianRank
Zsummary

Plot alluvial comparison of modules

```
### currently requires ggplot2_3.2.1 and ggalluvial_0.10.0 to work with ggnewscale to have to different

networkLodes <- as.data.frame(overlapTable(moduleLabels16, moduleLabels8)$countTable) %>%
  mutate(module16dpp = rownames(.)) %>%
  gather(key = "module8dpp", value = "overlap", -module16dpp) %>%
  left_join(as.data.frame(overlapTable(moduleLabels16, moduleLabels8)$pTable) %>%
              mutate(module16dpp = rownames(.)) %>%
              gather(key = "module8dpp", value = "pvalue", -module16dpp)
  ) %>%
  mutate(
    # module16dpp = fct_relevel(module16dpp, as.character(c(1:23, 0))),
    # module8dpp = fct_relevel(module8dpp, as.character(1:14)),
    `16dpp` = fct_rev(fct_inorder(rep(customColorOrder16[-1], length(customColorOrder8[-1])))),
    `8dpp` = fct_rev(fct_inorder(rep(customColorOrder8[-1], each = length(customColorOrder16[-1]))))
  ) %>%
  to_lodes_form(key = "Network", value = "module", id = "Cohort", -pvalue, -overlap, -module16dpp, -modu
  mutate(padj = p.adjust(pvalue),
         isSig = ifelse(padj < 0.001, "Yes", "No"),
         moduleSize = c(
           rep(sort(table(moduleColors16.custom), decreasing = TRUE), length(customColorOrder8[-1])),
           rep(sort(table(moduleColors8.custom), decreasing = TRUE), each = length(customColorOrder16[-
         ),
         moduleLabel = ifelse(Network == "16dpp", as.character(module16dpp), as.character(module8dpp))
         )


sixC <- ggplot(data = networkLodes,
         aes(
           x = Network,
           y = overlap,
           stratum = module,
           alluvium = Cohort
         )) +
    geom_stratum(aes(fill = module, color = module)) +
    scale_fill_identity() +
    scale_color_identity() +
    scale_alpha_manual(values=c(0.25, 1)) +
    ggnewscale::new_scale_fill() +
    ggnewscale::new_scale_color() +
    geom_flow(aes(fill = isSig)) +
    scale_fill_viridis_d(direction = 1, begin = 0, end = 0.55,
                         name = "Significant Overlap (Fisher's Exact, FDR < 0.001)"
                        ) +
    geom_text(stat = "stratum", aes(label = ifelse(
    moduleSize > 250, as.character(moduleLabel), NA
    ))) +
    ggrepel::geom_text_repel(data = filter(networkLodes, Network == "8dpp"),
                             stat = "stratum", aes(label = ifelse(moduleSize <= 250,
                                                                  moduleLabel,
                                                                  NA)),
                             nudge_x = .5) +
    ggrepel::geom_text_repel(data = filter(networkLodes, Network == "16dpp"),
```

```
                         stat = "stratum", aes(label = ifelse(moduleSize <= 250,
                                                              moduleLabel,
                                                              NA)),
                         nudge_x = -.5) +
    coord_flip() +
    scale_y_continuous(breaks = seq(0, length(infection16dpp) + 1000, 1000),
                       name = "Number of genes") +
    scale_x_discrete(labels = c("8dpp" = "Susceptible\nmodules\n(8dpp)",
                                "16dpp" = "Resistant\nmodules\n(16dpp)")) +
     cowplot::theme_cowplot(font_size = 14) +
    theme(legend.direction = "horizontal", legend.position = c(0.05, 0.1))

sixC
```

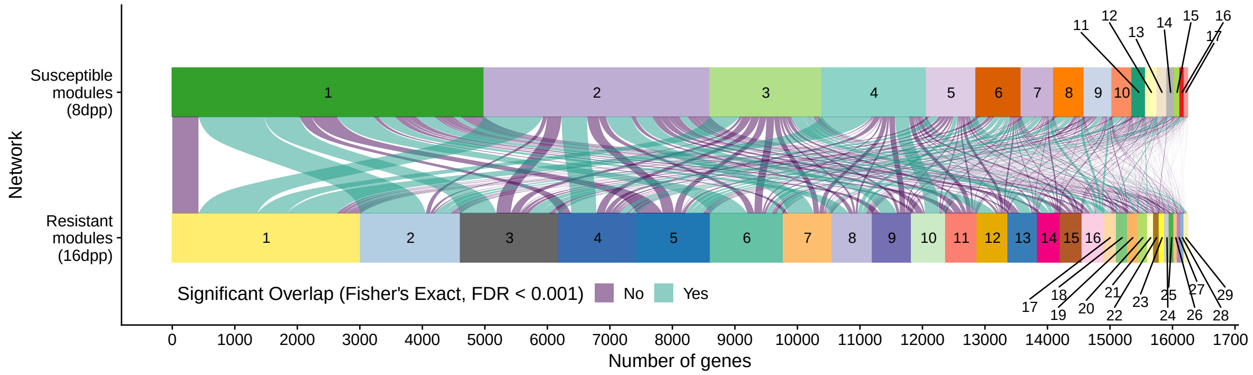

Plot VST expression data for each module

```
moduleExp16 <-
  as.data.frame(assay(vst[colnames(infection16dpp), ])) %>%
  mutate(gene = rownames(.),
         module = moduleColors16.custom,
         moduleLabel = moduleLabels16) %>%
  gather("sample", "counts",-gene, -module, -moduleLabel) %>%
  mutate(age = rep(vst$age, each = ncol(infection16dpp)),
         timepoint = rep(str_remove(vst$timepoint, "T"), each =  ncol(infection16dpp)),
         treatment = rep(vst$treatment, each =  ncol(infection16dpp))
  ) %>%
  left_join(., as_tibble(table(moduleColors16.custom)), by = c("module" = "moduleColors16.custom")) %>%
  mutate(timepoint = as.numeric(timepoint),
         module = fct_infreq(module),
         facetLabel = fct_infreq(paste0("R", moduleLabel, " (n=", n,")"))
         )

modSubset <- unique(moduleExp16$moduleLabel)
modSubset <- modSubset[!modSubset %in% 0]
```

Plot variance stabilized transformed read counts for resistant modules and overlay corrisponding values for other treament conditions. Figure 7.

```
p1 <- moduleExp16 %>%
  filter(moduleLabel %in% modSubset) %>%
  ggplot() +
  stat_summary(
    data = filter(moduleExp16, treatment == "Inoc" &
```

```r
                    moduleLabel %in% modSubset),
    fun.data = mean_se,
    geom = "ribbon",
    aes(x = timepoint, y = counts, fill = age),
    alpha = 0.2
  ) +
  stat_summary(
    data = filter(moduleExp16, treatment == "Cont" &
                    moduleLabel %in% modSubset),
    fun.data = mean_se,
    geom = "ribbon",
    aes(x = timepoint, y = counts, fill = age),
    alpha = 0.2
  ) +
  stat_summary(
    fun.y = mean,
    geom = "line",
    aes(
      x = timepoint,
      y = counts,
      color = age,
      linetype = treatment
    )
  ) +
  stat_summary(
    fun.y = mean,
    geom = "point",
    aes(
      x = timepoint,
      y = counts,
      group = paste0(age, treatment)
    ), shape = 16,
    color = rep(rep(c("#00BFC4", "#00BFC4", "#F8766D", "#F8766D"), each = 7), length(modSubset))
  ) +
  facet_wrap(~ facetLabel, scales = "free", ncol = 5) +
  scale_x_continuous(breaks = c(0, 2, 4, 8, 12, 18, 24)) +
  labs(x = "Hours post inoculation", y = "Variance stabilizing transformed read count") +
  guides(linetype = guide_legend(title="Treatment"),
         color = guide_legend(title = "Age"),
         fill = guide_legend(title = "Age")) +
  scale_linetype_discrete(labels = c("Control", "Inoculated")) +
  scale_color_discrete(labels = c("8 dpp", "16 dpp")) +
  scale_fill_discrete(labels = c("8 dpp", "16 dpp")) +
  cowplot::theme_cowplot(font_size = 11) +
    theme(strip.text = element_text(
        colour = "grey10",
        size = rel(0.8),
        margin = margin(0.5 * 7, 0.5 * 7, 0.5 * 7, 0.5 * 7)
        )
    ) +
    theme(legend.position=c(0.85, 0.05),
        legend.box = 'vertical',
        legend.margin = margin(t = -5)) +
```

```
    cowplot::panel_border()


g1 <- ggplot_gtable(ggplot_build(p1))
stripr <- which(grepl('strip-t', g1$layout$name) & grepl("gTree", g1$grobs))

fills <- names(sort(table(moduleColors16.custom), decreasing = T))
fills <- names(sort(table(moduleColors16.custom), decreasing = T))[unlist(rev(split(1:length(fills), ce[illegible]


k <- 1

for (i in stripr) {
    j <- which(grepl('rect', g1$grobs[[i]]$grobs[[1]]$childrenOrder))
    g1$grobs[[i]]$grobs[[1]]$children[[j]]$gp$fill <- fills[k]
    k <- k+1
}
grid::grid.draw(g1)
```

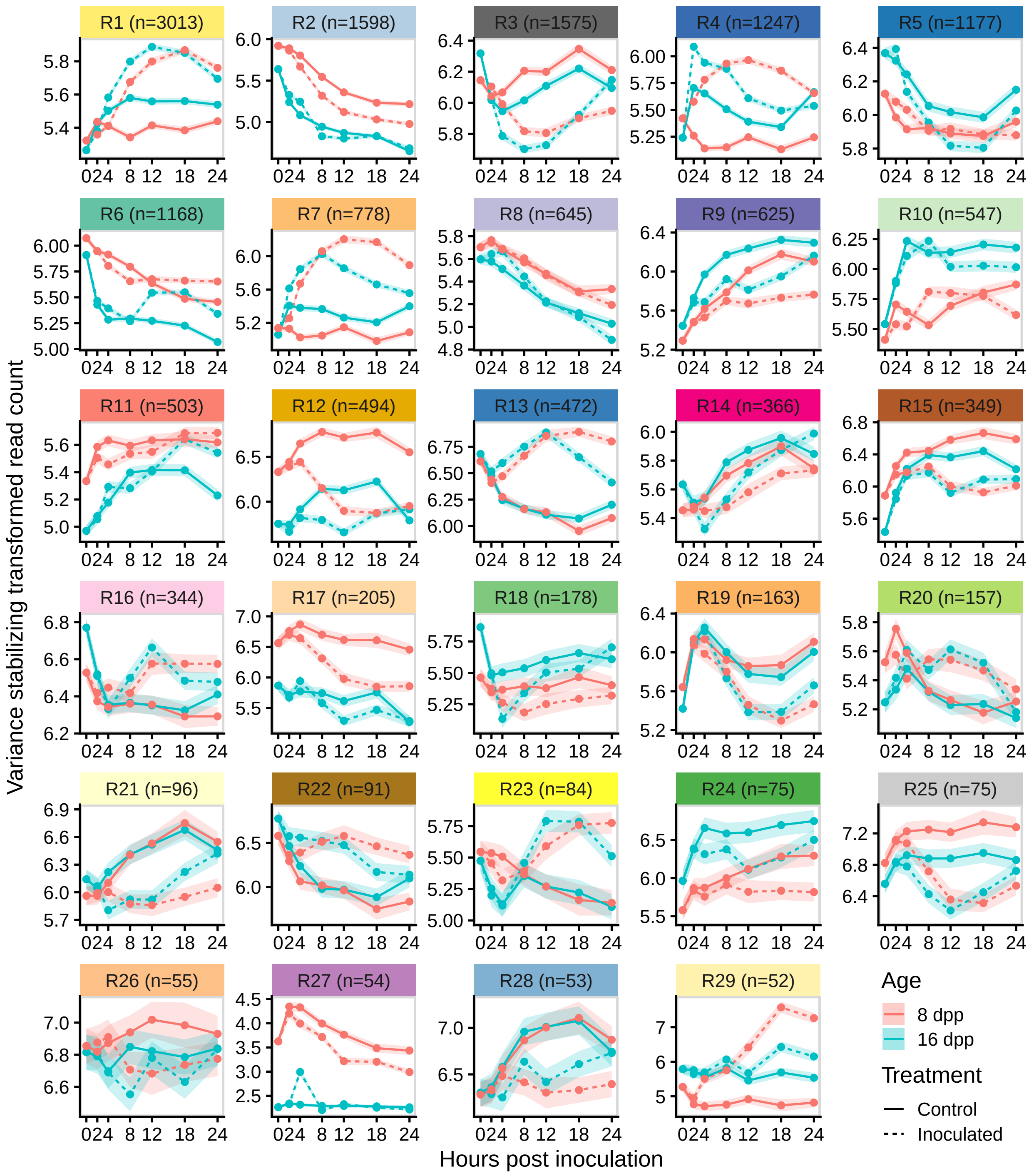

Plot VST expression data for each module in susceptible network:

```
moduleExp8 <-
    as.data.frame(assay(vst[colnames(infection8dpp), ])) %>%
    mutate(gene = rownames(.),
           module = moduleColors8.custom,
           moduleLabel = moduleLabels8) %>%
    gather("sample", "counts",-gene, -module, -moduleLabel) %>%
    mutate(age = rep(vst$age, each = ncol(infection8dpp)),
```

```
           timepoint = rep(str_remove(vst$timepoint, "T"), each =  ncol(infection8dpp)),
           treatment = rep(vst$treatment, each =  ncol(infection8dpp))
    ) %>%
    left_join(., as_tibble(table(moduleColors8.custom)), by = c("module" = "moduleColors8.custom")) %>%
    mutate(timepoint = as.numeric(timepoint),
           module = fct_infreq(module),
           facetLabel = fct_infreq(paste0("S", moduleLabel, " (n=", n,")"))
    )

modSubset <- unique(moduleExp8$moduleLabel)
modSubset <- modSubset[!modSubset %in% 0]
```

Plot variance stabilized transformed read counts for susceptible modules and overlay corrisponding values for other treament conditions.

```
moduleExp8  %>%
  filter(moduleLabel %in% modSubset) %>%
  ggplot() +
  stat_summary(
    data = filter(moduleExp8, treatment == "Inoc" &
                    moduleLabel %in% modSubset),
    fun.data = mean_se,
    geom = "ribbon",
    aes(x = timepoint, y = counts, fill = age),
    alpha = 0.2
  ) +
  stat_summary(
    data = filter(moduleExp8, treatment == "Cont" &
                    moduleLabel %in% modSubset),
    fun.data = mean_se,
    geom = "ribbon",
    aes(x = timepoint, y = counts, fill = age),
    alpha = 0.2
  ) +
  stat_summary(
    fun.y = mean,
    geom = "line",
    aes(
      x = timepoint,
      y = counts,
      color = age,
      linetype = treatment
    )
  ) +
  stat_summary(
    fun.y = mean,
    geom = "point",
    aes(
      x = timepoint,
      y = counts,
      group = paste0(age, treatment)
    ), shape = 16,
    color = rep(rep(c("#00BFC4", "#00BFC4", "#F8766D", "#F8766D"), each = 7), length(modSubset))
  ) +
```

```
  facet_wrap(~ facetLabel, scales = "free", ncol = 5) +
  scale_x_continuous(breaks = c(0, 2, 4, 8, 12, 18, 24)) +
  labs(x = "Hours post inoculation", y = "Variance stabilizing transformed read count") +
  guides(linetype = guide_legend(title="Treatment"),
         color = guide_legend(title = "Age"),
         fill = guide_legend(title = "Age")) +
  scale_linetype_discrete(labels = c("Control", "Inoculated")) +
  scale_color_discrete(labels = c("8 dpp", "16 dpp")) +
  scale_fill_discrete(labels = c("8 dpp", "16 dpp")) +
  cowplot::theme_cowplot(font_size = 11) +
    theme(strip.text = element_text(
        colour = "grey10",
        size = rel(0.8),
        margin = margin(0.5 * 7, 0.5 * 7, 0.5 * 7, 0.5 * 7)
        )
    ) +
    theme(legend.position=c(0.85, 0.05),
        legend.box = 'vertical',
        legend.margin = margin(t = -5)) +
    cowplot::panel_border()
```

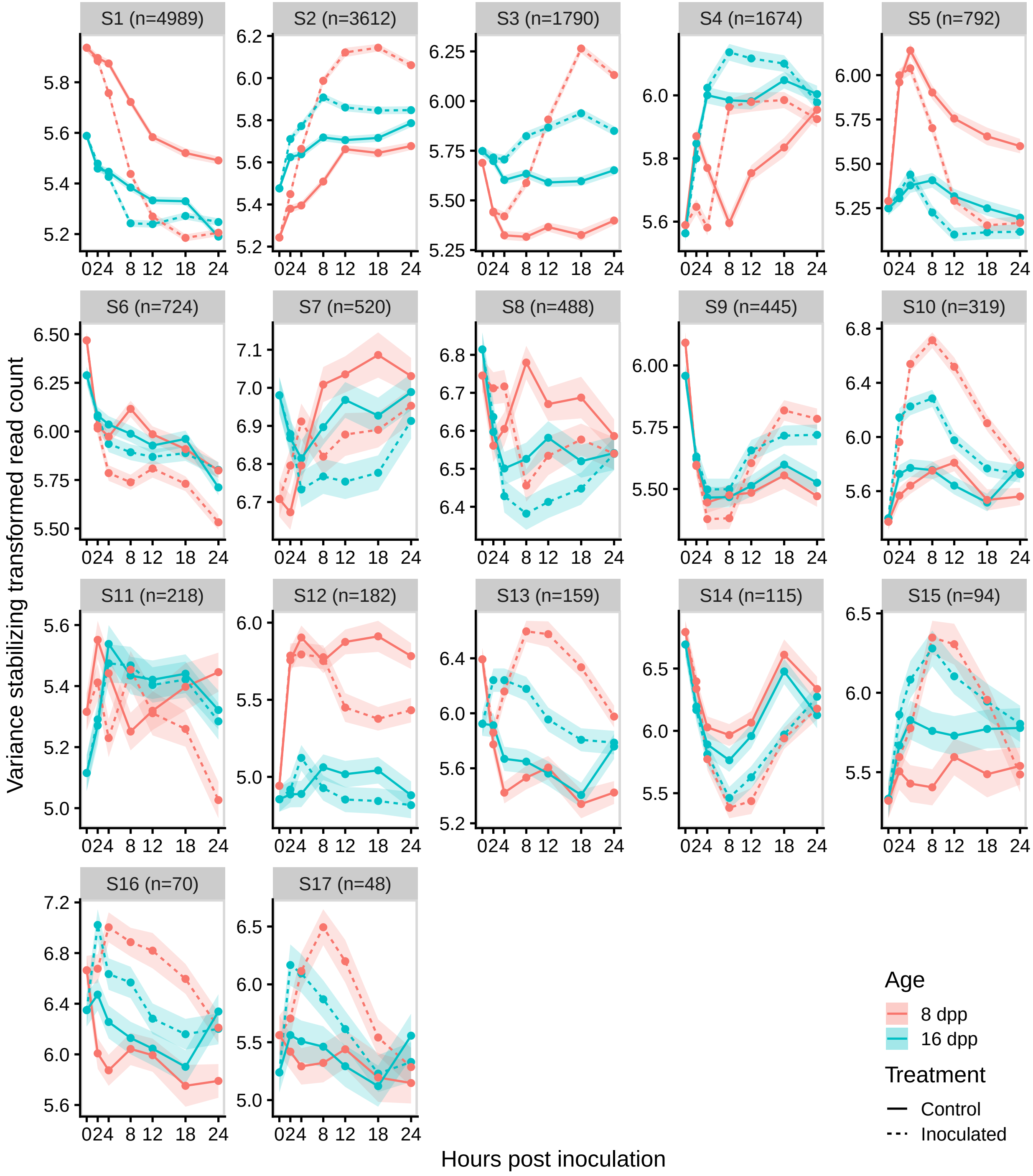


```r
# g1 <- ggplot_gtable(ggplot_build(p1))
# stripr <- which(grepl('strip-t', g1$layout$name) & grepl("gTree", g1$grobs))
#
# fills <- names(sort(table(moduleColors8.custom), decreasing = T))
# fills <- names(sort(table(moduleColors8.custom), decreasing = T))[unlist(rev(split(1:length(fills), c
#
#
# k <- 1
```

```
#
# for (i in stripr) {
#     j <- which(grepl('rect', g1$grobs[[i]]$grobs[[1]]$childrenOrder))
#     g1$grobs[[i]]$grobs[[1]]$children[[j]]$gp$fill <- fills[k]
#     k <- k+1
# }
# grid::grid.draw(g1)
```

Arrange the read count data in tidy format and add module numbers and log counts

```
modulecounts16 <-
  as.data.frame(counts(dds[colnames(infection16dpp), ], normalized = T)) %>%
  mutate(gene = rownames(.),
         module = moduleColors16.custom,
         moduleLabel = moduleLabels16) %>%
  gather("sample", "counts",-gene, -module, -moduleLabel) %>%
  mutate(age = rep(vst$age, each = ncol(infection16dpp)),
         timepoint = rep(str_remove(vst$timepoint, "T"), each =  ncol(infection16dpp)),
         treatment = rep(vst$treatment, each =  ncol(infection16dpp))
  ) %>%
  left_join(., as_tibble(table(moduleColors16.custom)), by = c("module" = "moduleColors16.custom")) %>%
  mutate(timepoint = as.numeric(timepoint),
         module = fct_infreq(module),
         facetLabel = fct_infreq(paste0("R", moduleLabel, " (n=", n,")")),
         logCounts = log2(counts + 0.5)
  )

modulecounts8 <-
  as.data.frame(counts(dds[colnames(infection8dpp), ], normalized = T)) %>%
  mutate(gene = rownames(.),
         module = moduleColors8.custom,
         moduleLabel = moduleLabels8) %>%
  gather("sample", "counts",-gene, -module, -moduleLabel) %>%
  mutate(age = rep(vst$age, each = ncol(infection8dpp)),
         timepoint = rep(str_remove(vst$timepoint, "T"), each =  ncol(infection8dpp)),
         treatment = rep(vst$treatment, each =  ncol(infection8dpp))
  ) %>%
  left_join(., as_tibble(table(moduleColors8.custom)), by = c("module" = "moduleColors8.custom")) %>%
  mutate(timepoint = as.numeric(timepoint),
         module = fct_infreq(module),
         facetLabel = fct_infreq(paste0("S", moduleLabel, " (n=", n,")")),
         logCounts = log2(counts + 0.5)
  )
```

Genes in ModS1 and their expression patterns in other modules. Figure 6D

```
networkLodes %>%
    filter(module8dpp == 1, isSig == "Yes")
```

```
##    module16dpp module8dpp overlap        pvalue Cohort Network  module
## 1            2          1    1042 3.194379e-198      2   16dpp #B3CDE3
## 2            3          1     572  3.320226e-07      3   16dpp #666666
## 3            6          1     589  2.472749e-48      6   16dpp #66C2A5
## 4            8          1     266  5.160602e-09      8   16dpp #BEBADA
## 5           12          1     296  3.509969e-42     12   16dpp #E6AB02
```

```
## 6           17          1     115  3.280177e-14     17   16dpp #FED9A6
## 7            2          1    1042 3.194379e-198      2    8dpp #33A02C
## 8            3          1     572  3.320226e-07      3    8dpp #33A02C
## 9            6          1     589  2.472749e-48      6    8dpp #33A02C
## 10           8          1     266  5.160602e-09      8    8dpp #33A02C
## 11          12          1     296  3.509969e-42     12    8dpp #33A02C
## 12          17          1     115  3.280177e-14     17    8dpp #33A02C
##              padj isSig moduleSize moduleLabel
## 1  3.149658e-195   Yes       1598           2
## 2   2.994843e-04   Yes       1575           3
## 3   2.403512e-45   Yes       1168           6
## 4   4.737433e-06   Yes        645           8
## 5   3.404670e-39   Yes        494          12
## 6   3.089926e-11   Yes        205          17
## 7  3.149658e-195   Yes       4989           1
## 8   2.994843e-04   Yes       4989           1
## 9   2.403512e-45   Yes       4989           1
## 10  4.737433e-06   Yes       4989           1
## 11  3.404670e-39   Yes       4989           1
## 12  3.089926e-11   Yes       4989           1
```

```
S1_R2_genes <- moduleExp8 %>%
    dplyr::select(gene, moduleLabel) %>%
    left_join(dplyr::select(moduleExp16, gene, moduleLabel), by = "gene", suffix = c(".8dpp", ".16dpp")
    filter(moduleLabel.8dpp == 1) %>%
    filter(moduleLabel.16dpp == 2) %>%
    pull(gene) %>%
    unique()

S1_R3_genes <- moduleExp8 %>%
    dplyr::select(gene, moduleLabel) %>%
    left_join(dplyr::select(moduleExp16, gene, moduleLabel), by = "gene", suffix = c(".8dpp", ".16dpp")
    filter(moduleLabel.8dpp == 1) %>%
    filter(moduleLabel.16dpp == 3) %>%
    pull(gene) %>%
    unique()

S1_R6_genes <- moduleExp8 %>%
    dplyr::select(gene, moduleLabel) %>%
    left_join(dplyr::select(moduleExp16, gene, moduleLabel), by = "gene", suffix = c(".8dpp", ".16dpp")
    filter(moduleLabel.8dpp == 1) %>%
    filter(moduleLabel.16dpp == 6) %>%
    pull(gene) %>%
    unique()

S1_R8_genes <- moduleExp8 %>%
    dplyr::select(gene, moduleLabel) %>%
    left_join(dplyr::select(moduleExp16, gene, moduleLabel), by = "gene", suffix = c(".8dpp", ".16dpp")
    filter(moduleLabel.8dpp == 1) %>%
    filter(moduleLabel.16dpp == 8) %>%
    pull(gene) %>%
    unique()

S1_R12_genes <- moduleExp8 %>%
```

```
    dplyr::select(gene, moduleLabel) %>%
    left_join(dplyr::select(moduleExp16, gene, moduleLabel), by = "gene", suffix = c(".8dpp", ".16dpp"))
    filter(moduleLabel.8dpp == 1) %>%
    filter(moduleLabel.16dpp == 12) %>%
    pull(gene) %>%
    unique()

S1_R17_genes <- moduleExp8 %>%
    dplyr::select(gene, moduleLabel) %>%
    left_join(dplyr::select(moduleExp16, gene, moduleLabel), by = "gene", suffix = c(".8dpp", ".16dpp"))
    filter(moduleLabel.8dpp == 1) %>%
    filter(moduleLabel.16dpp == 17) %>%
    pull(gene) %>%
    unique()

consModExp <- bind_rows("S1 vs R2\nGene overlap: 1042" = filter(moduleExp8, gene %in% S1_R2_genes),
                        "S1 vs R3\nGene overlap: 572" = filter(moduleExp8, gene %in% S1_R3_genes),
                        "S1 vs R6\nGene overlap: 589" = filter(moduleExp8, gene %in% S1_R6_genes),
                        "S1 vs R8\nGene overlap: 266" = filter(moduleExp8, gene %in% S1_R8_genes),
                        "S1 vs R12\nGene overlap: 296" = filter(moduleExp8, gene %in% S1_R12_genes),
                        "S1 vs R17\nGene overlap: 115" = filter(moduleExp8, gene %in% S1_R17_genes),
                        .id = "modComp") %>%
    mutate(modComp = fct_inorder(modComp))

sixD <- filter(consModExp, treatment == "Inoc")  %>%
  ggplot() +
  stat_summary(
    data = filter(consModExp, treatment == "Inoc"),
    fun.data = mean_se,
    geom = "ribbon",
    aes(x = timepoint, y = counts, fill = age),
    alpha = 0.2
  ) +
  # stat_summary(
  #   data = filter(consModExp, treatment == "Cont"),
  #   fun.data = mean_se,
  #   geom = "ribbon",
  #   aes(x = timepoint, y = counts, fill = age),
  #   alpha = 0.2
  # ) +
  stat_summary(
    fun.y = mean,
    geom = "line",
    aes(
      x = timepoint,
      y = counts,
      color = age,
      # linetype = treatment
    )
  ) +
  stat_summary(
    fun.y = mean,
    geom = "point",
```

```
    aes(
      x = timepoint,
      y = counts,
      group = paste0(age, treatment)
    ), shape = 16,
    # color = rep(rep(c("#00BFC4", "#00BFC4", "#F8766D", "#F8766D"), each = 7), 6)
  ) +
  facet_wrap(~ modComp, scales = "free", ncol = 6) +
  scale_x_continuous(breaks = c(0, 2, 4, 8, 12, 18, 24)) +
  labs(x = "Hours post inoculation", y = "Variance stabilizing transformed read count") +
  guides(#linetype = guide_legend(title="Treatment"),
         color = guide_legend(title = "Age"),
         fill = guide_legend(title = "Age")) +
  scale_color_discrete(labels = c("8 dpp", "16 dpp")) +
  scale_fill_discrete(labels = c("8 dpp", "16 dpp")) +
  cowplot::theme_cowplot(font_size = 11) +
    theme(strip.text = element_text(
        colour = "grey10",
        size = rel(0.8),
        margin = margin(0.5 * 7, 0.5 * 7, 0.5 * 7, 0.5 * 7)
        )
    ) +
    theme(legend.position = c(0.925, 0.875),
        # legend.position="bottom",
        # legend.box = 'vertical',
        legend.margin = margin(t = -5)) +
    cowplot::panel_border()

sixD
```

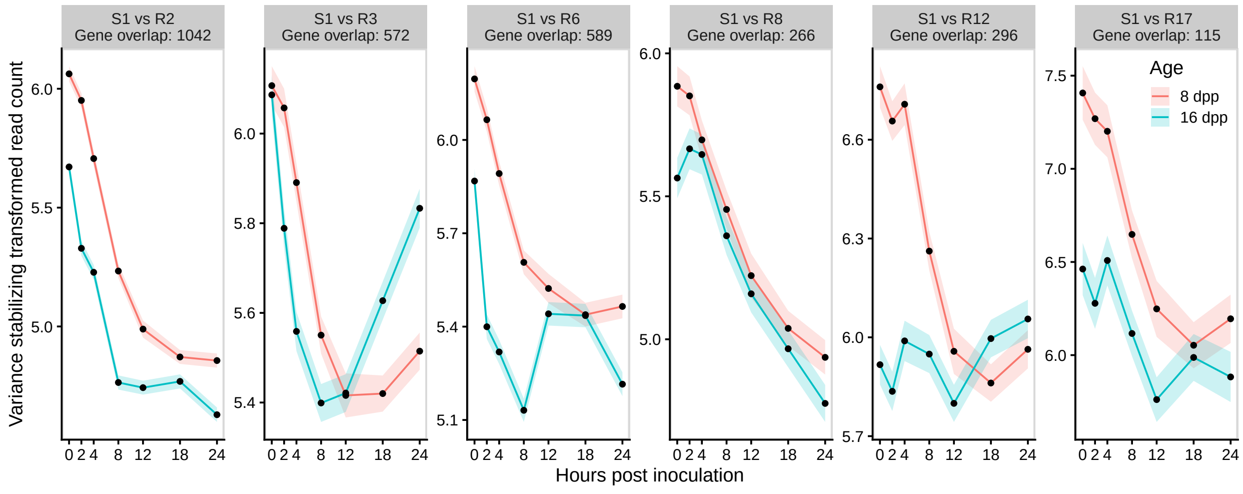

Figure 6:

```
sixA <- cowplot::ggdraw() + cowplot::draw_image(image = "fig6A.png")
sixB <- cowplot::ggdraw() + cowplot::draw_image(image = "fig6B.png")

sixAB <- cowplot::plot_grid(sixA, sixB,
                   ncol = 2,
```

```
                    labels = "AUTO")

cowplot::plot_grid(sixAB,
                   sixC,
                   sixD,
                   ncol = 1,
                   labels = c("", "C", "D"),
                   rel_heights = c(3, 2, 2.1))
```

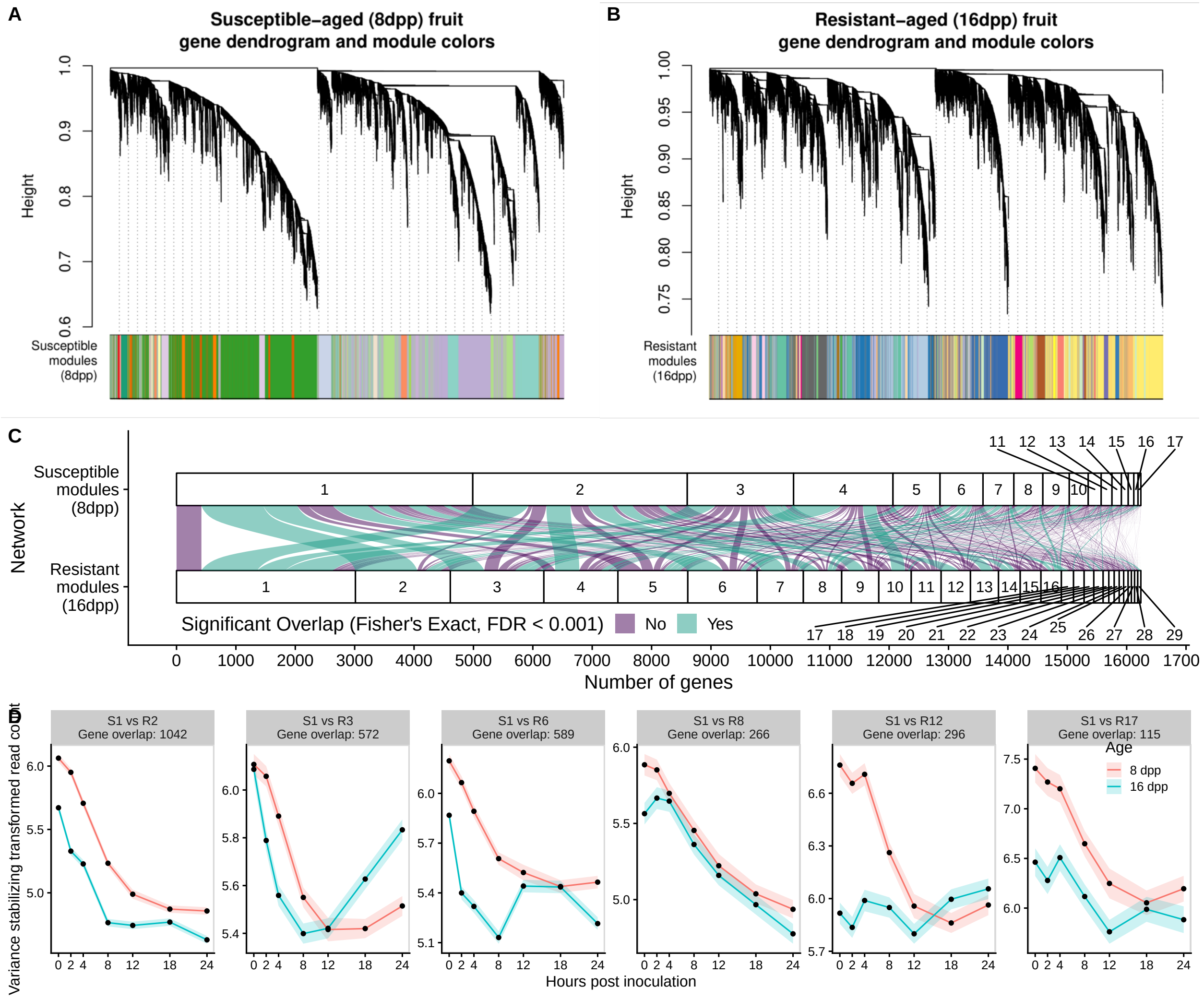


```
# pdf(file = "fig6.pdf", width = 7, 9)
# cowplot::plot_grid(drPlot, poin, mic,
#                    ncol = 1,
#                    labels = "AUTO",
#                    rel_heights = c(3, 1.5, 1.8))
# dev.off()
```

Export all genes and GO term enrichment results for Resistant network modules

```
modSubset <- unique(moduleExp16$moduleLabel)
modSubset <- modSubset[!modSubset %in% 0]
```

```
outGenes <- list()
k <- 1
for (i in 1:max(modSubset)) {
    outGenes[[k]] <- modulecounts16 %>%
        filter(moduleLabel %in% i) %>%
        dplyr::select(-module,  -moduleLabel, -age, -timepoint, -treatment ,-n, -facetLabel, -logCounts
        spread(sample, counts)
    k <- k + 1
}

names(outGenes) <- paste0("Module R", 1:max(modSubset))

library(writexl)

write_xlsx(outGenes, "Supplementary File 2_Resistant_Modules_Genes.xlsx", col_names = T)
```

```
modSubset <- unique(moduleExp16$moduleLabel)
modSubset <- modSubset[!modSubset %in% 0]

GOres <- list()
k <- 1
for (i in 1:max(modSubset)) {
GOres[[k]] <- moduleGOterms(
  network = "16dpp",
  mod = i,
  nodeSize = 100,
  n = 500
  ) %>% filter(Fisher.weight01 < 0.05)
k <- k + 1
}

names(GOres) <- paste0("Module R", 1:max(modSubset))
```

```
write_xlsx(GOres, "Supplementary File 4_Resistant_Modules_GO_Enrichment.xlsx", col_names = T)
```

Export gene module assignments for susceptible network:

```
modSubset <- unique(moduleExp8$moduleLabel)
modSubset <- modSubset[!modSubset %in% 0]

outGenes <- list()
k <- 1
for (i in 1:max(modSubset)) {
    outGenes[[k]] <- modulecounts8 %>%
        filter(moduleLabel %in% i) %>%
        dplyr::select(-module,  -moduleLabel, -age, -timepoint, -treatment ,-n, -facetLabel, -logCounts
        spread(sample, counts)
    k <- k + 1
}

names(outGenes) <- paste0("Module S", 1:max(modSubset))

library(writexl)
```

```r
write_xlsx(outGenes, "Supplementary File 3_Susceptible_Modules_Genes.xlsx", col_names = T)
```

Which have significant interaction effects and then in which spline portion do they have an interaction

```r
library(splines)

modulecounts16 %>%
  filter(treatment == "Inoc") %>%
  nest_by(moduleLabel) %>%
  mutate(anova = list(anova(lm(logCounts ~ age*ns(timepoint, df = 4), data = data)))) %>%
  summarise(broom::tidy(anova)) %>%
  filter(str_detect(term, "age:ns"), p.value < 0.05) %>% kable()
```

moduleLabel	term	df	sumsq	meansq	statistic	p.value
1	age:ns(timepoint, df = 4)	4	329.99965	82.49991	15.934368	0.0000000
2	age:ns(timepoint, df = 4)	4	314.39321	78.59830	14.955229	0.0000000
3	age:ns(timepoint, df = 4)	4	556.14154	139.03539	27.257186	0.0000000
4	age:ns(timepoint, df = 4)	4	1372.47125	343.11781	64.269490	0.0000000
5	age:ns(timepoint, df = 4)	4	330.53709	82.63427	14.554743	0.0000000
6	age:ns(timepoint, df = 4)	4	405.35071	101.33768	23.749391	0.0000000
7	age:ns(timepoint, df = 4)	4	940.10468	235.02617	42.185627	0.0000000
8	age:ns(timepoint, df = 4)	4	126.22441	31.55610	5.047284	0.0004597
9	age:ns(timepoint, df = 4)	4	73.84148	18.46037	3.210159	0.0120955
10	age:ns(timepoint, df = 4)	4	172.79071	43.19768	8.528060	0.0000007
11	age:ns(timepoint, df = 4)	4	135.22117	33.80529	7.716836	0.0000033
12	age:ns(timepoint, df = 4)	4	503.94481	125.98620	25.395775	0.0000000
13	age:ns(timepoint, df = 4)	4	223.30571	55.82643	13.362317	0.0000000
14	age:ns(timepoint, df = 4)	4	106.54735	26.63684	6.508226	0.0000315
15	age:ns(timepoint, df = 4)	4	207.59172	51.89793	10.683718	0.0000000
16	age:ns(timepoint, df = 4)	4	72.22910	18.05727	4.950540	0.0005495
18	age:ns(timepoint, df = 4)	4	98.17228	24.54307	5.643348	0.0001563
19	age:ns(timepoint, df = 4)	4	68.86208	17.21552	3.174732	0.0129011
20	age:ns(timepoint, df = 4)	4	55.10566	13.77642	3.567641	0.0065185
21	age:ns(timepoint, df = 4)	4	41.93763	10.48441	2.463965	0.0431030
22	age:ns(timepoint, df = 4)	4	52.10105	13.02526	3.387247	0.0089643
23	age:ns(timepoint, df = 4)	4	41.79785	10.44946	3.288753	0.0106407
27	age:ns(timepoint, df = 4)	4	104.56769	26.14192	11.520982	0.0000000
29	age:ns(timepoint, df = 4)	4	410.30424	102.57606	19.212257	0.0000000

```r
modulecounts16 %>%
  filter(treatment == "Inoc") %>%
  nest_by(moduleLabel) %>%
  mutate(fit = list(lm(counts ~ age*ns(timepoint, df = 4), data = data))) %>%
  summarise(broom::tidy(fit)) %>%
  filter(str_detect(term, "16dpp:ns"), p.value < 0.05) %>%
  kable()
```

moduleLabel	term	estimate	std.error	statistic	p.value
1	age16dpp:ns(timepoint, df = 4)1	33.716112	12.587751	2.678486	0.0073966
1	age16dpp:ns(timepoint, df = 4)4	-17.708278	8.045915	-2.200903	0.0277447
2	age16dpp:ns(timepoint, df = 4)2	34.164062	12.604516	2.710462	0.0067207
2	age16dpp:ns(timepoint, df = 4)4	27.622086	8.593784	3.214194	0.0013087
3	age16dpp:ns(timepoint, df = 4)3	-54.547120	24.827976	-2.197002	0.0280238
3	age16dpp:ns(timepoint, df = 4)4	42.540349	12.464383	3.412952	0.0006430
4	age16dpp:ns(timepoint, df = 4)1	-50.846834	25.008103	-2.033214	0.0420360
4	age16dpp:ns(timepoint, df = 4)2	-47.739781	23.444978	-2.036248	0.0417306
4	age16dpp:ns(timepoint, df = 4)4	-52.898562	15.984832	-3.309297	0.0009360
6	age16dpp:ns(timepoint, df = 4)2	25.219679	8.301942	3.037805	0.0023844
7	age16dpp:ns(timepoint, df = 4)2	-89.246529	39.228200	-2.275060	0.0229089
7	age16dpp:ns(timepoint, df = 4)4	-66.615752	26.745863	-2.490694	0.0127544
12	age16dpp:ns(timepoint, df = 4)1	84.857949	42.235479	2.009163	0.0445331
12	age16dpp:ns(timepoint, df = 4)2	79.431336	39.595562	2.006067	0.0448624
12	age16dpp:ns(timepoint, df = 4)4	84.269099	26.996331	3.121502	0.0018018
13	age16dpp:ns(timepoint, df = 4)2	-72.656693	24.064806	-3.019210	0.0025377
13	age16dpp:ns(timepoint, df = 4)3	-81.096923	32.682187	-2.481380	0.0130959
13	age16dpp:ns(timepoint, df = 4)4	-85.829751	16.407431	-5.231151	0.0000002
15	age16dpp:ns(timepoint, df = 4)3	139.282482	68.398006	2.036353	0.0417333
16	age16dpp:ns(timepoint, df = 4)3	-93.437517	30.767404	-3.036900	0.0023946
17	age16dpp:ns(timepoint, df = 4)4	237.608300	115.102668	2.064316	0.0390184
18	age16dpp:ns(timepoint, df = 4)3	-46.351422	19.764345	-2.345204	0.0190433
22	age16dpp:ns(timepoint, df = 4)4	-55.705134	28.027657	-1.987506	0.0469395
27	age16dpp:ns(timepoint, df = 4)1	13.974305	3.277604	4.263573	0.0000210
27	age16dpp:ns(timepoint, df = 4)2	8.463938	3.072738	2.754526	0.0059260
27	age16dpp:ns(timepoint, df = 4)4	14.381642	2.094999	6.864749	0.0000000
29	age16dpp:ns(timepoint, df = 4)2	-496.697392	99.904288	-4.971732	0.0000007
29	age16dpp:ns(timepoint, df = 4)3	-413.950980	135.679075	-3.050957	0.0023094
29	age16dpp:ns(timepoint, df = 4)4	-275.218091	68.114936	-4.040495	0.0000552

normalized gene expression and fitted curves with splined time

```
pME <- modulecounts16 %>%
  filter(treatment == "Inoc") %>%
  group_by(moduleLabel) %>%
  ggplot(aes(x = timepoint, y = logCounts, color = age)) +
  stat_summary(fun.data = "mean_se", size = 0.25) +
  geom_smooth(method = lm,
              formula = y ~ splines::ns(x, df = 4), linetype = 2) +
  facet_wrap(~ moduleLabel, scales = "free", ncol = 5) +
  labs(x = "Hours post inoculation", y = "Log(Normalized counts)") +
  theme_gray() +
  theme(legend.position="bottom")

gME <- ggplot_gtable(ggplot_build(pME))
stripr <- which(grepl('strip-t', gME$layout$name) & grepl("gTree", gME$grobs))

fills <- names(sort(table(moduleColors16.custom), decreasing = T))
fills <- names(
  sort(
    table(
      moduleColors16.custom), decreasing = T)
  )[unlist(rev(split(1:length(fills), ceiling(seq_along(1:length(fills))/5)))),
```

```
            use.names = F)]


k <- 1

for (i in stripr) {
    j <- which(grepl('rect', gME$grobs[[i]]$grobs[[1]]$childrenOrder))
    gME$grobs[[i]]$grobs[[1]]$children[[j]]$gp$fill <- fills[k]
    k <- k+1
}
grid::grid.draw(gME)
```

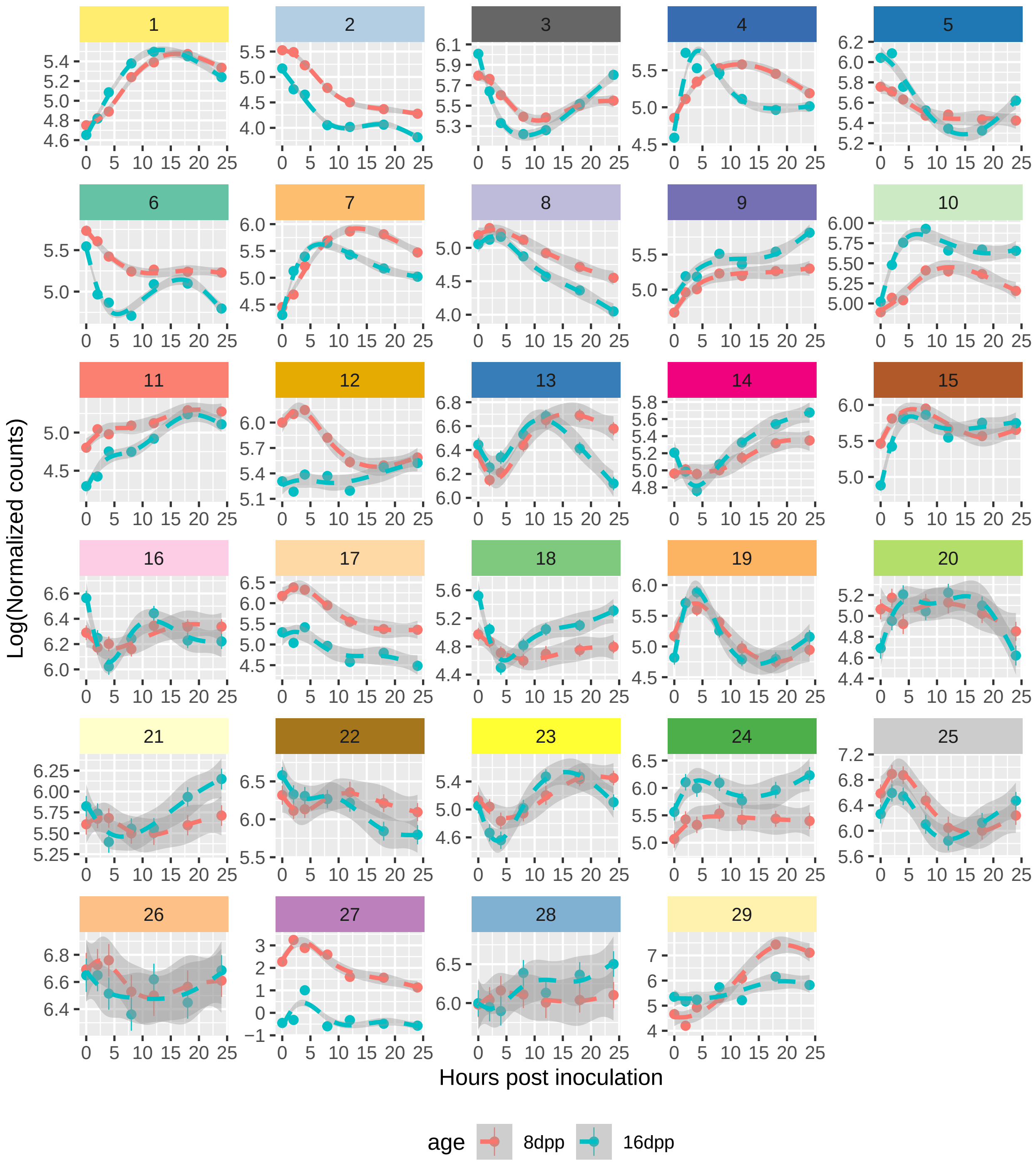

Which modules have an interaction in the first or second spline portions

```
library(splines)

anovaModExp <- modulecounts16 %>%
  filter(treatment == "Inoc") %>%
  nest_by(moduleLabel) %>%
  mutate(fit = list(lm(counts ~ age*ns(timepoint, df = 4), data = data))) %>%
  summarise(broom::tidy(fit)) %>%
```

```
  filter(str_detect(term, "age16dpp\\:ns.*[1-2]"), p.value < 0.05)

anovaModExp
```

```
## # A tibble: 12 x 6
## # Groups:   moduleLabel [9]
##    moduleLabel term                         estimate std.error statistic    p.value
##          <dbl> <chr>                           <dbl>     <dbl>     <dbl>      <dbl>
##  1           1 age16dpp:ns(timepoint, d~    33.7      12.6       2.68    7.40e-3
##  2           2 age16dpp:ns(timepoint, d~    34.2      12.6       2.71    6.72e-3
##  3           4 age16dpp:ns(timepoint, d~   -50.8      25.0      -2.03    4.20e-2
##  4           4 age16dpp:ns(timepoint, d~   -47.7      23.4      -2.04    4.17e-2
##  5           6 age16dpp:ns(timepoint, d~    25.2       8.30      3.04    2.38e-3
##  6           7 age16dpp:ns(timepoint, d~   -89.2      39.2      -2.28    2.29e-2
##  7          12 age16dpp:ns(timepoint, d~    84.9      42.2       2.01    4.45e-2
##  8          12 age16dpp:ns(timepoint, d~    79.4      39.6       2.01    4.49e-2
##  9          13 age16dpp:ns(timepoint, d~   -72.7      24.1      -3.02    2.54e-3
## 10          27 age16dpp:ns(timepoint, d~    14.0       3.28      4.26    2.10e-5
## 11          27 age16dpp:ns(timepoint, d~     8.46      3.07      2.75    5.93e-3
## 12          29 age16dpp:ns(timepoint, d~  -497.       99.9      -4.97    7.17e-7
```

```
GOres <- list()

k <- 1
for (i in unique(anovaModExp$moduleLabel)) {
GOres[[k]] <- moduleGOterms(
  network = "16dpp",
  mod = i,
  nodeSize = 100,
  n = 5
  )
k <- k + 1
}

names(GOres) <- unique(anovaModExp$moduleLabel)
```

Plot log2Normalized read counts with a spline of time. Plot top 5 goterms for each module on the side. Figure 8

```
fills <- rev(names(sort(table(moduleColors16.custom), decreasing = T))[unique(anovaModExp$moduleLabel)]

tidyGOres <- bind_rows(GOres, .id = "moduleLabel") %>%
  mutate(moduleLabel = fct_relevel(moduleLabel, as.character(sort(unique(anovaModExp$moduleLabel))))) %
  mutate(color = rep(rev(fills), each = 5))


pME <- modulecounts16 %>%
  filter(moduleLabel %in% anovaModExp$moduleLabel) %>%
  ggplot(aes(x = timepoint, y = logCounts, color = age)) +
  stat_summary(fun.data = "mean_se", size = 0.25) +
  geom_smooth(method = lm,
              formula = y ~ splines::ns(x, df = 4), linetype = 2) +
  labs(x = "Hours post inoculation", y = expression(log[2]*"(Normalized read counts)")) +
  # theme_gray() +
  scale_x_continuous(breaks = c(0, 2, 4, 8, 12, 18, 24)) +
```

```r
 guides(color = guide_legend(title = "Age")) +
 scale_color_discrete(labels = c("8 dpp", "16 dpp")) +
    cowplot::theme_cowplot(font_size = 10) +
   theme(strip.text = element_text(
       colour = "grey10",
       size = rel(0.8),
       margin = margin(0.8 * 7, 0.8 * 7, 0.8 * 7, 0.8 * 7)
       ),
       plot.margin = unit(c(0, 0, 0, 0), "cm"),
       axis.line = element_line(),
       legend.position = "none") +
   lemon::facet_rep_wrap(~ facetLabel, scales = "free", ncol = 1) +
   cowplot::panel_border()



gME <- ggplot_gtable(ggplot_build(pME))
stripr <- which(grepl('strip-t', gME$layout$name) & grepl("gTree", gME$grobs))

k <- 1

for (i in stripr) {
    j <- which(grepl('rect', gME$grobs[[i]]$grobs[[1]]$childrenOrder))
    gME$grobs[[i]]$grobs[[1]]$children[[j]]$gp$fill <- fills[k]
    k <- k+1
}

reorder_within <- function(x, by, within, fun = mean, sep = "___", ...) {
  new_x <- paste(x, within, sep = sep)
  stats::reorder(new_x, by, FUN = fun)
}

scale_x_reordered <- function(..., sep = "___") {
  reg <- paste0(sep, ".+$")
  ggplot2::scale_x_discrete(labels = function(x) gsub(reg, "", x), ...)
}


GO_ME <- tidyGOres %>%
  ggplot() +
  geom_col(aes(y = -log10(Fisher.weight01),
               x = reorder_within(Term,
                                  by = -log10(Fisher.weight01),
                                  moduleLabel),
               fill = color)) +
  scale_x_reordered(position = "top") +
  scale_fill_identity() +
  facet_wrap(~ moduleLabel, scales = "free", ncol = 1) +
  labs(x = "", y = expression(log[10]*"(p-value)")) +
    cowplot::theme_cowplot(font_size = 10) +
  theme(strip.background = element_rect(fill = "transparent", color = "transparent"),
        axis.line = element_blank(),
        strip.text = element_text(color = "transparent",
```

```
                                    size = rel(0.8),
                                    margin = margin(0.8 * 7, 0.8 * 7, 0.8 * 7, 0.8 * 7)),
         plot.margin = unit(c(0, 0, 0, 0), "cm"),
         axis.title.x = element_text(margin = margin(t = 5))) +
   coord_flip()

# cowplot::theme_cowplot(font_size = 10) +
#     theme(strip.text = element_text(
#         colour = "grey10",
#         size = rel(0.8),
#         margin = margin(0.8 * 7, 0.8 * 7, 0.8 * 7, 0.8 * 7)
#         ),
#         axis.line=element_line()


cowplot::plot_grid(gME, GO_ME, rel_widths = c(0.5, 1), align = "h", axis = "b")
```

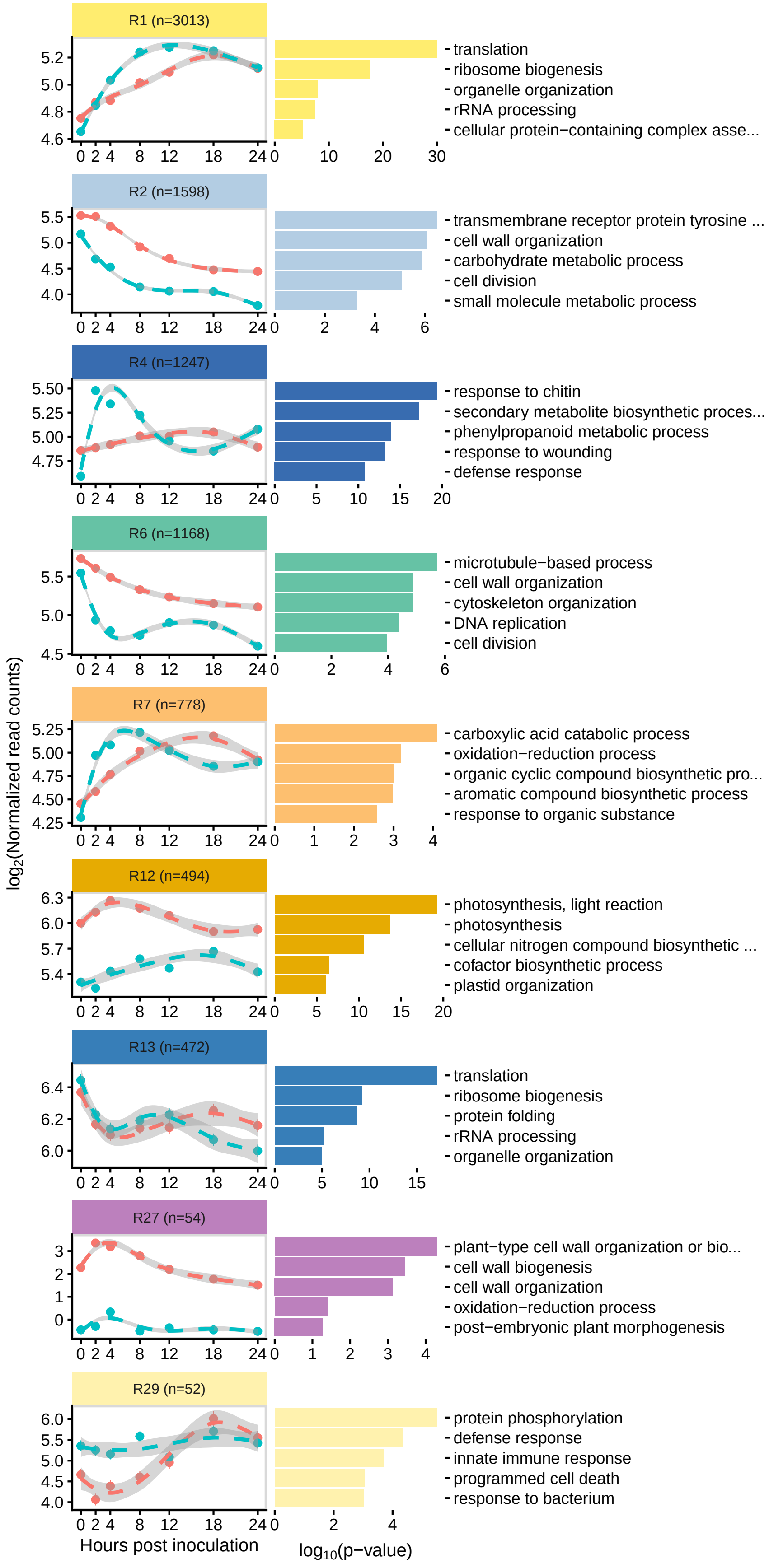

R1 (n=3013)
translation
ribosome biogenesis
organelle organization
rRNA processing
cellular protein−containing complex asse...
R2 (n=1598)
transmembrane receptor protein tyrosine ...
cell wall organization
carbohydrate metabolic process
cell division
small molecule metabolic process
R4 (n=1247)
response to chitin
secondary metabolite biosynthetic proces...
phenylpropanoid metabolic process
response to wounding
defense response
R6 (n=1168)
microtubule−based process
cell wall organization
cytoskeleton organization
DNA replication
cell division
R7 (n=778)
carboxylic acid catabolic process
oxidation−reduction process
organic cyclic compound biosynthetic pro...
aromatic compound biosynthetic process
response to organic substance
R12 (n=494)
photosynthesis, light reaction
photosynthesis
cellular nitrogen compound biosynthetic ...
cofactor biosynthetic process
plastid organization
R13 (n=472)
translation
ribosome biogenesis
protein folding
rRNA processing
organelle organization
R27 (n=54)
plant−type cell wall organization or bio...
cell wall biogenesis
cell wall organization
oxidation−reduction process
post−embryonic plant morphogenesis
R29 (n=52)
protein phosphorylation
defense response
innate immune response
programmed cell death
response to bacterium
log2(Normalized read counts)
Hours post inoculation
log10(p−value)

We can caluculate gene module membership ie the correlation between gene expression and the module eigengene

```
# module membership kME is the correlation to the module eigengene and represents the association to th
geneModuleMembership16 <- as.data.frame(cor(infection16dpp, MEs16Lables, use = "p"))
MMPvalue16 <- as.data.frame(corPvalueStudent(as.matrix(geneModuleMembership16), 21));
```

look for genes of interest in module 4 and 7:

Bring in contrast data from DESeq2 analysis comparing 16_Inoc_T2 vs 8_Inoc_T2

```
resT2 <- as.data.frame(lfcShrink(dds, contrast = c("condition", "16dpp_T2_Inoc", "8dpp_T2_Inoc"))) %>%
              mutate(gene = rownames(.))
resT2Cont <- as.data.frame(lfcShrink(dds, contrast = c("condition", "16dpp_T2_Inoc", "16dpp_T2_Cont")))
              mutate(gene = rownames(.))
```

```
resT4 <- as.data.frame(lfcShrink(dds, contrast = c("condition", "16dpp_T4_Inoc", "8dpp_T4_Inoc"))) %>%
              mutate(gene = rownames(.))
resT4Cont <- as.data.frame(lfcShrink(dds, contrast = c("condition", "16dpp_T4_Inoc", "16dpp_T4_Cont")))
              mutate(gene = rownames(.))
```

```
resT2 <- inner_join(resT2, resT2Cont, by = "gene", suffix = c("_T2vs8", "_T2vsC"))
resT4 <- inner_join(resT4, resT4Cont, by = "gene", suffix = c("_T4vs8", "_T4vsC"))

resultsEarly <- inner_join(resT2, resT4)
```

```
geneMod4 <-
  tibble(gene = colnames(infection16dpp),
         moduleLable = moduleLabels16) %>%
  mutate(
    modMembership = abs(geneModuleMembership16[gene, "ME4"]),
    modMemAdjPval = p.adjust(p = MMPvalue16$ME4, method = "BH") # [moduleLabels16 == 4]
  ) %>%
  left_join(resultsEarly)

geneMod7 <-
  tibble(gene = colnames(infection16dpp),
         moduleLable = moduleLabels16) %>%
  mutate(
    modMembership = abs(geneModuleMembership16[gene, "ME7"]),
    modMemAdjPval = p.adjust(p = MMPvalue16$ME7, method = "BH") # [moduleLabels16 == 7]
  ) %>%
    left_join(resultsEarly)
```

```
geneMod4_filt <- geneMod4 %>%
  mutate(passT2 = (padj_T2vs8 < 0.05 &
                     padj_T2vsC < 0.05 &
                     log2FoldChange_T2vs8 >= 1 &
                     log2FoldChange_T2vsC >= 1),
         passT4 = (padj_T4vs8 < 0.05 &
                     padj_T4vsC < 0.05 &
                     log2FoldChange_T4vs8 >= 1 &
                     log2FoldChange_T4vsC >= 1)
         ) %>%
  filter(passT2 == TRUE | passT4 == TRUE) %>%
  filter(modMembership >= 0.75) %>%
  #filter(modMembership >= quantile(geneMod4$modMembership, 0.75)) %>%
```

```
  dplyr::select(-contains("lfcSE"),
                -contains("stat"),
                -contains("pvalue"),
                -contains("baseMean")) %>%
  arrange(desc(modMembership)) %>%
  rowwise() %>%
  mutate(Annotation = paste(html_text(html_nodes(read_html(paste0("http://cucurbitgenomics.org/feature/g
    ) %>%
  separate(Annotation, into = c("Match Name", "E-value", "Identity", "Description"), sep = "__")

geneMod7_filt <- geneMod7 %>%
  mutate(passT2 = (padj_T2vs8 < 0.05 &
                     padj_T2vsC < 0.05 &
                     log2FoldChange_T2vs8 >= 1 &
                     log2FoldChange_T2vsC >= 1),
         passT4 = (padj_T4vs8 < 0.05 &
                     padj_T4vsC < 0.05 &
                     log2FoldChange_T4vs8 >= 1 &
                     log2FoldChange_T4vsC >= 1)
         ) %>%
  filter(passT2 == TRUE | passT4 == TRUE) %>%
  filter(modMembership >= 0.75) %>%
  #filter(modMembership >= quantile(geneMod4$modMembership, 0.75)) %>%
  dplyr::select(-contains("lfcSE"),
                -contains("stat"),
                -contains("pvalue"),
                -contains("baseMean")) %>%
  arrange(desc(modMembership)) %>%
  rowwise() %>%
  mutate(Annotation = paste(html_text(html_nodes(read_html(paste0("http://cucurbitgenomics.org/feature/g
    ) %>%
  separate(Annotation, into = c("Match Name", "E-value", "Identity", "Description"), sep = "__")


mod4and7GOIs <- bind_rows("Mod. R4" = geneMod4_filt, "Mod. R7" = geneMod7_filt, .id = "Module") %>%
    arrange(desc(modMembership)) %>%
    distinct(gene, .keep_all = TRUE) %>%
    arrange(Module, desc(modMembership))


kableExtra::kable(mod4and7GOIs %>%
                    dplyr::select(-`Match Name`, -`E-value`, -Identity, -Description), format="latex", b
  kable_styling(latex_options="scale_down")
```

Module	gene	moduleLable	modMembership	modMemAdjPval	log2FoldChange_T2vs8	padj_T2vs8	log2FoldChange_T2vsC	padj_T2vsC	log2FoldChange_T4vs8	padj_T4vs8	log2FoldChange_T4vsC	padj_T4vsC	passT2	passT4
Mod. R4	Csa3G706170	4	0.9600105	0.0000000	1.3898709	0.0029402	1.0593253	0.3940515	1.1399296	0.0239798	1.2171884	0.0485826	FALSE	TRUE
Mod. R4	Csa2G351540	4	0.9406524	0.0000004	1.3616668	0.0327769	2.1497868	0.0131642	0.4948059	0.5235623	2.5436807	0.0003579	TRUE	FALSE
Mod. R4	Csa3G845500	4	0.9380197	0.0000005	1.1012177	0.0061480	1.2105717	0.0498648	-0.0102767	0.9883439	0.5686060	0.3766573	TRUE	FALSE
Mod. R4	Csa3G782680	4	0.9246104	0.0000015	1.5068388	0.0000775	1.3838731	0.0116152	0.8281223	0.0582116	0.7758144	0.1816469	TRUE	FALSE
Mod. R4	Csa4G009900	4	0.9169922	0.0000026	1.8250168	0.0002261	1.9299967	0.0064796	0.6570751	0.2588914	2.2912030	0.0000406	TRUE	FALSE
Mod. R4	Csa5G524780	4	0.9140939	0.0000031	1.2371015	0.0433276	2.2021755	0.0066488	0.7709451	0.2597889	1.9153035	0.0078657	TRUE	FALSE
Mod. R4	Csa4G640960	4	0.9081028	0.0000051	1.5021135	0.0275712	2.5788916	0.0036529	-0.3130345	0.7307392	2.3283935	0.0021297	TRUE	FALSE
Mod. R4	Csa3G271380	4	0.9040334	0.0000066	1.7537840	0.0072234	2.1971300	0.0187068	-0.2687449	0.7661782	1.7768127	0.0347712	TRUE	FALSE
Mod. R4	Csa1G025960	4	0.9012187	0.0000077	1.1674401	0.0057501	1.5402297	0.0066488	-0.0530878	0.9362960	1.1137028	0.0383710	TRUE	FALSE
Mod. R4	Csa2G069200	4	0.8947864	0.0000112	1.6331850	0.0321952	2.7294085	0.0066488	-0.5669738	0.5465553	1.3857524	0.1924334	TRUE	FALSE
Mod. R4	Csa5G471600	4	0.8877080	0.0000173	1.4826746	0.0008038	1.9405516	0.0007089	-0.0874055	0.9016484	0.9185410	0.1495727	TRUE	FALSE
Mod. R4	Csa1G439830	4	0.8867294	0.0000182	1.6141040	0.0005676	1.7716575	0.0107740	1.3765261	0.0074272	1.6918006	0.0059780	TRUE	TRUE
Mod. R4	Csa1G086390	4	0.8815762	0.0000259	1.3412863	0.0024172	1.4832317	0.0272324	-0.5783553	0.2649027	1.4668518	0.0140072	TRUE	FALSE
Mod. R4	Csa4G411390	4	0.8773160	0.0000336	1.5769626	0.0088696	2.0508358	0.0207870	-0.1900954	0.8252679	1.4721343	0.0701171	TRUE	FALSE
Mod. R4	Csa3G219720	4	0.8749464	0.0000385	1.0142010	0.0012354	1.2465548	0.0066488	0.3525669	0.3673588	0.6780432	0.1615285	TRUE	FALSE
Mod. R4	Csa4G049610	4	0.8359295	0.0002425	1.7271720	0.0196407	2.3454202	0.0242726	0.5878543	0.5239993	2.4457724	0.0034939	TRUE	FALSE
Mod. R4	Csa6G367150	4	0.8080432	0.0007112	1.4268949	0.0063074	0.7363852	0.9997456	1.3328035	0.0126231	1.7390608	0.0060632	FALSE	TRUE
Mod. R7	Csa6G495000	7	0.9501139	0.0000001	1.6807367	0.0038978	0.4131100	0.9997456	1.4258427	0.0225707	1.9807971	0.0040090	FALSE	TRUE
Mod. R7	Csa6G213910	7	0.9414933	0.0000003	2.1449441	0.0003620	0.3153180	0.9997456	1.8827572	0.0035827	1.9741861	0.0076968	FALSE	TRUE
Mod. R7	Csa3G567330	7	0.9293113	0.0000007	1.7431791	0.0043160	2.5289635	0.0057249	-0.3364053	0.6690821	2.7319807	0.0002753	TRUE	FALSE
Mod. R7	Csa7G432140	7	0.9252129	0.0000010	1.0221420	0.0053574	-0.0479017	0.9997456	1.3115666	0.0002649	1.0393706	0.0173064	FALSE	TRUE
Mod. R7	Csa6G403620	7	0.9088402	0.0000036	1.2508920	0.0284654	0.4634498	0.9997456	1.1533441	0.0479600	1.7205324	0.0106230	FALSE	TRUE
Mod. R7	Csa2G070840	4	0.9030608	0.0000050	1.1974971	0.0076648	1.4792913	0.0242726	0.1655289	0.7898207	1.1374185	0.0526066	TRUE	FALSE
Mod. R7	Csa2G349630	7	0.8989141	0.0000066	3.9272827	0.0000000	1.2940690	0.4154108	1.2826325	0.0315018	3.2447890	0.0000001	FALSE	TRUE
Mod. R7	Csa6G454420	7	0.8743069	0.0000283	1.2303361	0.0300144	0.2987049	0.9997456	1.3393730	0.0130673	1.3635669	0.0438137	FALSE	TRUE
Mod. R7	Csa4G285730	1	0.8734286	0.0000296	2.3203664	0.0003727	0.1597129	0.9997456	1.7036611	0.0213874	1.9710580	0.0213608	FALSE	TRUE
Mod. R7	Csa6G517010	1	0.8656750	0.0000429	1.6339378	0.0315379	3.0025607	0.0346821	0.1919984	0.8481977	2.8248933	0.0034480	TRUE	FALSE
Mod. R7	Csa5G152820	7	0.8620518	0.0000507	1.7054469	0.0214431	1.5574696	0.5199601	1.6705263	0.0322478	2.2387310	0.0172186	FALSE	TRUE
Mod. R7	Csa6G501260	1	0.8551843	0.0000686	1.3755091	0.0209634	0.1235727	0.9997456	1.1799402	0.0450604	1.4858990	0.0394962	FALSE	TRUE
Mod. R7	Csa6G160180	1	0.8463722	0.0001033	0.7401645	0.1071162	1.0438717	0.2656611	1.7760578	0.0000230	1.5466169	0.0013964	FALSE	TRUE
Mod. R7	Csa6G431740	1	0.8315800	0.0001785	1.4421363	0.0191510	0.1356076	0.9997456	2.0566746	0.0004271	1.8116614	0.0086191	FALSE	TRUE
Mod. R7	Csa4G646190	1	0.8231660	0.0002280	1.1297512	0.0060468	0.3227404	0.9997456	1.0901826	0.0087498	1.6538751	0.0002312	FALSE	TRUE
Mod. R7	Csa2G270790	7	0.7918045	0.0006021	2.3212630	0.0104803	1.5130716	0.8083711	1.6459654	0.0262906	4.4452942	0.0003231	FALSE	TRUE
Mod. R7	Csa7G390060	1	0.7678290	0.0011451	0.6244622	0.3177034	0.0474841	0.9997456	1.3076313	0.0113320	1.3026763	0.0425571	FALSE	TRUE

```
write.csv(mod4and7GOIs, file = "Modules_4_7_GOIs.csv")

kableExtra::kable(mod4and7GOIs %>%
                    dplyr::select(Module, gene, `Match Name`, `E-value`, Identity, Description),
                  format="latex", booktabs=TRUE) %>%
  kable_styling(latex_options="scale_down")
```

Module	gene	Match Name	E-value	Identity	Description
Mod. R4	Csa3G706170	AT2G31945.1	1.2e-06	60.00	unknown protein
Mod. R4	Csa2G351540	AT5G43260.1	1.8e-34	65.63	chaperone protein dnaJ-related
Mod. R4	Csa3G845500	AT5G47910.1	0.0e+00	70.86	respiratory burst oxidase homologue D
Mod. R4	Csa3G782680	AT3G11820.1	4.5e-124	74.13	syntaxin of plants 121
Mod. R4	Csa4G009900	AT5G61760.1	9.7e-95	56.89	inositol polyphosphate kinase 2 beta
Mod. R4	Csa5G524780	AT5G14700.1	2.1e-88	48.35	NAD(P)-binding Rossmann-fold superfamily protein
Mod. R4	Csa4G640960	AT5G42830.1	2.2e-143	55.16	HXXXD-type acyl-transferase family protein
Mod. R4	Csa3G271380	AT1G13340.1	2.5e-56	37.40	Regulator of Vps4 activity in the MVB pathway protein
Mod. R4	Csa1G025960	AT2G38470.1	2.8e-119	47.19	WRKY DNA-binding protein 33
Mod. R4	Csa2G069200	AT2G30490.1	8.9e-30	63.83	cinnamate-4-hydroxylase
Mod. R4	Csa5G471600	AT1G14040.1	4.5e-309	66.50	EXS (ERD1/XPR1/SYG1) family protein
Mod. R4	Csa1G439830	AT1G30040.1	9.9e-117	63.55	gibberellin 2-oxidase
Mod. R4	Csa1G086390	AT2G48010.1	5.7e-211	62.46	receptor-like kinase in in flowers 3
Mod. R4	Csa4G411390	AT2G41640.1	2.7e-161	58.30	Glycosyltransferase family 61 protein
Mod. R4	Csa3G219720	AT1G11050.1	6.6e-234	65.27	Protein kinase superfamily protein
Mod. R4	Csa4G049610	AT4G11280.1	3.8e-186	65.29	1-aminocyclopropane-1-carboxylic acid (acc) synthase 6
Mod. R4	Csa6G367150	AT1G69040.2	1.3e-193	75.33	ACT domain repeat 4
Mod. R7	Csa6G495000	AT5G67400.1	1.0e-137	71.43	root hair specific 19
Mod. R7	Csa6G213910	AT5G05340.1	6.2e-116	65.62	Peroxidase superfamily protein
Mod. R7	Csa3G567330	AT5G13080.1	7.4e-44	71.79	WRKY DNA-binding protein 75
Mod. R7	Csa7G432140	AT1G80600.1	6.3e-175	74.75	HOPW1-1-interacting 1
Mod. R7	Csa6G403620	AT1G07040.1	1.0e-130	71.43	unknown protein
Mod. R7	Csa2G070840	AT1G08860.1	1.9e-227	68.62	Calcium-dependent phospholipid-binding Copine family protein
Mod. R7	Csa2G349630	AT1G67810.1	5.4e-53	55.22	sulfur E2
Mod. R7	Csa6G454420	AT5G06280.1	9.7e-14	38.26	unknown protein
Mod. R7	Csa4G285730	AT5G06720.1	6.0e-95	57.63	peroxidase 2
Mod. R7	Csa6G517010	AT2G29990.1	4.2e-64	80.99	alternative NAD(P)H dehydrogenase 2
Mod. R7	Csa5G152820	AT4G25700.1	2.0e-97	77.78	beta-hydroxylase 1
Mod. R7	Csa6G501260	AT4G39640.1	6.6e-209	64.92	gamma-glutamyl transpeptidase 1
Mod. R7	Csa6G160180	AT1G05010.1	3.2e-141	74.77	ethylene-forming enzyme
Mod. R7	Csa6G431740	AT5G19410.1	6.4e-213	59.39	ABC-2 type transporter family protein
Mod. R7	Csa4G646190	AT5G11950.1	1.1e-97	78.97	Putative lysine decarboxylase family protein
Mod. R7	Csa2G270790	AT4G35070.1	1.2e-35	37.04	SBP (S-ribonuclease binding protein) family protein
Mod. R7	Csa7G390060	AT4G02860.1	6.9e-93	59.15	Phenazine biosynthesis PhzC/PhzF protein

```
kableExtra::kable(mod4and7GOIs %>%
                  dplyr::select(Module, gene, modMembership, Description, -passT2, -passT4, -`Match
                format="latex", booktabs=TRUE, digits = 20) %>%
  kable_styling(latex_options="scale_down")
```

Module	gene	modMembership	moduleLable	modMemAdjPval	log2FoldChange_T2vs8	padj_T2vs8	log2FoldChange_T2vsC	padj_T2vsC	log2FoldChange_T4vs8	padj_T4vs8
Mod. R4	Csa3G706170	0.9600105	4	3.430158e-08	1.3898709	2.940210e-03	1.05932525	0.3940514824	1.13992964	0.0239797533
Mod. R4	Csa2G351540	0.9406524	4	4.167916e-07	1.3616668	3.277691e-02	2.14978676	0.0131641630	0.49480588	0.5235623271
Mod. R4	Csa3G845500	0.9380197	4	5.480415e-07	1.1012177	6.148018e-03	1.21057169	0.0498648135	-0.01027673	0.9883439439
Mod. R4	Csa3G782680	0.9246104	4	1.485907e-06	1.5068388	7.754051e-05	1.38387309	0.0116152162	0.82812232	0.0582115578
Mod. R4	Csa4G009900	0.9169922	4	2.585717e-06	1.8250168	2.261208e-04	1.92999668	0.0064795667	0.65707514	0.2588913696
Mod. R4	Csa5G524780	0.9140939	4	3.062732e-06	1.2371015	4.332757e-02	2.20217552	0.0066487908	0.77094506	0.2597889098
Mod. R4	Csa4G640960	0.9081028	4	5.120081e-06	1.5021135	2.757125e-02	2.57889158	0.0036529132	-0.31303452	0.7307392113
Mod. R4	Csa3G271380	0.9040334	4	6.632221e-06	1.7537840	7.223442e-03	2.19713004	0.0187067777	-0.26874492	0.7661782475
Mod. R4	Csa1G025960	0.9012187	4	7.653347e-06	1.1674401	5.750120e-03	1.54022975	0.0066487908	-0.05308777	0.9362960481
Mod. R4	Csa2G069200	0.8947864	4	1.124597e-05	1.6331850	3.219522e-02	2.72940853	0.0066487908	-0.56697381	0.5465552602
Mod. R4	Csa5G471600	0.8877080	4	1.731260e-05	1.4826746	8.037743e-04	1.94055156	0.0007089183	-0.08740545	0.9016483555
Mod. R4	Csa1G439830	0.8867294	4	1.823974e-05	1.6141040	5.675615e-04	1.77165753	0.0107740244	1.37652615	0.0074271878
Mod. R4	Csa1G086390	0.8815762	4	2.591417e-05	1.3412863	2.417233e-03	1.48323175	0.0272323673	-0.57835534	0.2649027018
Mod. R4	Csa4G411390	0.8773160	4	3.356186e-05	1.5769626	8.869603e-03	2.05083579	0.0207869976	-0.19009539	0.8252678743
Mod. R4	Csa3G219720	0.8749464	4	3.852191e-05	1.0142010	1.235416e-03	1.24655478	0.0066487908	0.35256687	0.3673588219
Mod. R4	Csa4G049610	0.8359295	4	2.425107e-04	1.7271720	1.964067e-02	2.34542015	0.0242726257	0.58785427	0.5239992763
Mod. R4	Csa6G367150	0.8080432	4	7.111629e-04	1.4268949	6.307412e-03	0.73638522	0.9997455713	1.33280352	0.0126230863
Mod. R7	Csa6G495000	0.9501139	7	1.068336e-07	1.6807367	3.897840e-03	0.41311001	0.9997455713	1.42584269	0.0225707044
Mod. R7	Csa6G213910	0.9414933	7	2.528006e-07	2.1449441	3.619690e-04	0.31531796	0.9997455713	1.88275725	0.0035826632
Mod. R7	Csa3G567330	0.9293113	7	7.264591e-07	1.7431791	4.316036e-03	2.52896350	0.0057249280	-0.33640533	0.6690821039
Mod. R7	Csa7G432140	0.9252129	7	1.023973e-06	1.0221420	5.357446e-03	-0.04790169	0.9997455713	1.31156664	0.0002648513
Mod. R7	Csa6G403620	0.9088402	7	3.611683e-06	1.2508920	2.846545e-02	0.46344979	0.9997455713	1.15334406	0.0479600430
Mod. R7	Csa2G070840	0.9030608	4	5.025251e-06	1.1974971	7.664827e-03	1.47929126	0.0242726257	0.16552892	0.7898207059
Mod. R7	Csa2G349630	0.8989141	7	6.615034e-06	3.9272827	4.186760e-10	1.29406905	0.4154108420	1.28263246	0.0315018244
Mod. R7	Csa6G454420	0.8743069	7	2.826254e-05	1.2303361	3.001439e-02	0.29870486	0.9997455713	1.33937296	0.0130673427
Mod. R7	Csa4G285730	0.8734286	1	2.962155e-05	2.3203664	3.726559e-04	0.15971293	0.9997455713	1.70366114	0.0213874463
Mod. R7	Csa6G517010	0.8656750	1	4.285083e-05	1.6339378	3.153789e-02	3.00256072	0.0346820738	0.19199843	0.8481977041
Mod. R7	Csa5G152820	0.8620518	7	5.070049e-05	1.7054469	2.144307e-02	1.55746963	0.5199600790	1.67052628	0.0322477640
Mod. R7	Csa6G501260	0.8551843	1	6.855180e-05	1.3755091	2.096341e-02	0.12357271	0.9997455713	1.17994024	0.0450604045
Mod. R7	Csa6G160180	0.8463722	1	1.033099e-04	0.7401645	1.071162e-01	1.04387167	0.2656611196	1.77605779	0.0000229799
Mod. R7	Csa6G431740	0.8315800	1	1.785270e-04	1.4421363	1.915103e-02	0.13560759	0.9997455713	2.05667456	0.0004270713
Mod. R7	Csa4G646190	0.8231660	1	2.280407e-04	1.1297512	6.046789e-03	0.32274039	0.9997455713	1.09018255	0.0087498062
Mod. R7	Csa2G270790	0.7918045	7	6.020873e-04	2.3212630	1.048026e-02	1.51307158	0.8083711230	1.64596538	0.0262905635
Mod. R7	Csa7G390060	0.7678290	1	1.145150e-03	0.6244622	3.177034e-01	0.04748414	0.9997455713	1.30763132	0.0113319883

gene	log2FoldChange_T4vsC	padj_T4vsC	passT2	passT4	Match Name	E-value	Identity	Description
Csa3G706170	1.2171884	4.858260e-02	FALSE	TRUE	AT2G31945.1	1.2e-06	60.00	unknown protein
Csa2G351540	2.5436807	3.578704e-04	TRUE	FALSE	AT5G43260.1	1.8e-34	65.63	chaperone protein dnaJ-related
Csa3G845500	0.5686060	3.766573e-01	TRUE	FALSE	AT5G47910.1	0.0e+00	70.86	respiratory burst oxidase homologue D
Csa3G782680	0.7758144	1.816469e-01	TRUE	FALSE	AT3G11820.1	4.5e-124	74.13	syntaxin of plants 121
Csa4G009900	2.2912030	4.061742e-05	TRUE	FALSE	AT5G61760.1	9.7e-95	56.89	inositol polyphosphate kinase 2 beta
Csa5G524780	1.9153035	7.865657e-03	TRUE	FALSE	AT5G14700.1	2.1e-88	48.35	NAD(P)-binding Rossmann-fold superfamily protein
Csa4G640960	2.3283935	2.129703e-03	TRUE	FALSE	AT5G42830.1	2.2e-143	55.16	HXXXD-type acyl-transferase family protein
Csa3G271380	1.7768127	3.477124e-02	TRUE	FALSE	AT1G13340.1	2.5e-56	37.40	Regulator of Vps4 activity in the MVB pathway protein
Csa1G025960	1.1137028	3.837098e-02	TRUE	FALSE	AT2G38470.1	2.8e-119	47.19	WRKY DNA-binding protein 33
Csa2G069200	1.3857524	1.924334e-01	TRUE	FALSE	AT2G30490.1	8.9e-30	63.83	cinnamate-4-hydroxylase
Csa5G471600	0.9185410	1.495727e-01	TRUE	FALSE	AT1G14040.1	4.5e-309	66.50	EXS (ERD1/XPR1/SYG1) family protein
Csa1G439830	1.6918006	5.978015e-03	TRUE	TRUE	AT1G30040.1	9.9e-117	63.55	gibberellin 2-oxidase
Csa1G086390	1.4668518	1.400716e-02	TRUE	FALSE	AT2G48010.1	5.7e-211	62.46	receptor-like kinase in in flowers 3
Csa4G411390	1.4721343	7.011708e-02	TRUE	FALSE	AT2G41640.1	2.7e-161	58.30	Glycosyltransferase family 61 protein
Csa3G219720	0.6780432	1.615285e-01	TRUE	FALSE	AT1G11050.1	6.6e-234	65.27	Protein kinase superfamily protein
Csa4G049610	2.4457724	3.493920e-03	TRUE	FALSE	AT4G11280.1	3.8e-186	65.29	1-aminocyclopropane-1-carboxylic acid (acc) synthase 6
Csa6G367150	1.7390608	6.063222e-03	FALSE	TRUE	AT1G69040.2	1.3e-193	75.33	ACT domain repeat 4
Csa6G495000	1.9807971	4.009014e-03	FALSE	TRUE	AT5G67400.1	1.0e-137	71.43	root hair specific 19
Csa6G213910	1.9741861	7.696816e-03	FALSE	TRUE	AT5G05340.1	6.2e-116	65.62	Peroxidase superfamily protein
Csa3G567330	2.7319807	2.752580e-04	TRUE	FALSE	AT5G13080.1	7.4e-44	71.79	WRKY DNA-binding protein 75
Csa7G432140	1.0393706	1.730635e-02	FALSE	TRUE	AT1G80600.1	6.3e-175	74.75	HOPW1-1-interacting 1
Csa6G403620	1.7205324	1.062303e-02	FALSE	TRUE	AT1G07040.1	1.0e-130	71.43	unknown protein
Csa2G070840	1.1374185	5.260656e-02	TRUE	FALSE	AT1G08860.1	1.9e-227	68.62	Calcium-dependent phospholipid-binding Copine family protein
Csa2G349630	3.2447890	8.437974e-08	FALSE	TRUE	AT1G67810.1	5.4e-53	55.22	sulfur E2
Csa6G454420	1.3635669	4.381373e-02	FALSE	TRUE	AT5G06280.1	9.7e-14	38.26	unknown protein
Csa4G285730	1.9710580	2.136078e-02	FALSE	TRUE	AT5G06720.1	6.0e-95	57.63	peroxidase 2
Csa6G517010	2.8248933	3.448031e-03	TRUE	FALSE	AT2G29990.1	4.2e-64	80.99	alternative NAD(P)H dehydrogenase 2
Csa5G152820	2.2387310	1.721862e-02	FALSE	TRUE	AT4G25700.1	2.0e-97	77.78	beta-hydroxylase 1
Csa6G501260	1.4858990	3.949623e-02	FALSE	TRUE	AT4G39640.1	6.6e-209	64.92	gamma-glutamyl transpeptidase 1
Csa6G160180	1.5466169	1.396397e-03	FALSE	TRUE	AT1G05010.1	3.2e-141	74.77	ethylene-forming enzyme
Csa6G431740	1.8116614	8.619114e-03	FALSE	TRUE	AT5G19410.1	6.4e-213	59.39	ABC-2 type transporter family protein
Csa4G646190	1.6538751	2.311617e-04	FALSE	TRUE	AT5G11950.1	1.1e-97	78.97	Putative lysine decarboxylase family protein
Csa2G270790	4.4452942	3.231415e-04	FALSE	TRUE	AT4G35070.1	1.2e-35	37.04	SBP (S-ribonuclease binding protein) family protein
Csa7G390060	1.3026763	4.255711e-02	FALSE	TRUE	AT4G02860.1	6.9e-93	59.15	Phenazine biosynthesis PhzC/PhzF protein

Plot all genes identified as early expressed. Supplemental figure 6.

```
moduleCount <-
  as.data.frame(counts(dds[colnames(infection16dpp), ], normalized = TRUE)) %>%
```

```r
  mutate(gene = rownames(.),
         module = moduleColors16.custom,
         moduleLabel = moduleLabels16) %>%
  gather("sample", "counts",-gene, -module, -moduleLabel) %>%
  mutate(age = rep(dds$age, each = ncol(infection16dpp)),
         timepoint = rep(str_remove(dds$timepoint, "T"), each =  ncol(infection16dpp)),
         treatment = rep(dds$treatment, each =  ncol(infection16dpp))
  ) %>%
  left_join(., as_tibble(table(moduleColors16.custom)), by = c("module" = "moduleColors16.custom")) %>%
  mutate(timepoint = as.numeric(timepoint),
         module = fct_infreq(module),
         facetLabel = fct_infreq(paste0(moduleLabel, " (n=", n,")"))
  )


geneplot <- moduleCount %>%
  # filter(gene %in% mod4and7GOIs$gene) %>%
  right_join(., mod4and7GOIs, by = "gene") %>%
  mutate(gene = fct_reorder(gene, .x = modMembership, .fun = "max", .desc = TRUE)) %>%
  ggplot() +
  stat_summary(
    fun.y = mean,
    geom = "line",
    aes(
      x = timepoint,
      y = counts,
      color = age,
      linetype = treatment
    )
  ) +
  stat_summary(fun.data = "mean_se", geom = "errorbar",
    aes(
      x = timepoint,
      y = counts,
      group = paste(age, treatment)
    ),
    color = rep(rep(c("#00BFC4", "#00BFC4", "#F8766D", "#F8766D"), each = 7),
                length(unique(mod4and7GOIs$gene)))
  ) +
  scale_x_continuous(breaks = c(0, 2, 4, 8, 12, 18, 24)) +
  labs(x = "Hours post inoculation", y = "Normalized read count") +
  cowplot::theme_cowplot(font_size = 10) +
    theme(strip.text =         element_text(
        colour = "grey10",
        size = rel(0.8) #,
        #margin = margin(0.8 * 7, 0.8 * 7, 0.8 * 7, 0.8 * 7)
        ),
        legend.position = "bottom") +
    facet_wrap(Module ~ gene, scales = "free", ncol = 5) +
    guides(linetype = guide_legend(title="Treatment"),
         color = guide_legend(title = "Age")) +
  scale_linetype_discrete(labels = c("Control", "Inoculated")) +
  scale_color_discrete(labels = c("8 dpp", "16 dpp")) +
```

```
  theme(legend.position=c(0.85, 0.05),
        legend.box = 'vertical',
        legend.margin = margin(t = -5)) +
    cowplot::panel_border()

g <- ggplot_gtable(ggplot_build(geneplot))
stripr <- which(grepl('strip-t', g$layout$name) & grepl("gTree", g$grobs))


# fills <- names(sort(table(moduleColors8.custom), decreasing = T))[unlist(rev(split(1:length(fills), c
#
fills <- names(sort(table(moduleColors16.custom), decreasing = T))[c(rep(7, 14), rep(4, 2), rep(7, 3), 

k <- 1

for (i in stripr) {
    j <- which(grepl('rect', g$grobs[[i]]$grobs[[1]]$childrenOrder))
    g$grobs[[i]]$grobs[[1]]$children[[j]]$gp$fill <- fills[k]
    k <- k+1
}
grid::grid.draw(g)
```

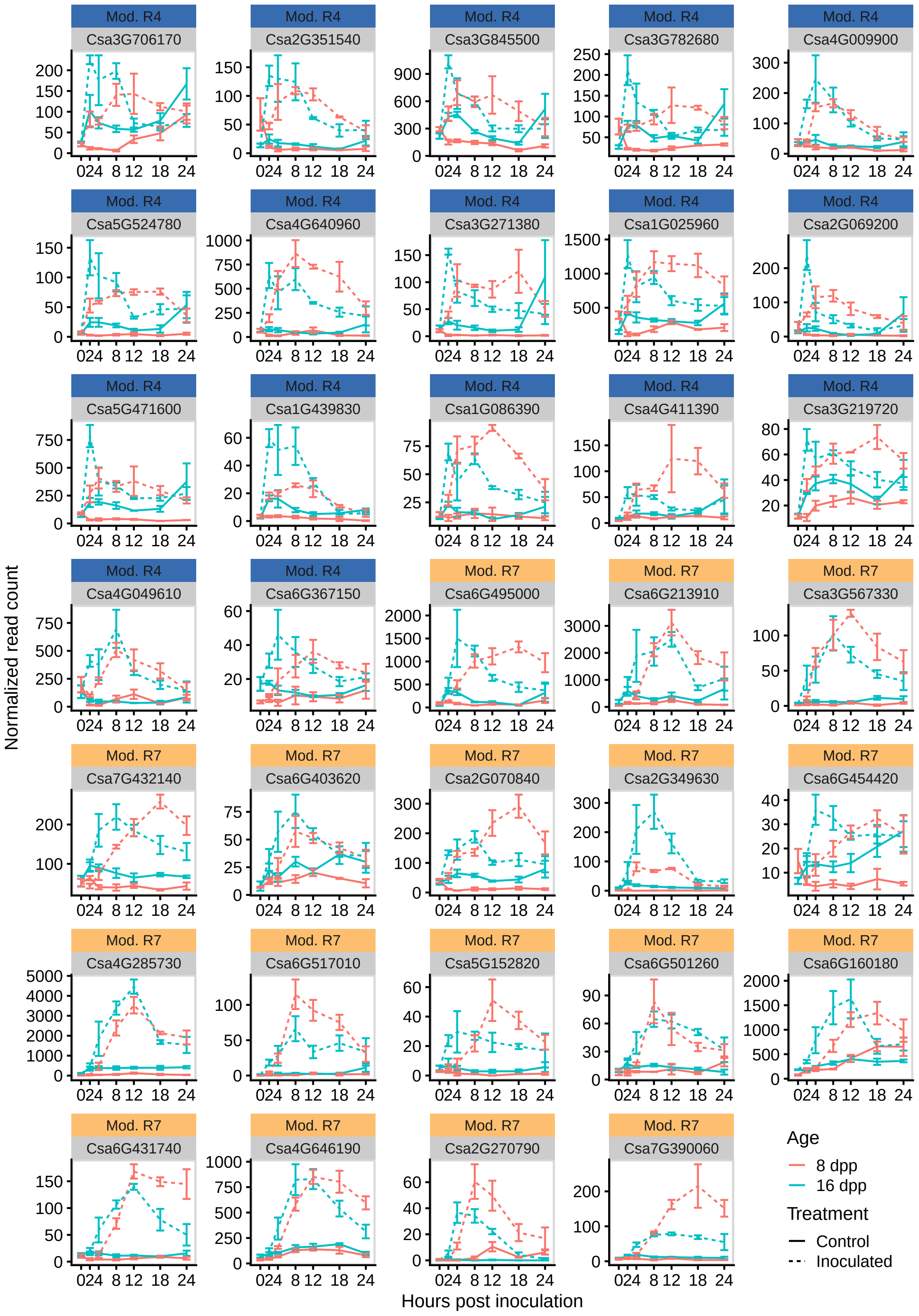

Mod. R4
Csa3G706170
Csa2G351540
Csa3G845500
Csa3G782680
Csa4G009900
Csa5G524780
Csa4G640960
Csa3G271380
Csa1G025960
Csa2G069200
Csa5G471600
Csa1G439830
Csa1G086390
Csa4G411390
Csa3G219720
Csa4G049610
Csa6G367150
Mod. R7
Csa6G495000
Csa6G213910
Csa3G567330
Csa7G432140
Csa6G403620
Csa2G070840
Csa2G349630
Csa6G454420
Csa4G285730
Csa6G517010
Csa5G152820
Csa6G501260
Csa6G160180
Csa6G431740
Csa4G646190
Csa2G270790
Csa7G390060
Normalized read count
Hours post inoculation
Age
8 dpp
16 dpp
Treatment
Control
Inoculated

Plot the subset of genes discussed in the discussion. Figure 9:

```
fd <- moduleCount %>%
    filter(gene %in% c("Csa1G025960", "Csa4G049610", "Csa6G160180",
                       "Csa4G285730", "Csa6G495000", "Csa3G845500",
                       "Csa3G271380", "Csa3G782680", "Csa6G431740",
                       "Csa1G086390", "Csa3G219720", "Csa4G009900",
                       "Csa2G069200", "Csa4G640960", "Csa4G411390")
    ) %>%
    mutate(
        gene = fct_relevel(
            gene,
            "Csa1G025960",
            "Csa4G049610",
            "Csa6G160180",
            "Csa4G285730",
            "Csa6G495000",
            "Csa3G845500",
            "Csa3G271380",
            "Csa3G782680",
            "Csa6G431740",
            "Csa1G086390",
            "Csa3G219720",
            "Csa4G009900",
            "Csa2G069200",
            "Csa4G640960",
            "Csa4G411390"
        ),
        `Annotation group` = fct_relevel(
            case_when(
                gene %in% c("Csa1G025960", "Csa4G049610", "Csa6G160180") ~ "Ethylene response",
                gene %in% c(
                    "Csa4G285730",
                    "Csa6G495000",
                    "Csa3G845500"
                ) ~ "Reactive oxygen metabolism",
                gene %in% c("Csa3G271380", "Csa3G782680", "Csa6G431740") ~ "Vesicle/Molecule transport"
                gene %in% c("Csa1G086390", "Csa3G219720", "Csa4G009900") ~ "Signal transduction",
                gene %in% c("Csa2G069200", "Csa4G640960", "Csa4G411390") ~ "Specialized metabolism"
            ),
            "Ethylene response",
            "Reactive oxygen metabolism",
            "Vesicle/Molecule transport",
            "Signal transduction",
            "Specialized metabolism"
        ),
        facet_order = case_when(
            gene == "Csa1G025960" ~ "Csa1G025960\nWRKY33",
            gene == "Csa4G049610" ~  "Csa4G049610\nACC synthase",
            gene == "Csa6G160180" ~ "Csa6G160180\nACC oxidase",
            gene == "Csa4G285730" ~ "Csa4G285730\nPeroxidase 2",
            gene == "Csa6G495000" ~  "Csa6G495000\nPeroxidase superfamily",
            gene == "Csa3G845500" ~  "Csa3G845500\nRespiratory burst oxidase homologue D",
            gene == "Csa3G271380" ~ "Csa3G271380\nIST1-LIKE 6",
```

```
            gene == "Csa3G782680" ~  "Csa3G782680\nSyntaxin 121",
            gene == "Csa6G431740" ~ "Csa6G431740\nABC-2 type transporter",
            gene == "Csa1G086390" ~  "Csa1G086390\nRLK IN FLOWERS 3",
            gene == "Csa3G219720" ~ "Csa3G219720\nProtein kinase superfamily",
            gene == "Csa4G009900" ~  "Csa4G009900\n inositol polyphosphate kinase",
            gene == "Csa2G069200" ~ "Csa2G069200\nCinnamate-4-hydroxylase",
            gene == "Csa4G640960" ~  "Csa4G640960\nHXXXD-type acyl-transferase",
            gene == "Csa4G411390" ~ "Csa4G411390\nGlycosyltransferase family 61"
        ),
        facet_order = factor(
            facet_order,
            levels = c(
                "Csa1G025960\nWRKY33",
                "Csa4G049610\nACC synthase",
                "Csa6G160180\nACC oxidase",
                "Csa4G285730\nPeroxidase 2",
                "Csa6G495000\nPeroxidase superfamily",
                "Csa3G845500\nRespiratory burst oxidase homologue D",
                "Csa3G271380\nIST1-LIKE 6",
                "Csa3G782680\nSyntaxin 121",
                "Csa6G431740\nABC-2 type transporter",
                "Csa1G086390\nRLK IN FLOWERS 3",
                "Csa3G219720\nProtein kinase superfamily",
                "Csa4G009900\n inositol polyphosphate kinase",
                "Csa2G069200\nCinnamate-4-hydroxylase",
                "Csa4G640960\nHXXXD-type acyl-transferase",
                "Csa4G411390\nGlycosyltransferase family 61"
            )
        )
    )

pGeneExp <- fd %>%
  ggplot() +
  stat_summary(
    fun.y = mean,
    geom = "line",
    aes(
      x = timepoint,
      y = counts,
      color = age,
      linetype = treatment
    )
  ) +
  stat_summary(fun.data = "mean_se", geom = "errorbar",
    aes(
      x = timepoint,
      y = counts,
      group = paste(age, treatment)
    ),
    color = rep(rep(c("#00BFC4", "#00BFC4", "#F8766D", "#F8766D"), each = 7), 15)
  ) +
  scale_x_continuous(breaks = c(0, 2, 4, 8, 12, 18, 24)) +
  labs(x = "Hours post inoculation", y = "Normalized read count") +
```

```
  cowplot::theme_cowplot(font_size = 10) +
    theme(strip.text =         element_text(
        colour = "grey10",
        size = rel(0.8) #,
        #margin = margin(0.8 * 7, 0.8 * 7, 0.8 * 7, 0.8 * 7)
        ),
        legend.position = "bottom") +
    facet_wrap(~ facet_order, ncol = 3,
                scales = "free", drop = FALSE) + #paste(gene, Description, sep = "\n")
    guides(linetype = guide_legend(title="Treatment"),
         color = guide_legend(title = "Age")) +
  scale_linetype_discrete(labels = c("Control", "Inoculated")) +
  scale_color_discrete(labels = c("8 dpp", "16 dpp")) +
  # theme(legend.position = c(0.75, 0.15),
  #       legend.box = 'vertical',
  #       legend.margin = margin(t = -5)) +
    cowplot::panel_border()

pGeneExp
```

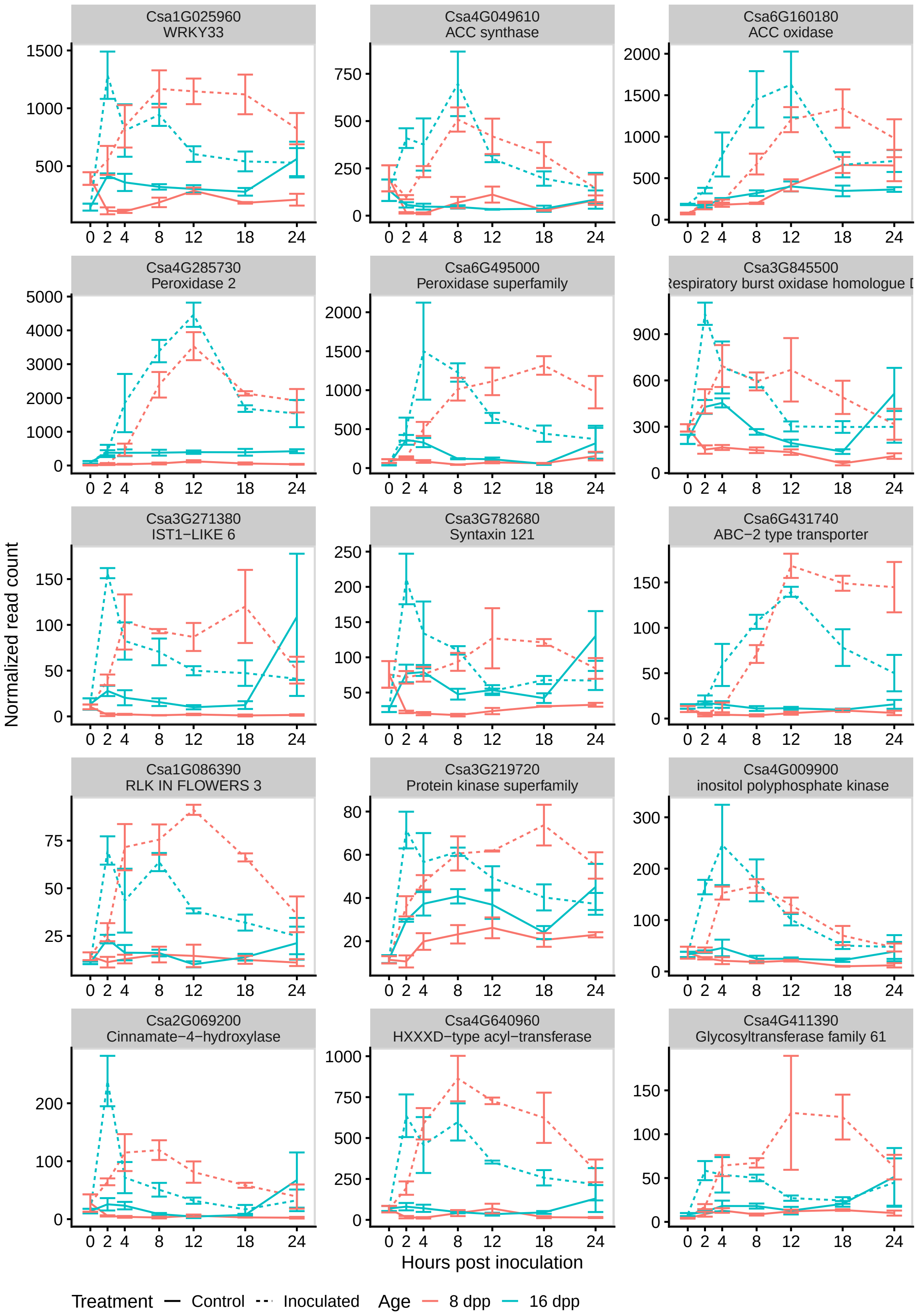

Csa1G025960
WRKY33
Csa4G049610
ACC synthase
Csa6G160180
ACC oxidase
Csa4G285730
Peroxidase 2
Csa6G495000
Peroxidase superfamily
Csa3G845500
Respiratory burst oxidase homologue D
Csa3G271380
IST1−LIKE 6
Csa3G782680
Syntaxin 121
Csa6G431740
ABC−2 type transporter
Csa1G086390
RLK IN FLOWERS 3
Csa3G219720
Protein kinase superfamily
Csa4G009900
inositol polyphosphate kinase
Csa2G069200
Cinnamate−4−hydroxylase
Csa4G640960
HXXXD−type acyl−transferase
Csa4G411390
Glycosyltransferase family 61
Normalized read count
Hours post inoculation
Treatment
Control
Inoculated
Age
8 dpp
16 dpp

```
library(grid)
library(gtable)
z <- ggplotGrob(pGeneExp)
z <- gtable_add_rows(z, unit(13/14, "line"), 6)
z <- gtable_add_grob(z,
                     list(rectGrob(gp = gpar(col=NA, fill = gray(0.80), size = 0.5)),
                          textGrob("Ethylene response",
                                   rot = 0, gp = gpar(col = gray(0),fontsize=12))),
                     7, 5, 7,14, name = paste(runif(2)))
z <- gtable_add_rows(z, unit(13/14, "line"), 12)
z <- gtable_add_grob(z,
                     list(rectGrob(gp = gpar(col=NA, fill = gray(0.80), size = 0.5,face="bold")),
                          textGrob("Reactive oxygen metabolism",
                                   rot = 0, gp = gpar(col = gray(0),fontsize=12))),
                     13, 5, 13, 14, name = paste(runif(2)))

z <- gtable_add_rows(z, unit(13/14, "line"), 18)
z <- gtable_add_grob(z,
                     list(rectGrob(gp = gpar(col=NA, fill = gray(0.80), size = 0.5)),
                          textGrob("Vesicle/Molecule transport",
                                   rot = 0, gp = gpar(col = gray(0),fontsize=12))),
                     19, 5, 19, 14, name = paste(runif(2)))
z <- gtable_add_rows(z, unit(13/14, "line"), 24)
z <- gtable_add_grob(z,
                     list(rectGrob(gp = gpar(col=NA, fill = gray(0.80), size = 0.5)),
                          textGrob("Signal transduction",
                                   rot = 0, gp = gpar(col = gray(0),fontsize=12))),
                     25, 5, 25, 14, name = paste(runif(2)))
z <- gtable_add_rows(z, unit(13/14, "line"), 30)
z <- gtable_add_grob(z,
                     list(rectGrob(gp = gpar(col=NA, fill = gray(0.80), size = 0.5)),
                          textGrob("Specialized metabolism",
                                   rot = 0, gp = gpar(col = gray(0),fontsize=12))),
                     31, 5, 31, 14, name = paste(runif(2)))

grid.newpage()
grid.draw(z)
```

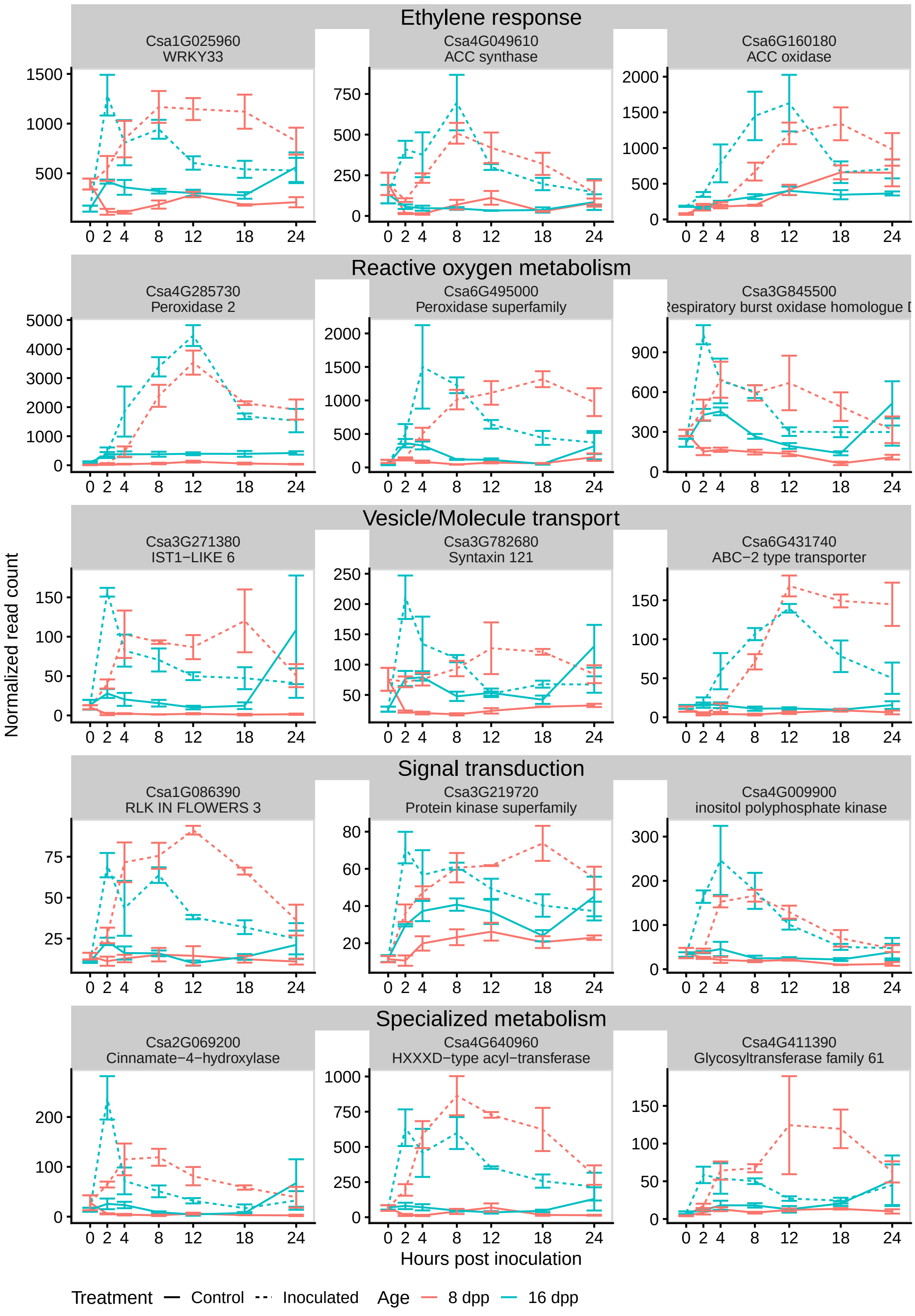
Ethylene response
Csa1G025960
WRKY33
Csa4G049610
ACC synthase
Csa6G160180
ACC oxidase
Reactive oxygen metabolism
Csa4G285730
Peroxidase 2
Csa6G495000
Peroxidase superfamily
Csa3G845500
Respiratory burst oxidase homologue D
Vesicle/Molecule transport
Csa3G271380
IST1−LIKE 6
Csa3G782680
Syntaxin 121
Csa6G431740
ABC−2 type transporter
Signal transduction
Csa1G086390
RLK IN FLOWERS 3
Csa3G219720
Protein kinase superfamily
Csa4G009900
inositol polyphosphate kinase
Specialized metabolism
Csa2G069200
Cinnamate−4−hydroxylase
Csa4G640960
HXXXD−type acyl−transferase
Csa4G411390
Glycosyltransferase family 61
Normalized read count
Hours post inoculation
Treatment
Control
Inoculated
Age
8 dpp
16 dpp

```r
pdf(file = "fig9.pdf", width = 7.5, height = 10)
    grid.draw(z)
dev.off()
```

```
## pdf
##   2
```

Plot the subset of genes discussed in the discussion from data in RNAseq exp1. Supp. Fig. 7:

```r
exp1Count <-
  as.data.frame(counts(dds_exp1, normalized = TRUE)) %>%
  mutate(gene = rownames(.),
         # module = moduleColors16.custom,
         # moduleLabel = moduleLabels16
         ) %>%
  gather("sample", "counts",-gene
         # , -module, -moduleLabel
          ) %>%
  mutate(age = rep(dds_exp1$age, each = nrow(dds_exp1)),
         timepoint = rep(str_remove(dds_exp1$timepoint, "T"), each =  nrow(dds_exp1)),
         timepoint = fct_relevel(timepoint, "0", "4", "24", "48")
  )

GOIs <- exp1Count %>%
    filter(gene %in% c("Csa1G025960", "Csa4G049610", "Csa6G160180",
                       "Csa4G285730", "Csa6G495000", "Csa3G845500",
                       "Csa3G271380", "Csa3G782680", "Csa6G431740",
                       "Csa1G086390", "Csa3G219720", "Csa4G009900",
                       "Csa2G069200", "Csa4G640960", "Csa4G411390")
    ) %>%
    mutate(
        gene = fct_relevel(
            gene,
            "Csa1G025960",
            "Csa4G049610",
            "Csa6G160180",
            "Csa4G285730",
            "Csa6G495000",
            "Csa3G845500",
            "Csa3G271380",
            "Csa3G782680",
            "Csa6G431740",
            "Csa1G086390",
            "Csa3G219720",
            "Csa4G009900",
            "Csa2G069200",
            "Csa4G640960",
            "Csa4G411390"
        ),
        `Annotation group` = fct_relevel(
            case_when(
                gene %in% c("Csa1G025960", "Csa4G049610", "Csa6G160180") ~ "Ethylene response",
                gene %in% c(
                    "Csa4G285730",
                    "Csa6G495000",
```

```
                    "Csa3G845500"
                ) ~ "Reactive oxygen metabolism",
                gene %in% c("Csa3G271380", "Csa3G782680", "Csa6G431740") ~ "Vesicle/Molecule transport"
                gene %in% c("Csa1G086390", "Csa3G219720", "Csa4G009900") ~ "Signal transduction",
                gene %in% c("Csa2G069200", "Csa4G640960", "Csa4G411390") ~ "Specialized metabolism"
            ),
            "Ethylene response",
            "Reactive oxygen metabolism",
            "Vesicle/Molecule transport",
            "Signal transduction",
            "Specialized metabolism"
        ),
        facet_order = case_when(
            gene == "Csa1G025960" ~ "Csa1G025960\nWRKY33",
            gene == "Csa4G049610" ~  "Csa4G049610\nACC synthase",
            gene == "Csa6G160180" ~ "Csa6G160180\nACC oxidase",
            gene == "Csa4G285730" ~ "Csa4G285730\nPeroxidase 2",
            gene == "Csa6G495000" ~  "Csa6G495000\nPeroxidase superfamily",
            gene == "Csa3G845500" ~  "Csa3G845500\nRespiratory burst oxidase homologue D",
            gene == "Csa3G271380" ~ "Csa3G271380\nIST1-LIKE 6",
            gene == "Csa3G782680" ~  "Csa3G782680\nSyntaxin 121",
            gene == "Csa6G431740" ~ "Csa6G431740\nABC-2 type transporter",
            gene == "Csa1G086390" ~  "Csa1G086390\nRLK IN FLOWERS 3",
            gene == "Csa3G219720" ~ "Csa3G219720\nProtein kinase superfamily",
            gene == "Csa4G009900" ~  "Csa4G009900\n inositol polyphosphate kinase",
            gene == "Csa2G069200" ~ "Csa2G069200\nCinnamate-4-hydroxylase",
            gene == "Csa4G640960" ~  "Csa4G640960\nHXXXD-type acyl-transferase",
            gene == "Csa4G411390" ~ "Csa4G411390\nGlycosyltransferase family 61"
        ),
        facet_order = factor(
            facet_order,
            levels = c(
                "Csa1G025960\nWRKY33",
                "Csa4G049610\nACC synthase",
                "Csa6G160180\nACC oxidase",
                "Csa4G285730\nPeroxidase 2",
                "Csa6G495000\nPeroxidase superfamily",
                "Csa3G845500\nRespiratory burst oxidase homologue D",
                "Csa3G271380\nIST1-LIKE 6",
                "Csa3G782680\nSyntaxin 121",
                "Csa6G431740\nABC-2 type transporter",
                "Csa1G086390\nRLK IN FLOWERS 3",
                "Csa3G219720\nProtein kinase superfamily",
                "Csa4G009900\n inositol polyphosphate kinase",
                "Csa2G069200\nCinnamate-4-hydroxylase",
                "Csa4G640960\nHXXXD-type acyl-transferase",
                "Csa4G411390\nGlycosyltransferase family 61"
            )
        )
    )


GOIs %>%
```

```r
ggplot() +
stat_summary(
  fun.y = mean,
  geom = "line",
  aes(
    x = timepoint,
    y = counts,
    color = age,
    group = age
  )
) +
stat_summary(fun.data = "mean_se", geom = "errorbar",
  aes(
    x = timepoint,
    y = counts,
    color = age,
    group = age
  ), width = 0.2
) +
# scale_x_continuous(breaks = c(0, 2, 4, 8, 12, 18, 24)) +
labs(x = "Hours post inoculation", y = "Normalized read count") +
cowplot::theme_cowplot(font_size = 10) +
  theme(strip.text =         element_text(
      colour = "grey10",
      size = rel(0.8) #,
      #margin = margin(0.8 * 7, 0.8 * 7, 0.8 * 7, 0.8 * 7)
      ),
      legend.position = "bottom") +
  facet_wrap(~ facet_order, ncol = 3,
             scales = "free", drop = FALSE) + #paste(gene, Description, sep = "\n")
  guides(color = guide_legend(title = "Age")) +
scale_linetype_discrete(labels = c("Control", "Inoculated")) +
scale_color_discrete(labels = c("8 dpp", "16 dpp")) +
# theme(legend.position = c(0.75, 0.15),
#       legend.box = 'vertical',
#       legend.margin = margin(t = -5)) +
  cowplot::panel_border()
```

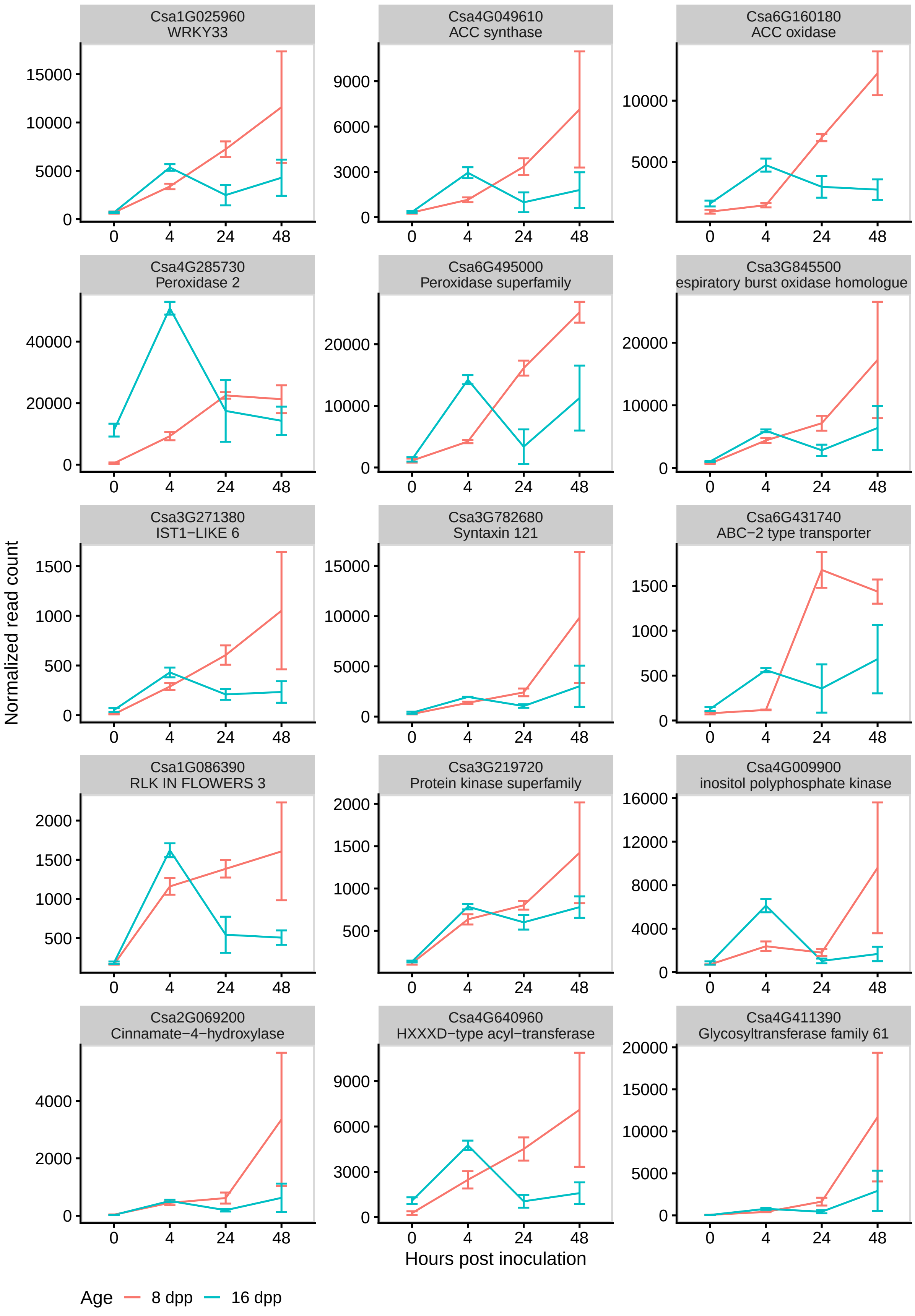
Csa1G025960
WRKY33
Csa4G049610
ACC synthase
Csa6G160180
ACC oxidase
Csa4G285730
Peroxidase 2
Csa6G495000
Peroxidase superfamily
Csa3G845500
espiratory burst oxidase homologue
Csa3G271380
IST1−LIKE 6
Csa3G782680
Syntaxin 121
Csa6G431740
ABC−2 type transporter
Csa1G086390
RLK IN FLOWERS 3
Csa3G219720
Protein kinase superfamily
Csa4G009900
inositol polyphosphate kinase
Csa2G069200
Cinnamate−4−hydroxylase
Csa4G640960
HXXXD−type acyl−transferase
Csa4G411390
Glycosyltransferase family 61
Normalized read count
Hours post inoculation
Age
8 dpp
16 dpp

Correlation of the subset of genes discussed in the discussion between Exp1 and 2. Supp. Fig. 8:

```
moduleCount %>%
    filter(timepoint %in% c(0, 4, 24),
           treatment == "Inoc" | timepoint == 0
           ) %>%
    filter(gene %in% c(
            "Csa1G025960",
            "Csa4G049610",
            "Csa6G160180",
            "Csa4G285730",
            "Csa6G495000",
            "Csa3G845500",
            "Csa3G271380",
            "Csa3G782680",
            "Csa6G431740",
            "Csa1G086390",
            "Csa3G219720",
            "Csa4G009900",
            "Csa2G069200",
            "Csa4G640960",
            "Csa4G411390"
            )
    ) %>%
    mutate(timepoint = as.factor(timepoint)) %>%
    group_by(gene, timepoint, age) %>%
    summarize(avgCount = mean(counts)) %>%
    left_join(GOIs %>%
    group_by(gene, timepoint, age) %>%
    summarize(avgCount = mean(counts)), by = c("gene", "timepoint", "age")) %>%
    ggplot(aes(x = log10(avgCount.x), y = log10(avgCount.y))) +
        geom_point() +
        geom_smooth(method = "lm") +
        ggpubr::stat_cor(method = "pearson") +
        labs(x = "log10(Avg. Counts) - Exp2", y = "log10(Avg. Counts) - Exp1") +
    cowplot::theme_cowplot(font_size = 11) +
    theme(strip.text = element_text(
        colour = "grey10",
        size = rel(0.8),
        margin = margin(0.5 * 7, 0.5 * 7, 0.5 * 7, 0.5 * 7)
        )
    ) +
    theme(legend.position="bottom",
        legend.box = 'vertical',
        legend.margin = margin(t = -5)) +
    cowplot::panel_border()
```

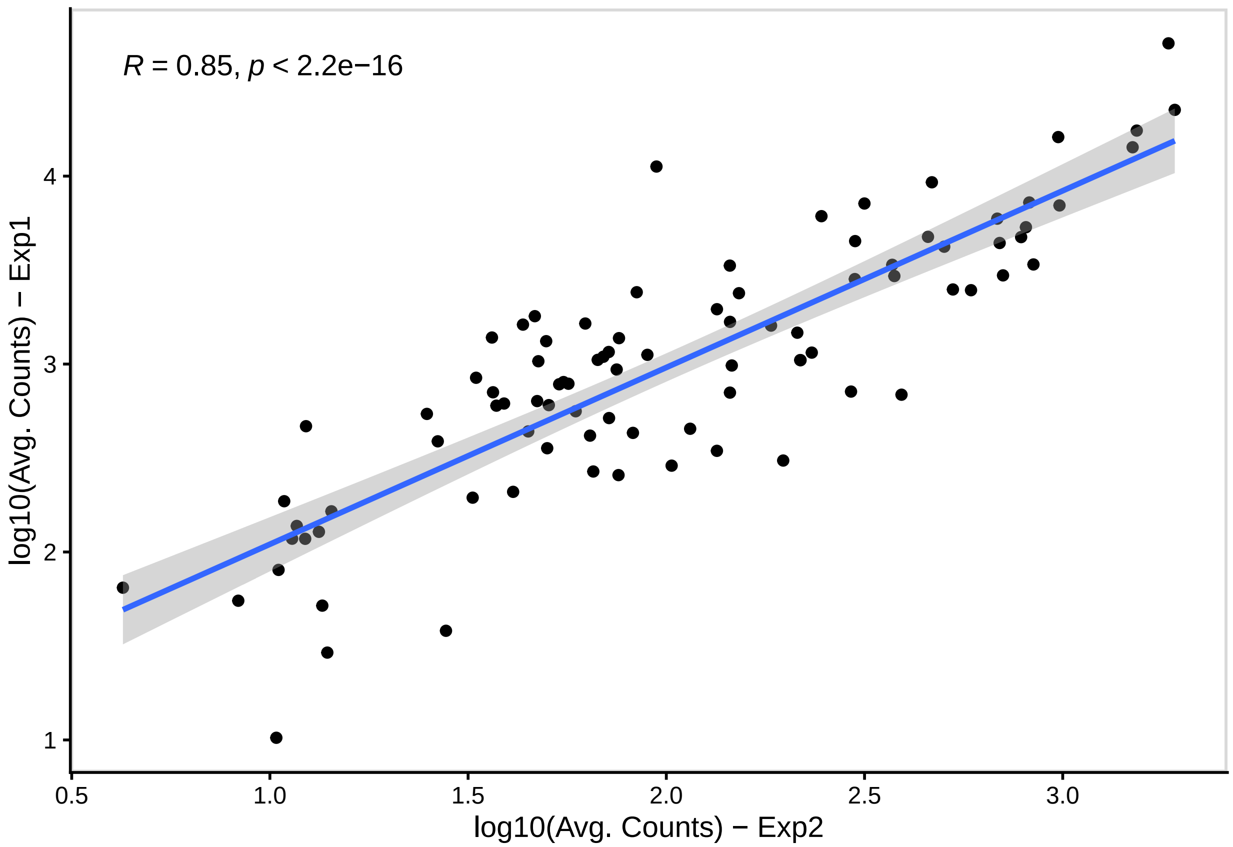


```r
sessionInfo()
```

```
## R version 3.6.1 (2019-07-05)
## Platform: x86_64-w64-mingw32/x64 (64-bit)
## Running under: Windows 10 x64 (build 19041)
##
## Matrix products: default
##
## Random number generation:
##  RNG:     Mersenne-Twister
##  Normal:  Inversion
##  Sample:  Rounding
##
## locale:
## [1] LC_COLLATE=English_United States.1252
## [2] LC_CTYPE=English_United States.1252
## [3] LC_MONETARY=English_United States.1252
## [4] LC_NUMERIC=C
## [5] LC_TIME=English_United States.1252
##
## attached base packages:
## [1] stats4    parallel  stats     graphics  grDevices utils     datasets
## [8] methods   base
##
## other attached packages:
##  [1] gtable_0.3.0                writexl_1.3
```

```
##  [3] rgeos_0.5-3                 sp_1.4-2
##  [5] ggdendro_0.1-20             magick_2.4.0
##  [7] readxl_1.3.1                rvest_0.3.6
##  [9] xml2_1.3.2                  RColorBrewer_1.1-2
## [11] topGO_2.38.1                SparseM_1.78
## [13] GO.db_3.10.0                AnnotationDbi_1.48.0
## [15] graph_1.64.0                pheatmap_1.0.12
## [17] WGCNA_1.69                  fastcluster_1.1.25
## [19] dynamicTreeCut_1.63-1       ggalluvial_0.10.0
## [21] rjson_0.2.20                kableExtra_1.1.0
## [23] DESeq2_1.26.0               SummarizedExperiment_1.16.1
## [25] DelayedArray_0.12.1         BiocParallel_1.20.1
## [27] matrixStats_0.56.0          Biobase_2.46.0
## [29] GenomicRanges_1.38.0        GenomeInfoDb_1.22.0
## [31] IRanges_2.20.1              S4Vectors_0.24.1
## [33] BiocGenerics_0.32.0         tximport_1.14.0
## [35] UpSetR_1.4.0                forcats_0.5.0
## [37] stringr_1.4.0               dplyr_1.0.0
## [39] purrr_0.3.4                 readr_1.3.1
## [41] tidyr_1.1.0                 tibble_3.0.3
## [43] ggplot2_3.2.1               tidyverse_1.3.0
##
## loaded via a namespace (and not attached):
##  [1] backports_1.1.8       Hmisc_4.4-0           plyr_1.8.6
##  [4] lazyeval_0.2.2        splines_3.6.1         digest_0.6.25
##  [7] foreach_1.5.0         htmltools_0.5.0       fansi_0.4.1
## [10] magrittr_1.5          checkmate_2.0.0       memoise_1.1.0
## [13] cluster_2.1.0         doParallel_1.0.15     annotate_1.64.0
## [16] modelr_0.1.8          jpeg_0.1-8.1          colorspace_1.4-1
## [19] blob_1.2.1            haven_2.3.1           xfun_0.16
## [22] crayon_1.3.4          RCurl_1.98-1.2        jsonlite_1.7.0
## [25] genefilter_1.68.0     impute_1.60.0         survival_3.2-3
## [28] iterators_1.0.12      glue_1.4.1            zlibbioc_1.32.0
## [31] XVector_0.26.0        webshot_0.5.2         scales_1.1.1
## [34] DBI_1.1.0             Rcpp_1.0.5            viridisLite_0.3.0
## [37] xtable_1.8-4          htmlTable_2.0.1       foreign_0.8-71
## [40] bit_1.1-15.2          preprocessCore_1.48.0 Formula_1.2-3
## [43] htmlwidgets_1.5.1     httr_1.4.2            acepack_1.4.1
## [46] ellipsis_0.3.1        pkgconfig_2.0.3       XML_3.98-1.20
## [49] nnet_7.3-12           dbplyr_1.4.4          locfit_1.5-9.4
## [52] tidyselect_1.1.0      rlang_0.4.7           munsell_0.5.0
## [55] cellranger_1.1.0      tools_3.6.1           cli_2.0.2
## [58] generics_0.0.2        RSQLite_2.2.0         broom_0.7.0
## [61] evaluate_0.14         yaml_2.2.1            knitr_1.29
## [64] bit64_0.9-7.1         fs_1.4.2              compiler_3.6.1
## [67] rstudioapi_0.11       png_0.1-7             reprex_0.3.0
## [70] geneplotter_1.64.0    stringi_1.4.6         lattice_0.20-38
## [73] Matrix_1.2-17         vctrs_0.3.2           pillar_1.4.6
## [76] lifecycle_0.2.0       data.table_1.13.0     bitops_1.0-6
## [79] R6_2.4.1              latticeExtra_0.6-29   gridExtra_2.3
## [82] codetools_0.2-16      MASS_7.3-51.4         assertthat_0.2.1
## [85] withr_2.2.0           GenomeInfoDbData_1.2.2 hms_0.5.3
## [88] grid_3.6.1            rpart_4.1-15          rmarkdown_2.3
## [91] lubridate_1.7.9       base64enc_0.1-3
```
